# A scoping review of health literacy in rare disorders: key issues and research directions

**DOI:** 10.1186/s13023-024-03332-5

**Published:** 2024-09-06

**Authors:** Una Stenberg, Lydia Westfal, Andreas Dybesland Rosenberger, Kristin Ørstavik, Maria Flink, Heidi Holmen, Silje Systad, Karl Fredrik Westermann, Gry Velvin

**Affiliations:** 1 Frambu Resource Center for Rare Disorders, Sandbakkveien 18, Siggerud, 1404 Norway; 2 Norwegian National Advisory Unit on Learning and Mastery in Health, Oslo University Hospital, Postboks 4959 Nydalen, Oslo, 0424 Norway; 3 National Neuromuscular Centre Norway, University Hospital of North-Norway, Hansine Hansens vei 37, Tromsø, 9019 Norway; 4 Section for Rare Neuromuscular Disorders and Unit for Congenital and Hereditary Neuromuscular Disorders (EMAN), Department of Neurology, Oslo University Hospital, Postboks 4950 Nydalen, Oslo, 0424 Norway; 5 Department of Neurobiology, Care Sciences and Society, Karolinska Institutet, Solnavägen 1, Solna, 171 77 Sweden; 6 Oslo Metropolitan University, Postbox 4, St. Olavs place, Oslo, N-0130 Norway; 7 National Centre for Rare Epilepsy-Related Disorders, Department of Rare Disorders, Division of Paediatric and Adolescent Medicine, Oslo University Hospital, Postboks, 4950 Nydalen, 0424 Oslo, Norway; 8 TRS Resource Centre for Rare Diseases, Sunnaas Rehabilitation Hospital, Bjørnemyrveien 11, 1453 Bjørnemyr, Norway

**Keywords:** Health literacy, Coping, Rare disorders, Rare disease, Rare genetic disorder, Rare developmental defect, Integrative model of health literacy, Scoping review

## Abstract

**Background:**

The ability to find, understand, appraise and utilise health information is crucial among individuals living with rare disorders. The aim of this study was to give a comprehensive overview of the literature on health literacy in adult persons with rare disorders.

**Methods:**

We applied a scoping review methodology and performed a systematic search in 2021 in bibliographic databases. Searches were conducted in Medline (Ovid), Embase (Ovid), PsycInfo (Ovid), CINAHL (ebsco), and ERIC (Ovid). References were sorted and evaluated for inclusion using EndNote and Covidence. This review was guided by the question “What are the characteristics of research on health literacy in rare disorders?”

**Results:**

The database searches yielded 75 eligible reports. A total of 6223 individuals with rare disorders were represented alongside 1707 caregivers. The reports in this review have included study participants representing a total of 80 different rare disorders with unique ORPHA and ICD-10 codes. The results revealed that persons with rare disorders often exhibit gaps in health literacy through a lack of knowledge and access to information related to self-management, their own diagnosis and health, as well as daily coping and social rights. In addition, the importance of aid and information from healthcare personnel and the significance of getting social support from others in the same situation were accentuated.

**Conclusion:**

This review emphasizes the importance of reinforcing health literacy among persons with rare disorders through peer support and education. This is the first review to give a comprehensive and state-of-the-art overview of literature investigating health literacy among persons with rare disorders and offers a basis for further research.

**Supplementary Information:**

The online version contains supplementary material available at 10.1186/s13023-024-03332-5.

## Introduction

In Europe, a disorder is considered rare when it affects less than 1:2000 individuals [1]. According to current calculations, more than 7000 different rare disorders have been identified. However, it is plausible that the actual number may be as high as 10,000 [2]. Although each rare disorder affects a limited quantity of individuals, it is estimated that the combined prevalence of all rare diseases is 3,5–5,9% [3]. Accordingly, up to 36 million people residing in the European Union are living with a rare disease [4]. Out of the total rare disorders, 72% have a genetic aetiology, and 70% have childhood onset [3]. Whilst there is a large clinical diversity between the rare disorders, they tend to have some aspects in common; they are known for being chronic, complicated, mostly degenerating, and often disabling [5].

Persons with rare disorders face some unique challenges in accessing information on their diagnosis, which may lead to issues in making beneficial health choices regarding treatment and care [6]. A key issue with rare disorders is the lack of research in the field [7, 8]. Insufficient evidence and knowledge on rare diseases in general pose challenges both for professionals and people with these diseases [8]. Due to healthcare professionals’ limited understanding of their rare disorder in general, as well as a lack of information provided, persons with rare disorders often need to search for health-related information themselves [6]. A systematic review published in 2017 aimed to provide an overview of adults` shared experience of living with a rare disorder, found that in 12 out of 21 reports, persons with rare disorders reported progressively becoming “experts” on their own diagnosis [9]. In some cases, those living with rare disorders possess more information about the condition than the healthcare professionals they encounter [10].

Healthcare systems are increasingly challenging to navigate [11]. Simultaneously, the healthcare services share prospective aims of prioritising digitization, enabling more home-based care, promoting shared decision-making, and ensuring equitable access to services [5, 12, 13]. Managing one’s health while dealing with a rare disorder and the responsibility of seeking information can be especially demanding due to the challenging standards set by the healthcare system [6].

Increased participation and responsibility for one’s own health impose a demand on the individual to have adequate health literacy. Health literacy pertains to individuals’ ability to manage the complex health requirements of today’s society and make informed decisions regarding health [14]. This includes understanding the factors that affect one’s health, addressing health challenges, and making appropriate health choices. There is a lack of consensus on the definition of health literacy, and multiple interpretations have been made [14]. A review by Sørensen et al. [15] identified as many as 17 different definitions of health literacy and created a working definition of health literacy by considering the contents of each interpretation. The inclusive definition according to Sørensen et al. is stated as follows:

*“Health literacy is linked to literacy and entails people’s knowledge*,* motivation and competencies to access*,* understand*,* appraise*,* and apply health information in order to make judgments and take decisions in everyday life concerning healthcare*,* disease prevention and health promotion to maintain or improve quality of life during the life course.” (ref p. 3).*

Along with the comprehensive definition, Sørensen et al. developed an integrated model of health literacy [15]. The model has been widely used to understand the complex interaction between individual skills and abilities related to health literacy, social and environmental factors, and health outcomes. The core elements of the model are four cognitive competencies; to access, understand, appraise, and apply health-related information. These four competencies allow a person to manoeuvre three identified domains on the health spectrum: healthcare, disease prevention, and health promotion. The model suggests that an individual’s ability to access and use health information is determined by their own skills, motivation, and knowledge as well as the social and environmental context they reside within. These conditions, accordingly, affect individuals’ ability to address their health and ultimately impact their health outcomes.

Sorensen’s model emphasises that components such as empowerment, health outcomes, and health behaviour are interlaced and connected to an individual’s health literacy. Enhancing the level of health literacy allows individuals to become more empowered and take charge of their health, participate in health-promoting behaviours, and gradually attain improved health outcomes [15]. Thus, participation and empowerment can give persons with rare disorders enhanced control over their own health and treatment, and increased involvement in decision-making processes that concern their health. This may lead to better health outcomes and elevated health-related quality of life, which remain crucial as persons with rare disorders report lower quality of life compared to those with more common chronic conditions [16]. They can feel stigmatised and marginalised in the healthcare system, and it can be challenging to find psychosocial support. Examining how to increase health literacy and empowerment for persons with rare disorders can therefore be an important and relevant direction for further research. Health literacy of individuals with rare disorders is an emerging field of research, and the literature is based on a wide range of study methodologies [7, 8]. Hence, this scoping review aims to give a comprehensive overview of empirical reports (from primary research studies) investigating health literacy among persons with rare disorders as reported in the international literature, by identifying characteristics of definitions, study populations, methods and interventions.

## Methods

### Study design and research questions

The scoping review process described by Arksey and O`Malley [17] aims to: “(…) *map rapidly the key concepts underpinning a research area and the main sources and types of evidence available and can be undertaken as a stand-alone project in their own right*,* especially where an area is complex or has not been reviewed comprehensively before.*” A scoping review methodology is also suitable for examining the extent, range, variety, and characteristics of evidence on a topic, but also to identify research gaps. This scoping review was conducted according to the five-stage framework by Arksey and O`Malley [17], enhanced by Levac [18] and Daudt [19] and reported according to the PRISMA Extension for Scoping Reviews [20] (shown in Additional file 1). A protocol for this review is available on request.

The aim of this review was to identify the characteristics of research on health literacy in rare disorders. The specific research questions were:

1. What are the characteristics of study populations?
2. When and where have reports on health literacy been carried out?
3. What are the characteristics of research questions used to investigate health literacy?
4. What are the characteristics of methods used to investigate health literacy?
5. What are the characteristics of assessment tools used to measure health literacy?
6. What are the characteristics of interventions that have been described in the reports?
7. How is health literacy defined or described in the reports?
8. How is access to health information and support for individuals with rare disorders described the reports?

### Overarching participatory approach

The study group in this scoping review included one co-researcher, one with experiential knowledge trained in research methods, several experienced healthcare professionals in the field of rare disorders, working in clinical practice (specialized health care), and experienced researchers in health literacy and scoping review methodology. All members have been involved in all stages of the review process.

### Eligibility criteria

This scoping review included primary research reports that investigated health literacy in adults with rare disorders. Reports were included if they had investigated the individual`s capacities, skills and motivation to make judgements and decisions in everyday life concerning healthcare, disease prevention and health promotion in persons with a rare disorder. While being 18 years of age or older was set as a search criterion, reports that included both adults and persons below 18 were not excluded. Empirical reports in English and Scandinavian languages published in peer-reviewed journals were included. All study designs were included. Dissertations, reports published in abstract form only, editorials, commentaries and duplicates were excluded.

### Systematic searches

In the first stage, research questions were developed by the study group in a highly iterative process. We agreed to apply a broad variety of synonyms, conducting many and extensive pilot searches and simultaneously enhancing the search strategy, and clarify the criteria for inclusion and exclusion of reports. A senior academic librarian, in close collaboration with the first author, developed a systematic literature search using MeSH-terms and free search terms combining a comprehensive set of synonyms and terms for health literacy and rare disorders. Both the librarian and the researchers in the study group had experience with previous literature searches in the field of rare disorders. The literature searches complied with the PICO principles and applied a combination of “OR” within groups and “AND” between groups. Searches were conducted in Medline (Ovid), Embase (Ovid), PsycInfo (Ovid), CINAHL (ebsco), and ERIC (Ovid) for publications between 2010 and 2021. No other sources for literature were searched for this review. The complete search strategy is displayed in Additional file 2.

### Selection of publications

All titles and abstracts were reviewed by the first author (US) and one of the co-authors independently using the systematic review software Covidence (Veritas Health Innovation). Disagreements and conflicts were resolved through discussion with a third review author.

### Data extraction

All data from the included reports were extracted according to study characteristics, participant characteristics included ORPHA and ICD-codes, description of interventions, methods, assessment tools, definitions and understanding of health literacy was collected using data extraction forms and reported separately for each study in evidence summaries (Supplementary Material 4–9: Tables 2–6). A full reference list of included reports is presented in Additional file 3. Extracted data is presented in a descriptive manner using text, tables and figures. All members of the study group participated in the data extraction. We did not attempt to contact the authors in this review process.

## Results

**Fig. 1:**
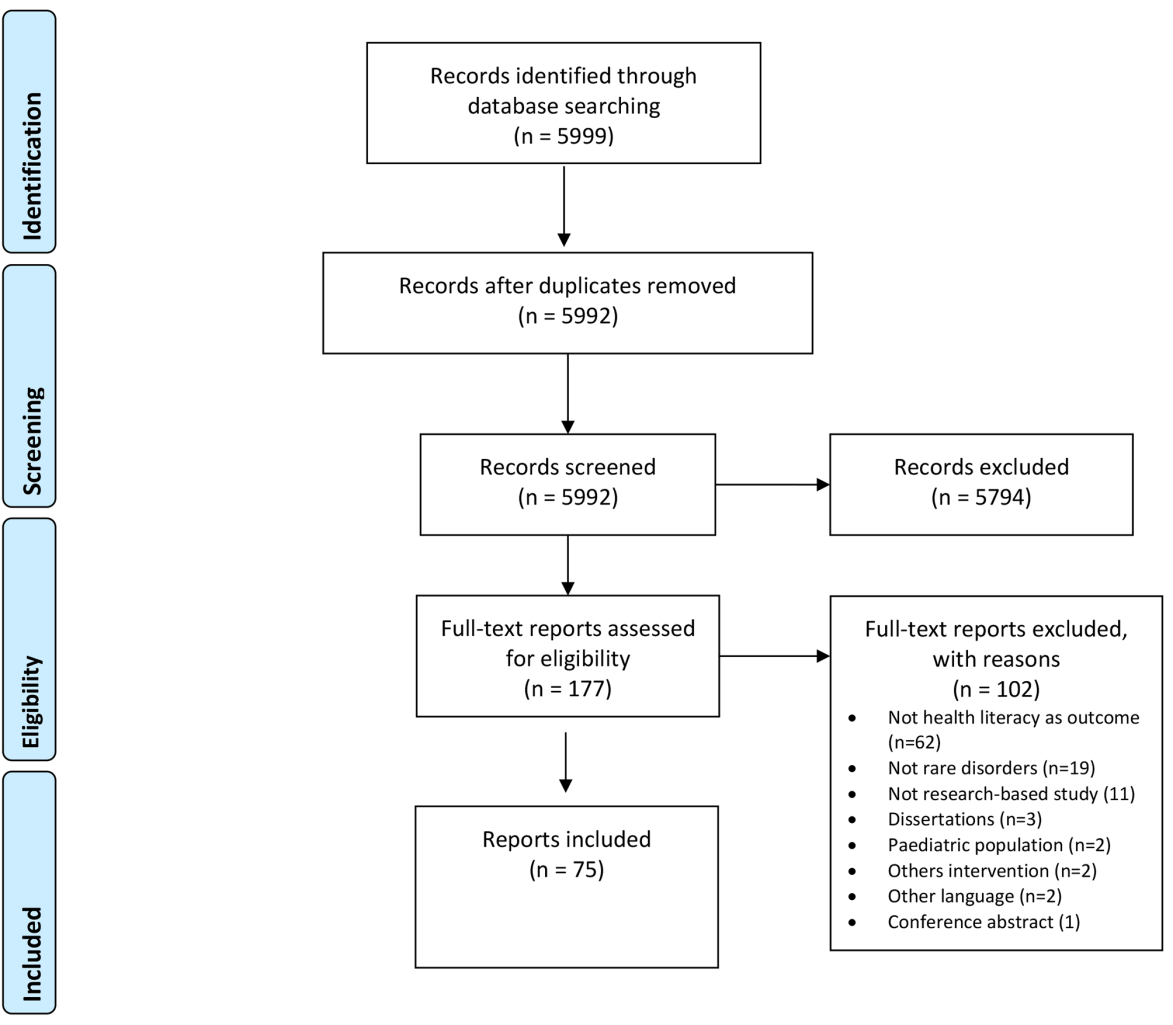
Prisma flow diagram

The search of the online databases resulted in 5999 reports when duplicates were removed. From these, 5794 were excluded because they did not fulfil the inclusion criteria. A total of 177 reports were downloaded in full text and read by two authors. Of these, 102 reports were excluded, leaving 75 to undergo analysis in this review (Fig. 1). All the included reports were in English language.

### Characteristics of study populations

A total of 6223 persons with a rare disorder and 1707 caregivers were represented in the 75 included reports. About 70% of the reports were based on data from samples with less than 100 participants. About 15% of the reports were based on samples with more than 200 participants. Of the included participants in the reports, about 60% were female. Of the reports that reported the mean age of the participants, approximately 75% of the participants were between 30 and 50 years of age. About 15% of the reports had participants with a mean age over 55 years, and eleven reports had participants with a mean age under 25 years.

The reports in this review have included study participants representing a total of 80 different rare disorders with unique ORPHA and ICD-10 codes. A detailed description of diagnoses is given in Table 1 (Additional file 4). Five of the reports included participants across rare disorders but did not specify what type of disorders. Most of the rare disorders had been investigated in one or two reports, but a few disorders were investigated in several reports: different types of Hemophilia were investigated in 24/75 reports, Cystic Fibrosis in 14/75 reports, Huntington’s disease in 7/75 reports, Scleroderma in 4/75 reports and Myotonic dystrophy type 1, Neurofibromatosis type 1 and Spina bifida in 3/75 reports.

### Where and when have reports on health literacy been carried out?

Of the 75 included reports, 21 were conducted in the USA, 11 in Canada and eight in the UK (see Table 2 for details in Additional file 9). The included reports were published between 2010 and 2021, 54/75 after 2016.

### Characteristics of research questions

The research questions most frequently investigated among the included reports were related to assessments of experienced knowledge and different health- and/or psychosocial outcomes (31/75). The second most investigated research questions (27/75) were about persons with rare disorders’ views, experiences and understanding of their own condition, care, health information, management, transition process or peer support (see Table 6 for details in Additional file 8). In addition, 16 reports were conducted to evaluate an intervention aimed to improve or strengthen participants` knowledge, health literacy or coping, and therefore included in this review.

### Characteristics of methodological design

Of the included reports, 28/75 applied a quantitative cross-sectional design to explore characteristics of patient groups in terms of knowledge and disease-related variables. Among the cross-sectional reports, both digital and paper-based surveys were used, and some gathered data through medical charts or personal interviews. In addition, fourteen reports applied an experimental design investigating either the feasibility or effects of specific interventions, mainly to increase knowledge or health literacy. Among the qualitative designs (28/75), individual interviews were frequently applied, less so focus groups. Most of the qualitative reports aimed to explore experiences and gain insight into the views of persons who are living with a rare disorder, for example, needs of information and support, barriers to care and communication with health care providers. To present the qualitative results, a thematic analysis approach was most frequently applied. A minority of reports (5/75) reported a mixed or multi-method approach, combining interviews and surveys (see Table 3 for more details, Additional file 5).

### Characteristics of assessment tools

Five of the assessment tools measured health literacy specifically. However, 23 standardized assessment tools aimed to assess important aspects relevant to health literacy, such as self-management skills, coping and medication adherence. Table 4 provides an overview of the standardised assessment tools used to measure health outcomes (Additional file 6). Quality of life was the outcome assessed most frequently (10/75) and was most commonly assessed with SF36 (4/75). Seven reports examined anxiety levels, while six estimated depression. Hospital Anxiety and Depression Scale (HAD) was the most commonly utilized tool to assess anxiety and depression (3/75). Correspondingly, 27 study-specific assessment tools sought to achieve outcomes closely related to health literacy, including health information-seeking patterns, medication information sources and knowledge, attitude and behaviour towards their condition. For a more detailed review of study-specific assessment tools, see Table 5 (Additional file 7).

### Characteristics of interventions

A total of 16/75 of the reports included interventions. Each intervention originated from a distinct study and had diverse characteristics in terms of study design, objectives, intended recipients, implementation settings, and delivery personnel, including healthcare professionals and peers. Additional information regarding this is provided in Table 6 (Additional file 8). The interventions encompassed both face-to-face approaches, such as individual sessions [21–27] and group-based patient education [22, 26, 28–34], and written information/online training [29, 31, 35–37]. The interventions took place in a variety of settings, including hospitals, clinics, and online platforms. The common thread between the interventions is that they all share the objective of enhancing patient outcomes and experiences through education, support, and empowerment. For example, they aim to improve knowledge, health literacy, and self-treatment skills, as well as to promote treatment adherence and reduce interruptions in care. In 9/16 interventions, the primary aim was to improve knowledge or understanding of the patient’s particular health condition or treatment. These nine interventions applied various components such as audiovisual materials, individualised training courses, or booklets. Out of those nine interventions, six demonstrated a significant (*p* < 0.05) improvement in knowledge of the targeted health condition or treatment [21, 25, 28, 32, 36, 37].

Out of all interventions, 5/16 aimed predominantly at reducing psychiatric symptoms, such as depression, anxiety, and somatic symptom severity. Several interventions displayed positive effects on mental health, including improvements in emotional health, coping strategies, and quality of life [22, 26, 28, 29, 32, 33, 35]. Examples of such interventions included group counselling and group mindfulness training. The interventions were evaluated using methods such as self-report questionnaires, physiological measures, and clinical assessments. The outcomes measured included improvements in physical health, mental health, quality of life, and social support.

### Description of health literacy

Only 6/75 reports described in the introduction how they defined health literacy [21, 38–42]. Five of these reports were based on the understanding and definition of health literacy as the cognitive and social skills that determine the motivation and ability of individuals to gain access to, understand and use information in ways that promote and maintain good health (WHO). One of the reports defined health literacy as “the patients’ skills on reading, listening, analysing decisions making and applying these skills to the situation related to health monitoring and coordination for strategy plan in term of health promotion” [42].

### Access to health information and support

Most of the reports included in this review investigated knowledge or understanding of one’s own health and diagnosis, and access to health information. Persons with rare disorders commonly lack information about:

- Own diagnosis and health [43–58].
- Self-management and daily coping [6, 10, 54, 59–63].
- Medication, treatment options and research-based recommendations [6, 10, 28, 51, 64–67].
- Peer and professional support [53–55].
- Clinical trials and research [53–55].
- Sexual knowledge [68–71].
- Behaviour and attitude [28, 72, 73].
- Social rights [28, 60].
- Pregnancy and childbirth [51, 60].
- Ageing [71].
- Navigation and coordination [23].

The most important sources of health information summarized among the included reports were physicians, the internet, patient organizations and spouse/partner [74–76]. Transitions in life can be challenging and generate new needs for information and care. Three of the reports investigated the transition process from paediatric to adult services [23, 77, 78]. Persons with rare disorders and their family caregivers call for health information on various aspects of the disease burden including medical research and treatment, coping strategies, management, symptoms and general knowledge about the disease [57, 63].

Only a few reports investigated how persons with rare disorders are navigating in healthcare and their experiences of healthcare services. These reports found that many persons with rare disorders feel let down by the system- and lack trust in the standards of health care [54, 79–81]. Several reports described the frustration among persons with rare disorders because of a lack of knowledge about diagnosis and medication by healthcare professionals [54, 56, 59, 62, 73, 81, 82] and concerns about poor communication and information provision [83].

Some of the reports described the experiences of persons with rare disorders concerning limited access to peer- and professional support, like specialized care, treatment plans and access to peer groups [34, 53, 61, 62, 84–86]. Persons with rare disorders missed the engagement in health care to assist in their management of the disease [85], and one report claimed that hospital visits could be reduced with more information [52].

Several reports have investigated peer support [6, 22, 28, 44, 50, 81, 82, 87–89]. Persons with rare disorders who connected and interacted with fellow individuals with rare disorders reported great improvements in overall health, disease severity, motivation to take care of health, emotional well-being and satisfaction with their primary treating physician [66, 69].

## Discussion

This scoping review identified 75 reports presenting data on rare disorders and aspects of health literacy, thereby providing valuable insight into the characteristics of research in the field of health literacy in individuals with rare disorders. A total of 6223 individuals with rare disorders and 1707 caregivers were included, and 80 different rare disorders were represented. Most of the studies were published after 2016, and were conducted in the USA, Canada and UK. The most frequently investigated research questions were related to different health- and psychosocial outcomes, understanding of own condition, health information and support, or concerning evaluation of an intervention. The reports used a variety of research methodologies, including qualitative, quantitative, and mixed methods approaches. Cross-sectional designs were frequently employed to depict patient characteristics, knowledge and health-related variables, and qualitative designs were commonly used to capture the perspectives of persons living with rare disorders. In total 23 standardized assessment tools and 27 study-specific assessment used in the reports. Only five assessment tools measured health literacy specifically. Some of the reports also assessed interventions to improve elements such as knowledge, health literacy and coping strategies. These interventions encompassed both face-to-face approaches, such as individual sessions and group-based patient education.

Only six reports had described how they defined health literacy. Five of these reports were based on the understanding and WHO-definition of health literacy as the cognitive and social skills that determine the motivation and ability of individuals to gain access to, understand and use information in ways that promote and maintain good health (WHO). Concerning access to health information and support, the results revealed that individuals with rare disorders often exhibit gaps in knowledge and access to information related to self-management, their own diagnosis and health, as well as daily coping and social rights. In addition, the importance of aid and information from healthcare personnel and the significance of getting social support from others in the same situation were accentuated.

A recurring issue identified among the reports was that individuals with rare disorders consistently encounter challenges in accessing information on their own health and diagnosis, self-managing and coping [43–58]. This observation has been established in previous research and can sometimes be ascribed to a lack of knowledge among healthcare personnel [6, 9, 10]. The understanding and appraisal of health information could pose difficulties since the information available on rare conditions often is complex and contains medical terminology that is challenging to comprehend. This particularly applies to those with cognitive impairments, which pertains to 44% of the rare disease population [90]. More than 7000 rare disorders are identified, and only 80 of these disorders are represented in this review. More than 50% of the included reports have included study participants with Haemophilia, Cystic Fibrosis and Huntington’s disease, which means that a range of different rare disorders have not been included in health literacy research. A majority of the interventions in this study focused on increasing knowledge and understanding of one’s own health and treatment. Acquiring the skills to apply health knowledge to everyday life efficiently can profoundly impact health outcomes and is especially important when it comes to self-management, such as adherence to medication and treatment [15, 91–95].

Another possible challenge related to access to information and support is the often-large geographical distances between persons with rare disorders. This may result in difficulties when it comes to meeting or participating in peer-support groups in person [96]. Peer interactions appear particularly important in this population [6, 53–55], and several of the perceived benefits of the interventions in this review were associated with the recognition, acceptance and companionship encountered within peer-support groups [16, 28, 32, 35]. Interestingly, none of the included reports explored the potential benefits of online peer support, which has been found to be an effective supplement to in-person meetings in people with other disorders.

While there is reason to believe that health literacy has a significant impact on health outcomes, only one of the included reports investigated this possible correlation, finding that individuals who possessed adequate health literacy displayed more favourable health-related outcomes [38]. In that report, the authors observed that individuals who possessed adequate health literacy displayed more favourable health-related outcomes. None of the included reports explored health literacy across various types of rare disorders. One prominent finding across the reviewed reports is the shortage of accessible health information specifically targeted towards individuals with rare conditions. There is a need to investigate if there are structural or social barriers that limit access to information and support for the population. Furthermore, it would be valuable to examine the underlying factors that impact health literacy in persons with rare disorders, including the association between health literacy and socio-demographic variables, health status, self-efficacy and health-related quality of life. Another potential research topic could be to evaluate the success of interventions aiming at improving health literacy in persons with rare disorders and their caregivers.

To the best of our knowledge, only four previous reports have explicitly aimed to examine the levels of health literacy in persons with rare disorders [38–40, 42]. Furthermore, the data does not provide enough information to say anything about relatives’ health literacy. Enhancing health literacy is known to be an enabler for improved empowerment and participation, which is associated with positive health outcomes [13, 94]. Empowerment is especially important in the field of rare disorders, due to the unique challenges of low prevalence, limited knowledge and expertise, and compromised quality of life [94].

To achieve a better understanding of health literacy in rare disorders, we could benefit from the incorporation of different perspectives, including those of persons with rare disorders, their family members and healthcare providers. We need future research on how different dimensions of health literacy, and interventions aiming to strengthen health literacy, influence health outcomes according to health care, disease prevention and health promotion. We need to achieve a deeper understanding of how the personal determinants of health literacy, such as individual skills and motivation, interact with situational determinants, such as social and environmental factors, to shape health outcomes. To properly address the executive challenges faced by persons with rare disorders we need a greater understanding of health literacy in rare disorders [28]. The integrated model of health literacy [15] can serve as a tool to point us in the right direction when designing future research projects.

The strength of our work lies in providing a comprehensive overview of the reported findings from research on health literacy in rare disorders. We conducted an up-to-date systematic search in five databases without restrictions. Despite using an array of synonyms in database searches to maximise the identification of relevant reports, the search terms used are not exhaustive. Hence, some reports may not have been detected. To reduce the risk of selection bias, two authors independently assessed the abstracts and reports in full text according to the a priori eligibility criteria. Further, in line with the scoping review framework, we have not evaluated the methodological quality or risk of bias among the included reports. This may be seen as a limitation; however, the purpose of scoping reviews is to give an overview of the available research literature, characterise a research area and pinpoint gaps in knowledge that should be addressed in future systematic reviews.

This review has important implications for practice. Healthcare does not offer curative treatment options for most rare disorders, and several reports suggest the development of consensus recommendations for care. To optimise health and secure continuity of care several reports included in this review recommend formalisation of the transition process through the courses of illness and life. Moreover, the results reveal that some of the key challenges for persons with rare disorders are related to important aspects of health literacy, such as accessing, understanding, and applying health information. Our findings indicate a need for strengthened health literacy in the rare disease population, that could be accomplished by developing health communication strategies tailored to the needs and preferences of persons with rare conditions. Healthcare personnel can play a significant role in enhancing health literacy, which is an additional implication for practice. Health care personnel can achieve this by offering clear and understandable health-related information and encouraging an active dialogue between patients and professionals. Another way for healthcare personnel to assist persons with rare disorders is by offering them the support needed to accept, cope, and effectively manage their condition [97].

## Conclusion

This scoping review consists of 75 reports presenting data on rare disorders and aspects of health literacy, thereby providing valuable insight into the characteristics of research in the field of health literacy in individuals with rare disorders. In total, 6223 individuals with rare disorders and 1707 caregivers were included, and 80 different rare disorders were represented. Most of the studies were published after 2016, and were conducted in the USA, Canada and UK.

The findings of this scoping review demonstrate that persons with rare disorders experience considerable gaps in knowledge and information, particularly in relation to their own diagnosis and health, treatment options, self-management and coping strategies. Moreover, the lack of diagnosis-specific knowledge and limited information provided by healthcare professionals are identified as a common concern among persons with rare disorders. Access to, and understanding, health information is key aspects of health literacy. Therefore, our results imply a need for increased awareness regarding the state of health literacy among individuals with rare disorders. The points of view expressed in this review offer valuable perspectives that can help health personnel in outlining the communicative strategy when caring for individuals with rare disorders.

This review provides a solid understanding block for future research into the emerging field of health literacy in rare disorders, by examining the challenges that persons with rare conditions encounter. Moreover, the findings enable us to develop a better understanding of the care and support persons with a rare disorder and their family members require.

These results pave the way for future research that looks to improve the healthcare experience of those with rare disorders and their caretakers and shed light on the importance of empowering the rare disease population through peer support, participation, education and increased health literacy. Future reports in this field are necessary to develop strategies and interventions that improve health literacy and enhance health outcomes and the quality of life for individuals with rare disorders.

## Electronic supplementary material

Below is the link to the electronic supplementary material.

Supplementary Material 1

Supplementary Material 2

Supplementary Material 3

Supplementary Material 4

Supplementary Material 5

Supplementary Material 6

Supplementary Material 7

Supplementary Material 8

Supplementary Material 9

## Data Availability

All data generated or analysed during this review are included in this published article (and its additional files).
