# Supplementary material for: A scoping review of health literacy in rare disorders: key issues and research directions
Source: Orphanet J Rare Dis. 2024 Sep 6;19:328. doi: 10.1186/s13023-024-03332-5 (PMC11380335; doi:10.1186/s13023-024-03332-5)
Supplement: Supplementary file 2 — Supplementary Material 2 [file 13023_2024_3332_MOESM2_ESM.docx]

Documentation of literature search

Documentation of literature search for Una Stenberg.

| **Database** | **Number of studies** |
| --- | --- |
| Medline (Ovid): | 3252 |
| Embase (Ovid): | 4083 |
| PsycInfo (Ovid): | 682 |
| CINAHL (Ebsco): | 1565 |
| ERIC (Ovid): | 22 |
| Number of references before dublets | 9604 |
| Number of references after dublets were removed | 5999 |

All the searches are conducted 20.04.2021 by:

**Hilde Iren Flaatten, Medical Librarian
University of Oslo : Library of Medicine and Science.**

Total amount of time: 37 hours

**Database:** Ovid MEDLINE(R) ALL <1946 to April 19, 2021>

**Dato for søk:** 20.04.2021
**Antall treff:** 3252

1 ((rare adj3 (disease* or disorder* or diagnos* or condition*)) or (orphan adj (disease* or disorder* or diagnosis* or condition*)) or (osteogenesis adj imperfecta) or brittle bone disease* or fragilitas ossium or osteopsathyrosis or ((lobstein* or bruck*) adj (disease* or syndrome*)) or ((skeletal or fibrous) adj3 (dysplasia* or bone*)) or achondroplas* or Diaphyseal Aclasis or ((Hereditary or Familial or Multiple) adj2 (Exostos* or Chondrodysplas* or Osteochondroma*)) or Bessel-Hagen* Disease* or (fibro* adj dysplasia*) or Jaffe Lichtenstein* or (Marfan* adj2 (syndrome* or disease* or disorder* or abiotrophy)) or (Ehlers adj Danlos) or (Loeys adj Dietz) or (genetic adj3 aortic) or ((musc* or limb-girdle) adj dystroph*) or (Glycogen Storage Disease Type adj (II or "2")) or (glycogenos* adj2 (II or "2" or generali*)) or (Pompe adj (disease* or syndrome* or disorder*)) or porphyria* or (porphyrin adj (disorder* or disease* or syndrome*)) or hemophilia* or haemophilia* or ((cystic or pancreatic) adj2 fibros*) or mucoviscidosis or mucoviscoidosis or (pancrea* adj (fibrocystic or fibros* or cystic)) or myelomeningocele* or (neural tube adj2 defect*) or spina bifida* or (congenital adj3 (limb* or extremit*)) or ((limb* or extremit*) adj3 (deformi* or malform* or anomalit*)) or Arthrogryposis multiplex congenita or Amyoplasia* or (Charcot adj Marie) or (Roussy adj Levy) or Peroneal Muscular Atroph* or Hereditary Areflexic Dystasia* or ("hereditary motor and sensory" adj neuropat*) or (HMSN adj2 (I or II or 1A or 1B or "5" or V)) or HMSN1A or HMSN1B or ((Strumpel* or Struempel*) adj2 (disease* or syndrome*)) or ((familial or hereditary) adj1 spastic adj (paralys* or parapares* or paraplegia*)) or (dejerine sottas adj (syndrome* or disease*)) or (myoton* adj2 (dystroph* or atrophica* or myopathy*)) or (Steinert* adj disease*) or Ricker Syndrome* or PROMM* or (Hereditary adj4 (Spastic Paraplegia* or spastic parapares*)) or (Spastic Paraplegia adj1 Hypertrophic Motor Sensory Neuropathy) or (hereditary adj4 neuropath*) or "CMT with Pyramidal Features" or (Spastic Paraplegia adj2 ("2" or II)) or SPG2 or (rare adj3 hereditary ataxia*) or ((DiGeorge or Di George) adj (syndrome* or sequence or anomal*)) or ((velocardiofacial or Velo Cardio Facial or 22q11* or vcf or pharyngeal pouch or Thymic Aplasia or Sedlackova or Shprintzen) adj2 syndrome*) or "Autosomal Dominant Opitz G Bbb Syndrome" or "Conotruncal Anomaly Face Syndrome" or Catch22 or neurofibromatos* or recklinghausen* or (multiple adj1 neurofibroma*) or ((Turner* or Ullrich* or XO or 45X) adj3 (syndrome* or disease* or state or status or stigma*)) or (Gonadal Dysgenesis adj XO) or Monosomy X or (Bonnevie Ullrich* adj (syndrome* or status)) or (mitochondrial adj (disease* or disorder*)) or respiratory chain deficienc* or oxidative phosphorylation deficienc* or (Noonan* adj3 (syndrome* or disease*)) or ((Klinefelter* or XXY or XXYY or XXXXY) adj3 (syndrome* or disease*)) or angelman* or happy puppet syndrome* or cfc syndrome* or cardio-facio-cutan* or cardiofaciocutan* or de lange* or amstelodamensis or (x linked adj1 cdls*) or costello* syndrome* or fcs syndrome* or faciocutaneoskeletal or facio-cutaneous-skeletal or (macrocephaly and (pseudopapilledema or lipomas) and hemangiom*) or dysplastic gangliocytoma or lhermitte duclos or hamartoma* or (ruvalcaba* adj3 syndrome*) or (bannayan* adj3 syndrome*) or (cowden* adj (disease* or syndrome*)) or (5p adj2 syndrome*) or (chromosome 5* adj4 syndrome*) or ((cri du chat or cat cry or crying cat) adj syndrome*) or ((mar* x or martin bell*) adj syndrome*) or x linked mental retardation or fragile x or frax* syndrome* or (friedreich* adj3 (ataxia* or disease*)) or hereditary spinal scleros* or ciliopath* or alstrom* syndrome* or bardet-biedl* syndrome* or caroli* disease* or ciliary motility disorder* or kartagener* syndrome* or (von hippel-lindau* adj (disease* or syndrome*)) or joubert* or kabuki* syndrome* or sarcoglycanopath* or Phelan-McDermid* or (22q13* adj3 syndrome*) or pitt-hopkin* or labhart willi* or prader willi* or royer* syndrome* or (autism dementia ataxi* adj4 syndrome*) or cerebroatrophic hyperammonemia* or (rett* adj (disorder* or syndrome*)) or 17p11* monosomy or 17p* deletion syndrome* or smith magenis* or spinal amyotroph* or ((progressive or myelopathic or spinal or spinobulbar or bulbo spinal or bulbospinal or juvenile ot infantile) adj musc* atroph*) or (peron* adj3 atroph*) or (neurogenic adj2 (amyotroph* or atroph*)) or neurogenic muscle syndrome* or bulbospinal neuronopath* or hereditary motor neuro* or (spinal adj1 bulbar muscular atroph*) or (kennedy* adj (disease* or syndrome*)) or (kugelberg welander* adj (disease* or syndrome*)) or werdnig hoffman* or chromosomal triplication or trisom* or (down* adj (syndrome* or disease*)) or ((22q13* adj3 syndrome*) and xxx) or triple x or ((william* or beuren*) adj3 syndrome*) or aortic stenosis syndrome* or hypercalcemia supravalvar aortic stenos* or epileptic syndrome* or familial epilep* or (myoclon* adj2 epilep*) or lafora* disease* or merrf* syndrome* or unverricht lundborg* or absence epilep* or frontal lobe epilep* or rolandic* epilep* or temporal lobe epilep* or landau-kleffner* or lennox gastaut* or chronic progressive hereditary chorea or huntington* or excessive somnolence or hypersomnia or kleine-levin* syndrome* or narcolepsy or cataplexy or phenylketonuria* or ((bh4 or pah or qdpr or dhpr or tetrahydrobiopterin or dihydropteridine reductase or phenylalanine hydroxylase deficiency) adj deficienc*) or folling* or foelling* or hpabh4c or hyperphenylalaninaemia* or hyperphenylalaninemia* or oligophrenia phenylpyruvica or megalencephal* or megacephal* or megalocephal* or (malformation* adj1 cort* development) or hemimegalencephal* or microcephal* or macrocephal* or tubero* scleros* or cerebral scleros* or ((bourneville* or bournaeville*) adj2 (disease* or syndrome* or phacomatosis)) or lissencephal* or agyria or microlissencephal* or Walker-Warburg* or Warburg* syndrome* or epiloia or periventricular nodular heterotopia or polymicrogyria or porencephal* or schizencephal* or cortical dysplasia* or adenoma sebaceum or agyria or double cortex or (miller* adj1 dieker*) or heterotopia* or fukuyama* or cod md syndrome* or muscular dystroph* or chemke* syndrome* or pomt1-related or hard* syndrome* or lgmd2k or mddga1 or (muscle eye brain adj (disease* or syndrome*)) or pagon* syndrome* or alpha dystroglycanopath* or micropolygyria* or polymicrogyria* or Alexander* disease* or ((musc* or myoton*) adj dystroph*) or glycogen storage disease* or lysosomal storage disease* or sialic acid storage disease or sarcoglycanopath* or myodystroph* or myopath* or myoencephalopath* or encephalomyopath* or muscle phosphofructokinase deficienc* or pfkm deficienc* or tarui* disease* or (limb-girdle adj (muscular dystroph* or syndrome*)) or duchenne* or emery dreifuss* or ((facio-scapulo-humeral or facioscapulohumeral) adj (dystroph* or atroph*)) or landouzy dejerine* or oculopharyngeal dystroph* or Ricker* syndrome* or Steinert* disease* or myotonia atrophica* or ragged red fibers or kearn-say* or kearn* syndrome* or ((oculocraniosomatic or ophthalmoplegia) adj (syndrome* or disease*)) or (ophthalmoplegia and pigmentary and degeneration and retina and cardiomyopathy) or ((melas or merff) adj syndrome*) or (mitochondr* adj (disease* or disorder*)) or fucosidosis or mucolipidoses or sphingolipidoses or fabry disease* or farber lipogranulomatosis or gangliosidoses or sandhoff* disease* or tay-sachs* disease* or gaucher* disease* or globoid cell leukodystroph* or niemann-pick* disease* or sea-blue histiocyte syndrome* or sulfatidosis or metachromatic leukodystroph* or sulfatase deficiency disease* or Pompe* disease* or acid maltase deficienc* or glucosidase deficienc* or gaa deficienc* or glycogenos* or glucogenos* or glycogen disease* or hereditary musc* disease* or giant axonal neuropath* or Refsum* disease* or familial dysautonomia* or aicardi* or agenesis of corpus callosum or callosal agenesis or chorioretinal anomalies or Dravet* or ((myoclon* or epileptic) adj encephalopath*) or (myoclon* adj2 epilep*) or doose* syndrome* or lafora* or acquired aphasia with convulsive disorder or acquired epilepti* aphasia or migrating partial epilepsy of infancy or migrating partial seizures of infancy or migrating focal seizure* or Ohtahara* or (Sturge adj2 Weber*) or ((sturge* or weber*) adj (syndrome* or disease* or krabbe)) or oculoorbital-thalamic syndrome* or hemangiomatosis syndrome* or meningo oculo facial angiomatosis or meningofacial angiomatosis or neuroretinoangiomatosis or ((GLUT or glucose transporter) adj4 syndrome*) or GLUT* deficienc* or glucose transport* defect* or De Vivo disease* or metabolic brain disease* or metabolic encephalopath* or metabolic brain disease* or dysantonomia* or dysautonomia* or autonomic dysfunction* or riley day*).tw,kf.

2 Rare Diseases/ or Osteogenesis Imperfecta/ or Exostoses, Multiple Hereditary/ or exp Fibrous Dysplasia of Bone/ or Achondroplasia/ or Marfan Syndrome/ or Ehlers-Danlos Syndrome/ or Loeys-Dietz Syndrome/ or exp Muscular Dystrophies/ or exp Glycogen Storage Disease/ or exp Porphyrias/ or Hemophilia A/ or Hemophilia B/ or Cystic Fibrosis/ or exp Neural Tube Defects/ or exp Limb Deformities, Congenital/ or Charcot-Marie-Tooth Disease/ or Spastic Paraplegia, Hereditary/ or DiGeorge Syndrome/ or exp Neurofibromatoses/ or Turner Syndrome/ or exp Mitochondrial Diseases/ or Noonan Syndrome/ or Klinefelter Syndrome/ or Angelman Syndrome/ or De Lange Syndrome/ or Costello Syndrome/ or exp Hamartoma/ or Cri-du-Chat Syndrome/ or Fragile X Syndrome/ or Friedreich Ataxia/ or Ciliopathies/ or alstrom syndrome/ or bardet-biedl syndrome/ or caroli disease/ or ciliary motility disorders/ or kartagener syndrome/ or von hippel-lindau disease/ or Prader-Willi Syndrome/ or Rett Syndrome/ or Smith-Magenis Syndrome/ or muscular atrophy, spinal/ or bulbo-spinal atrophy, x-linked/ or "spinal muscular atrophies of childhood"/ or Trisomy/ or Williams Syndrome/ or epileptic syndromes/ or epilepsies, myoclonic/ or myoclonic epilepsies, progressive/ or lafora disease/ or merrf syndrome/ or unverricht-lundborg syndrome/ or myoclonic epilepsy, juvenile/ or epilepsy, absence/ or epilepsy, frontal lobe/ or epilepsy, rolandic/ or epilepsy, temporal lobe/ or landau-kleffner syndrome/ or lennox gastaut syndrome/ or "disorders of excessive somnolence"/ or idiopathic hypersomnia/ or kleine-levin syndrome/ or narcolepsy/ or cataplexy/ or Huntington Disease/ or Phenylketonurias/ or exp "Malformations of Cortical Development"/ or down syndrome/ or trisomy 13 syndrome/ or Trisomy 18 Syndrome/ or Kearns-Sayre Syndrome/ or mitochondrial myopathies/ or mitochondrial encephalomyopathies/ or melas syndrome/ or merrf syndrome/ or exp Brain Diseases, Metabolic, Inborn/ or "hereditary sensory and autonomic neuropathies"/ or dysautonomia, familial/ or exp "hereditary sensory and motor neuropathy"/ or Aicardi Syndrome/ or Lafora Disease/ or Sturge-Weber Syndrome/

3 1 or 2

4 health literacy/ or Patient Medication Knowledge/ or attitude to health/ or health knowledge, attitudes, practice/ or Information Seeking Behavior/

5 ((health or ehealth or medic* or disease* or illness* or drug*) adj3 (literacy or literate* or numeracy or illitera* or competenc* or knowledg* or attitude* or understand* or cognition* or aware* or perception* or perceiv* or comprehend* or comprehension*)).tw,kf.

6 ((health or ehealth or medic* or disease* or illness* or drug*) adj3 information adj3 (access* or seek* or "use" or using or used or evaluat* or apply* or apprais* or obtain* or process* or need*)).tw,kf.

7 ((patient* or client* or relatives or caregiver* or carer* or next of kin* or significant other* or sibling* or spouse* or parent* or family or families) adj (know or knowing or knowledge or understand*)).tw,kf.

8 or/4-7

9 3 and 8

10 ((child* or infant*) not ((child* or infant*) and adult*)).ti.

11 (adolescent/ or exp child/ or exp infant/) not ((adolescent/ or exp child/ or exp infant/) and exp adult/)

12 10 or 11

13 9 not 12

14 limit 13 to (editorial or letter or newspaper article)

15 13 not 14

16 limit 15 to (yr="2010 -Current" and (danish or english or norwegian or swedish))

**Database**: Embase Classic+Embase <1947 to 2021 April 19>

**Dato for søk:** 20.04.2021
**Antall treff:** 4083

1 ((rare adj3 (disease* or disorder* or diagnos* or condition*)) or (orphan adj (disease* or disorder* or diagnosis* or condition*)) or (osteogenesis adj imperfecta) or brittle bone disease* or fragilitas ossium or osteopsathyrosis or ((lobstein* or bruck*) adj (disease* or syndrome*)) or ((skeletal or fibrous) adj3 (dysplasia* or bone*)) or achondroplas* or Diaphyseal Aclasis or ((Hereditary or Familial or Multiple) adj2 (Exostos* or Chondrodysplas* or Osteochondroma*)) or Bessel-Hagen* Disease* or (fibro* adj dysplasia*) or Jaffe Lichtenstein* or (Marfan* adj2 (syndrome* or disease* or disorder* or abiotrophy)) or (Ehlers adj Danlos) or (Loeys adj Dietz) or (genetic adj3 aortic) or ((musc* or limb-girdle) adj dystroph*) or (Glycogen Storage Disease Type adj (II or "2")) or (glycogenos* adj2 (II or "2" or generali*)) or (Pompe adj (disease* or syndrome* or disorder*)) or porphyria* or (porphyrin adj (disorder* or disease* or syndrome*)) or hemophilia* or haemophilia* or ((cystic or pancreatic) adj2 fibros*) or mucoviscidosis or mucoviscoidosis or (pancrea* adj (fibrocystic or fibros* or cystic)) or myelomeningocele* or (neural tube adj2 defect*) or spina bifida* or (congenital adj3 (limb* or extremit*)) or ((limb* or extremit*) adj3 (deformi* or malform* or anomalit*)) or Arthrogryposis multiplex congenita or Amyoplasia* or (Charcot adj Marie) or (Roussy adj Levy) or Peroneal Muscular Atroph* or Hereditary Areflexic Dystasia* or ("hereditary motor and sensory" adj neuropat*) or (HMSN adj2 (I or II or 1A or 1B or "5" or V)) or HMSN1A or HMSN1B or ((Strumpel* or Struempel*) adj2 (disease* or syndrome*)) or ((familial or hereditary) adj1 spastic adj (paralys* or parapares* or paraplegia*)) or (dejerine sottas adj (syndrome* or disease*)) or (myoton* adj2 (dystroph* or atrophica* or myopathy*)) or (Steinert* adj disease*) or Ricker Syndrome* or PROMM* or (Hereditary adj4 (Spastic Paraplegia* or spastic parapares*)) or (Spastic Paraplegia adj1 Hypertrophic Motor Sensory Neuropathy) or (hereditary adj4 neuropath*) or "CMT with Pyramidal Features" or (Spastic Paraplegia adj2 ("2" or II)) or SPG2 or (rare adj3 hereditary ataxia*) or ((DiGeorge or Di George) adj (syndrome* or sequence or anomal*)) or ((velocardiofacial or Velo Cardio Facial or 22q11* or vcf or pharyngeal pouch or Thymic Aplasia or Sedlackova or Shprintzen) adj2 syndrome*) or "Autosomal Dominant Opitz G Bbb Syndrome" or "Conotruncal Anomaly Face Syndrome" or Catch22 or neurofibromatos* or recklinghausen* or (multiple adj1 neurofibroma*) or ((Turner* or Ullrich* or XO or 45X) adj3 (syndrome* or disease* or state or status or stigma*)) or (Gonadal Dysgenesis adj XO) or Monosomy X or (Bonnevie Ullrich* adj (syndrome* or status)) or (mitochondrial adj (disease* or disorder*)) or respiratory chain deficienc* or oxidative phosphorylation deficienc* or (Noonan* adj3 (syndrome* or disease*)) or ((Klinefelter* or XXY or XXYY or XXXXY) adj3 (syndrome* or disease*)) or angelman* or happy puppet syndrome* or cfc syndrome* or cardio-facio-cutan* or cardiofaciocutan* or de lange* or amstelodamensis or (x linked adj1 cdls*) or costello* syndrome* or fcs syndrome* or faciocutaneoskeletal or facio-cutaneous-skeletal or (macrocephaly and (pseudopapilledema or lipomas) and hemangiom*) or dysplastic gangliocytoma or lhermitte duclos or hamartoma* or (ruvalcaba* adj3 syndrome*) or (bannayan* adj3 syndrome*) or (cowden* adj (disease* or syndrome*)) or (5p adj2 syndrome*) or (chromosome 5* adj4 syndrome*) or ((cri du chat or cat cry or crying cat) adj syndrome*) or ((mar* x or martin bell*) adj syndrome*) or x linked mental retardation or fragile x or frax* syndrome* or (friedreich* adj3 (ataxia* or disease*)) or hereditary spinal scleros* or ciliopath* or alstrom* syndrome* or bardet-biedl* syndrome* or caroli* disease* or ciliary motility disorder* or kartagener* syndrome* or (von hippel-lindau* adj (disease* or syndrome*)) or joubert* or kabuki* syndrome* or sarcoglycanopath* or Phelan-McDermid* or (22q13* adj3 syndrome*) or pitt-hopkin* or labhart willi* or prader willi* or royer* syndrome* or (autism dementia ataxi* adj4 syndrome*) or cerebroatrophic hyperammonemia* or (rett* adj (disorder* or syndrome*)) or 17p11* monosomy or 17p* deletion syndrome* or smith magenis* or spinal amyotroph* or ((progressive or myelopathic or spinal or spinobulbar or bulbo spinal or bulbospinal or juvenile ot infantile) adj musc* atroph*) or (peron* adj3 atroph*) or (neurogenic adj2 (amyotroph* or atroph*)) or neurogenic muscle syndrome* or bulbospinal neuronopath* or hereditary motor neuro* or (spinal adj1 bulbar muscular atroph*) or (kennedy* adj (disease* or syndrome*)) or (kugelberg welander* adj (disease* or syndrome*)) or werdnig hoffman* or chromosomal triplication or trisom* or (down* adj (syndrome* or disease*)) or ((22q13* adj3 syndrome*) and xxx) or triple x or ((william* or beuren*) adj3 syndrome*) or aortic stenosis syndrome* or hypercalcemia supravalvar aortic stenos* or epileptic syndrome* or familial epilep* or (myoclon* adj2 epilep*) or lafora* disease* or merrf* syndrome* or unverricht lundborg* or absence epilep* or frontal lobe epilep* or rolandic* epilep* or temporal lobe epilep* or landau-kleffner* or lennox gastaut* or chronic progressive hereditary chorea or huntington* or excessive somnolence or hypersomnia or kleine-levin* syndrome* or narcolepsy or cataplexy or phenylketonuria* or ((bh4 or pah or qdpr or dhpr or tetrahydrobiopterin or dihydropteridine reductase or phenylalanine hydroxylase deficiency) adj deficienc*) or folling* or foelling* or hpabh4c or hyperphenylalaninaemia* or hyperphenylalaninemia* or oligophrenia phenylpyruvica or megalencephal* or megacephal* or megalocephal* or (malformation* adj1 cort* development) or hemimegalencephal* or microcephal* or macrocephal* or tubero* scleros* or cerebral scleros* or ((bourneville* or bournaeville*) adj2 (disease* or syndrome* or phacomatosis)) or lissencephal* or agyria or microlissencephal* or Walker-Warburg* or Warburg* syndrome* or epiloia or periventricular nodular heterotopia or polymicrogyria or porencephal* or schizencephal* or cortical dysplasia* or adenoma sebaceum or agyria or double cortex or (miller* adj1 dieker*) or heterotopia* or fukuyama* or cod md syndrome* or muscular dystroph* or chemke* syndrome* or pomt1-related or hard* syndrome* or lgmd2k or mddga1 or (muscle eye brain adj (disease* or syndrome*)) or pagon* syndrome* or alpha dystroglycanopath* or micropolygyria* or polymicrogyria* or Alexander* disease* or ((musc* or myoton*) adj dystroph*) or glycogen storage disease* or lysosomal storage disease* or sialic acid storage disease or sarcoglycanopath* or myodystroph* or myopath* or myoencephalopath* or encephalomyopath* or muscle phosphofructokinase deficienc* or pfkm deficienc* or tarui* disease* or (limb-girdle adj (muscular dystroph* or syndrome*)) or duchenne* or emery dreifuss* or ((facio-scapulo-humeral or facioscapulohumeral) adj (dystroph* or atroph*)) or landouzy dejerine* or oculopharyngeal dystroph* or Ricker* syndrome* or Steinert* disease* or myotonia atrophica* or ragged red fibers or kearn-say* or kearn* syndrome* or ((oculocraniosomatic or ophthalmoplegia) adj (syndrome* or disease*)) or (ophthalmoplegia and pigmentary and degeneration and retina and cardiomyopathy) or ((melas or merff) adj syndrome*) or (mitochondr* adj (disease* or disorder*)) or fucosidosis or mucolipidoses or sphingolipidoses or fabry disease* or farber lipogranulomatosis or gangliosidoses or sandhoff* disease* or tay-sachs* disease* or gaucher* disease* or globoid cell leukodystroph* or niemann-pick* disease* or sea-blue histiocyte syndrome* or sulfatidosis or metachromatic leukodystroph* or sulfatase deficiency disease* or Pompe* disease* or acid maltase deficienc* or glucosidase deficienc* or gaa deficienc* or glycogenos* or glucogenos* or glycogen disease* or hereditary musc* disease* or giant axonal neuropath* or Refsum* disease* or familial dysautonomia* or riley-day* syndrome* or aicardi* or agenesis of corpus callosum or callosal agenesis or chorioretinal anomalies or Dravet* or ((myoclon* or epileptic) adj encephalopath*) or (myoclon* adj2 epilep*) or doose* syndrome* or lafora* or acquired aphasia with convulsive disorder or acquired epilepti* aphasia or migrating partial epilepsy of infancy or migrating partial seizures of infancy or migrating focal seizure* or Ohtahara* or (Sturge adj2 Weber*) or ((sturge* or weber*) adj (syndrome* or disease* or krabbe)) or oculoorbital-thalamic syndrome* or hemangiomatosis syndrome* or meningo oculo facial angiomatosis or meningofacial angiomatosis or neuroretinoangiomatosis or ((GLUT or glucose transporter) adj4 syndrome*) or GLUT* deficienc* or glucose transport* defect* or De Vivo disease* or metabolic brain disease* or metabolic encephalopath* or metabolic brain disease* or dysantonomia* or dysautonomia* or autonomic dysfunction* or riley day*).tw,kw.

2 Rare Diseases/ or Osteogenesis Imperfecta/ or Exostoses, Multiple Hereditary/ or exp Fibrous Dysplasia of Bone/ or Achondroplasia/ or Marfan Syndrome/ or Ehlers-Danlos Syndrome/ or Loeys-Dietz Syndrome/ or exp Muscular Dystrophies/ or exp Glycogen Storage Disease/ or exp Porphyrias/ or Hemophilia A/ or Hemophilia B/ or Cystic Fibrosis/ or exp Neural Tube Defects/ or exp Limb Deformities, Congenital/ or Charcot-Marie-Tooth Disease/ or Spastic Paraplegia, Hereditary/ or DiGeorge Syndrome/ or exp Neurofibromatoses/ or Turner Syndrome/ or exp Mitochondrial Diseases/ or Noonan Syndrome/ or Klinefelter Syndrome/ or happy puppet syndrome/ or de Lange syndrome/ or Costello syndrome/ or Cowden syndrome/ or multiple cancer/ or cat cry syndrome/ or fragile X syndrome/ or Friedreich ataxia/ or ciliopathy/ or alstrom syndrome/ or bardet biedl syndrome/ or caroli disease/ or joubert syndrome/ or kidney polycystic disease/ or meckel syndrome/ or nephronophthisis/ or ciliary dyskinesia/ or Kartagener syndrome/ or von Hippel Lindau disease/ or Prader Willi syndrome/ or Rett syndrome/ or Smith Magenis syndrome/ or spinal muscular atrophy/ or hereditary spinal muscular atrophy/ or kennedy disease/ or kugelberg welander disease/ or spinal muscular atrophy type 2/ or spinal muscular atrophy type 4/ or werdnig hoffmann disease/ or motor neuron disease/ or acute motor axonal neuropathy/ or amyotrophic lateral sclerosis/ or primary lateral sclerosis/ or progressive muscular atrophy/ or Kennedy disease/ or exp trisomy/ or Down syndrome/ or Williams Beuren syndrome/ or ohtahara syndrome/ or myoclonus epilepsy/ or MERRF syndrome/ or frontal lobe epilepsy/ or rolandic epilepsy/ or exp temporal lobe epilepsy/ or Landau Kleffner syndrome/ or Lennox Gastaut syndrome/ or somnolence/ or hypersomnia/ or narcolepsy/ or cataplexy/ or Huntington chorea/ or phenylketonuria/ or exp brain malformation/ or Alexander disease/ or tuberous sclerosis/ or Kearns Sayre syndrome/ or mitochondrial myopathy/ or mitochondrial encephalomyopathy/ or MELAS syndrome/ or metabolic encephalopathy/ or hereditary motor sensory neuropathy/ or dysautonomia/ or exp mental retardation malformation syndrome/ or Sturge Weber syndrome/ or exp hamartoma/

3 1 or 2

4 health literacy/ or attitude to health/ or information seeking/

5 ((health or ehealth or medic* or disease* or illness* or drug*) adj3 (literacy or literate* or numeracy or illitera* or competenc* or knowledg* or attitude* or understand* or cognition* or aware* or perception* or perceiv* or comprehend* or comprehension*)).tw,kw.

6 ((health or ehealth or medic* or disease* or illness* or drug*) adj3 information adj3 (access* or seek* or "use" or using or used or evaluat* or apply* or apprais* or obtain* or process* or need*)).tw,kw.

7 ((patient* or client* or relatives or caregiver* or carer* or next of kin* or significant other* or sibling* or spouse* or parent* or family or families) adj (know or knowing or knowledge or understand*)).tw,kw.

8 or/4-7

9 3 and 8

10 ((child* or infant*) not ((child* or infant*) and adult*)).ti.

11 (adolescent/ or exp child/ or exp infant/) not ((adolescent/ or exp child/ or exp infant/) and exp adult/)

12 10 or 11

13 9 not 12

14 limit 13 to (books or chapter or conference abstract or editorial or letter or note)

15 13 not 14

16 limit 15 to ((danish or english or norwegian or swedish) and yr="2010 -Current")

**Database:** APA PsycInfo <1806 to April Week 2 2021>
**Dato for søk:** 20.04.2021
**Antall treff:** 682

1 ((rare adj3 (disease* or disorder* or diagnos* or condition*)) or (orphan adj (disease* or disorder* or diagnosis* or condition*)) or (osteogenesis adj imperfecta) or brittle bone disease* or fragilitas ossium or osteopsathyrosis or ((lobstein* or bruck*) adj (disease* or syndrome*)) or ((skeletal or fibrous) adj3 (dysplasia* or bone*)) or achondroplas* or Diaphyseal Aclasis or ((Hereditary or Familial or Multiple) adj2 (Exostos* or Chondrodysplas* or Osteochondroma*)) or Bessel-Hagen* Disease* or (fibro* adj dysplasia*) or Jaffe Lichtenstein* or (Marfan* adj2 (syndrome* or disease* or disorder* or abiotrophy)) or (Ehlers adj Danlos) or (Loeys adj Dietz) or (genetic adj3 aortic) or ((musc* or limb-girdle) adj dystroph*) or (Glycogen Storage Disease Type adj (II or "2")) or (glycogenos* adj2 (II or "2" or generali*)) or (Pompe adj (disease* or syndrome* or disorder*)) or porphyria* or (porphyrin adj (disorder* or disease* or syndrome*)) or hemophilia* or haemophilia* or ((cystic or pancreatic) adj2 fibros*) or mucoviscidosis or mucoviscoidosis or (pancrea* adj (fibrocystic or fibros* or cystic)) or myelomeningocele* or (neural tube adj2 defect*) or spina bifida* or (congenital adj3 (limb* or extremit*)) or ((limb* or extremit*) adj3 (deformi* or malform* or anomalit*)) or Arthrogryposis multiplex congenita or Amyoplasia* or (Charcot adj Marie) or (Roussy adj Levy) or Peroneal Muscular Atroph* or Hereditary Areflexic Dystasia* or ("hereditary motor and sensory" adj neuropat*) or (HMSN adj2 (I or II or 1A or 1B or "5" or V)) or HMSN1A or HMSN1B or ((Strumpel* or Struempel*) adj2 (disease* or syndrome*)) or ((familial or hereditary) adj1 spastic adj (paralys* or parapares* or paraplegia*)) or (dejerine sottas adj (syndrome* or disease*)) or (myoton* adj2 (dystroph* or atrophica* or myopathy*)) or (Steinert* adj disease*) or Ricker Syndrome* or PROMM* or (Hereditary adj4 (Spastic Paraplegia* or spastic parapares*)) or (Spastic Paraplegia adj1 Hypertrophic Motor Sensory Neuropathy) or (hereditary adj4 neuropath*) or "CMT with Pyramidal Features" or (Spastic Paraplegia adj2 ("2" or II)) or SPG2 or (rare adj3 hereditary ataxia*) or ((DiGeorge or Di George) adj (syndrome* or sequence or anomal*)) or ((velocardiofacial or Velo Cardio Facial or 22q11* or vcf or pharyngeal pouch or Thymic Aplasia or Sedlackova or Shprintzen) adj2 syndrome*) or "Autosomal Dominant Opitz G Bbb Syndrome" or "Conotruncal Anomaly Face Syndrome" or Catch22 or neurofibromatos* or recklinghausen* or (multiple adj1 neurofibroma*) or ((Turner* or Ullrich* or XO or 45X) adj3 (syndrome* or disease* or state or status or stigma*)) or (Gonadal Dysgenesis adj XO) or Monosomy X or (Bonnevie Ullrich* adj (syndrome* or status)) or (mitochondrial adj (disease* or disorder*)) or respiratory chain deficienc* or oxidative phosphorylation deficienc* or (Noonan* adj3 (syndrome* or disease*)) or ((Klinefelter* or XXY or XXYY or XXXXY) adj3 (syndrome* or disease*)) or angelman* or happy puppet syndrome* or cfc syndrome* or cardio-facio-cutan* or cardiofaciocutan* or de lange* or amstelodamensis or (x linked adj1 cdls*) or costello* syndrome* or fcs syndrome* or faciocutaneoskeletal or facio-cutaneous-skeletal or (macrocephaly and (pseudopapilledema or lipomas) and hemangiom*) or dysplastic gangliocytoma or lhermitte duclos or hamartoma* or (ruvalcaba* adj3 syndrome*) or (bannayan* adj3 syndrome*) or (cowden* adj (disease* or syndrome*)) or (5p adj2 syndrome*) or (chromosome 5* adj4 syndrome*) or ((cri du chat or cat cry or crying cat) adj syndrome*) or ((mar* x or martin bell*) adj syndrome*) or x linked mental retardation or fragile x or frax* syndrome* or (friedreich* adj3 (ataxia* or disease*)) or hereditary spinal scleros* or ciliopath* or alstrom* syndrome* or bardet-biedl* syndrome* or caroli* disease* or ciliary motility disorder* or kartagener* syndrome* or (von hippel-lindau* adj (disease* or syndrome*)) or joubert* or kabuki* syndrome* or sarcoglycanopath* or Phelan-McDermid* or (22q13* adj3 syndrome*) or pitt-hopkin* or labhart willi* or prader willi* or royer* syndrome* or (autism dementia ataxi* adj4 syndrome*) or cerebroatrophic hyperammonemia* or (rett* adj (disorder* or syndrome*)) or 17p11* monosomy or 17p* deletion syndrome* or smith magenis* or spinal amyotroph* or ((progressive or myelopathic or spinal or spinobulbar or bulbo spinal or bulbospinal or juvenile ot infantile) adj musc* atroph*) or (peron* adj3 atroph*) or (neurogenic adj2 (amyotroph* or atroph*)) or neurogenic muscle syndrome* or bulbospinal neuronopath* or hereditary motor neuro* or (spinal adj1 bulbar muscular atroph*) or (kennedy* adj (disease* or syndrome*)) or (kugelberg welander* adj (disease* or syndrome*)) or werdnig hoffman* or chromosomal triplication or trisom* or (down* adj (syndrome* or disease*)) or ((22q13* adj3 syndrome*) and xxx) or triple x or ((william* or beuren*) adj3 syndrome*) or aortic stenosis syndrome* or hypercalcemia supravalvar aortic stenos* or epileptic syndrome* or familial epilep* or (myoclon* adj2 epilep*) or lafora* disease* or merrf* syndrome* or unverricht lundborg* or absence epilep* or frontal lobe epilep* or rolandic* epilep* or temporal lobe epilep* or landau-kleffner* or lennox gastaut* or chronic progressive hereditary chorea or huntington* or excessive somnolence or hypersomnia or kleine-levin* syndrome* or narcolepsy or cataplexy or phenylketonuria* or ((bh4 or pah or qdpr or dhpr or tetrahydrobiopterin or dihydropteridine reductase or phenylalanine hydroxylase deficiency) adj deficienc*) or folling* or foelling* or hpabh4c or hyperphenylalaninaemia* or hyperphenylalaninemia* or oligophrenia phenylpyruvica or megalencephal* or megacephal* or megalocephal* or (malformation* adj1 cort* development) or hemimegalencephal* or microcephal* or macrocephal* or tubero* scleros* or cerebral scleros* or ((bourneville* or bournaeville*) adj2 (disease* or syndrome* or phacomatosis)) or lissencephal* or agyria or microlissencephal* or Walker-Warburg* or Warburg* syndrome* or epiloia or periventricular nodular heterotopia or polymicrogyria or porencephal* or schizencephal* or cortical dysplasia* or adenoma sebaceum or agyria or double cortex or (miller* adj1 dieker*) or heterotopia* or fukuyama* or cod md syndrome* or muscular dystroph* or chemke* syndrome* or pomt1-related or hard* syndrome* or lgmd2k or mddga1 or (muscle eye brain adj (disease* or syndrome*)) or pagon* syndrome* or alpha dystroglycanopath* or micropolygyria* or polymicrogyria* or Alexander* disease* or ((musc* or myoton*) adj dystroph*) or glycogen storage disease* or lysosomal storage disease* or sialic acid storage disease or sarcoglycanopath* or myodystroph* or myopath* or myoencephalopath* or encephalomyopath* or muscle phosphofructokinase deficienc* or pfkm deficienc* or tarui* disease* or (limb-girdle adj (muscular dystroph* or syndrome*)) or duchenne* or emery dreifuss* or ((facio-scapulo-humeral or facioscapulohumeral) adj (dystroph* or atroph*)) or landouzy dejerine* or oculopharyngeal dystroph* or Ricker* syndrome* or Steinert* disease* or myotonia atrophica* or ragged red fibers or kearn-say* or kearn* syndrome* or ((oculocraniosomatic or ophthalmoplegia) adj (syndrome* or disease*)) or (ophthalmoplegia and pigmentary and degeneration and retina and cardiomyopathy) or ((melas or merff) adj syndrome*) or (mitochondr* adj (disease* or disorder*)) or fucosidosis or mucolipidoses or sphingolipidoses or fabry disease* or farber lipogranulomatosis or gangliosidoses or sandhoff* disease* or tay-sachs* disease* or gaucher* disease* or globoid cell leukodystroph* or niemann-pick* disease* or sea-blue histiocyte syndrome* or sulfatidosis or metachromatic leukodystroph* or sulfatase deficiency disease* or Pompe* disease* or acid maltase deficienc* or glucosidase deficienc* or gaa deficienc* or glycogenos* or glucogenos* or glycogen disease* or hereditary musc* disease* or giant axonal neuropath* or Refsum* disease* or familial dysautonomia* or riley-day* syndrome* or aicardi* or agenesis of corpus callosum or callosal agenesis or chorioretinal anomalies or Dravet* or ((myoclon* or epileptic) adj encephalopath*) or (myoclon* adj2 epilep*) or doose* syndrome* or lafora* or acquired aphasia with convulsive disorder or acquired epilepti* aphasia or migrating partial epilepsy of infancy or migrating partial seizures of infancy or migrating focal seizure* or Ohtahara* or (Sturge adj2 Weber*) or ((sturge* or weber*) adj (syndrome* or disease* or krabbe)) or oculoorbital-thalamic syndrome* or hemangiomatosis syndrome* or meningo oculo facial angiomatosis or meningofacial angiomatosis or neuroretinoangiomatosis or ((GLUT or glucose transporter) adj4 syndrome*) or GLUT* deficienc* or glucose transport* defect* or De Vivo disease* or metabolic brain disease* or metabolic encephalopath* or metabolic brain disease* or dysantonomia* or dysautonomia* or autonomic dysfunction* or riley day*).mp.

2 ((health or ehealth or medic* or disease* or illness* or drug*) adj3 (literacy or literate* or numeracy or illitera* or competenc* or knowledg* or attitude* or understand* or cognition* or aware* or perception* or perceiv* or comprehend* or comprehension*)).mp.

3 ((health or ehealth or medic* or disease* or illness* or drug*) adj3 information adj3 (access* or seek* or "use" or using or used or evaluat* or apply* or apprais* or obtain* or process* or need*)).mp.

4 ((patient* or client* or relatives or caregiver* or carer* or next of kin* or significant other* or sibling* or spouse* or parent* or family or families) adj (know or knowing or knowledge or understand*)).mp.

5 or/2-4

6 1 and 5

7 limit 6 to (100 childhood <birth to age 12 yrs> or 120 neonatal <birth to age 1 mo> or 140 infancy <2 to 23 mo> or 160 preschool age <age 2 to 5 yrs> or 180 school age <age 6 to 12 yrs> or 200 adolescence <age 13 to 17 yrs>)

8 limit 6 to (200 adolescence <age 13 to 17 yrs> or "300 adulthood <age 18 yrs and older>" or 320 young adulthood <age 18 to 29 yrs> or 340 thirties <age 30 to 39 yrs> or 360 middle age <age 40 to 64 yrs> or "380 aged <age 65 yrs and older>" or "390 very old <age 85 yrs and older>")

9 7 not 8

10 6 not 9

11 ((child* or infant*) not ((child* or infant*) and adult*)).ti.

12 10 not 11

13 limit 12 to ("0200 book" or "0240 authored book" or "0280 edited book" or "0300 encyclopedia")

14 12 not 13

15 limit 14 to ((danish or english or norwegian or swedish) and yr="2010 -Current")

**Database:** CINAHL (Ebsco)

**Dato for søk:** 20.04.2021

**Antall treff:** 1565

S1 ((rare N2 (disease* OR disorder* OR diagnos* OR condition*)) OR (orphan W0 (disease* OR disorder* OR diagnosis* OR condition*)) OR (osteogenesis W0 imperfecta) OR "brittle bone disease*" OR "fragilitas ossium" OR osteopsathyrosis OR ((lobstein* OR bruck*) W0 (disease* OR syndrome*)) OR ((skeletal OR fibrous) N2 (dysplasia* OR bone*)) OR achondroplas* OR "Diaphyseal Aclasis" OR ((Hereditary OR Familial OR Multiple) N1 (Exostos* OR Chondrodysplas* OR Osteochondroma*)) OR "Bessel Hagen* Disease*" OR (fibro* W0 dysplasia*) OR "Jaffe Lichtenstein*" OR (Marfan* N1 (syndrome* OR disease* OR disorder* OR abiotrophy)) OR (Ehlers W0 Danlos) OR (Loeys W0 Dietz) OR (genetic N2 aortic) OR ((musc* OR "limb girdle") W0 dystroph*) OR ("Glycogen Storage Disease Type" W0 (II OR "2")) OR (glycogenos* N1 (II OR 2 OR generali*)) OR (Pompe W0 (disease* OR syndrome* OR disorder*)) OR porphyria* OR (porphyrin W0 (disorder* OR disease* OR syndrome*)) OR hemophilia* OR haemophilia* OR ((cystic OR pancreatic) N1 fibros*) OR mucoviscidosis OR mucoviscoidosis OR (pancrea* W0 (fibrocystic OR fibros* OR cystic)) OR myelomeningocele* OR ("neural tube" N1 defect*) OR "spina bifida*" OR (congenital N2 (limb* OR extremit*)) OR ((limb* OR extremit*) N2 (deformi* OR malform* OR anomalit*)) OR "Arthrogryposis multiplex congenita" OR Amyoplasia* OR (Charcot W0 Marie) OR (Roussy W0 Levy) OR "Peroneal Muscular Atroph*" OR "Hereditary Areflexic Dystasia*" OR ("hereditary motOR and sensory" W0 neuropat*) OR (HMSN N1 (I OR II OR 1A OR 1B OR 5 OR V)) OR HMSN1A OR HMSN1B OR ((Strumpel* OR Struempel*) W0 (disease* OR syndrome)) OR ("familial spastic" W0 (paralysis OR paraparesis OR paraplegia)) OR ("dejerine sottas" W0 (syndrome* OR disease*)) OR (myoton* N1 (dystroph* OR atrophica* OR myopathy*)) OR (Steinert* W0 disease*) OR "Ricker Syndrome*" OR PROMM* OR (Hereditary N3 ("Spastic Paraplegia*" OR "spastic parapares*")) OR ("Spastic Paraplegia" W0 "Hypertrophic Motor Sensory Neuropathy") OR (hereditary N3 neuropath*) OR "CMT with Pyramidal Features" OR ("Spastic Paraplegia" N1 (2 OR II)) OR SPG2 OR (rare N2 hereditary ataxia*) OR ((DiGeorge OR "Di George") W0 (syndrome* OR sequence OR anomal*)) OR ((velocardiofacial OR "Velo Cardio Facial" OR 22q11* OR vcf OR "pharyngeal pouch" OR "Thymic Aplasia" OR Sedlackova OR Shprintzen) N1 syndrome*) OR "Autosomal Dominant Opitz G Bbb Syndrome" OR "Conotruncal Anomaly Face Syndrome" OR Catch22 OR neurofibromatos* OR recklinghausen* OR "multiple neurofibroma*" OR ((Turner* OR Ullrich* OR XO OR 45X) N2 (syndrome* OR disease* OR state OR status OR stigma*)) OR ("Gonadal Dysgenesis XO") OR Monosomy X OR ("Bonnevie Ullrich*" W0 (syndrome* OR status)) OR (mitochondrial W0 (disease* OR disorder*)) OR "respiratory chain deficienc*" OR "oxidative phosphorylation deficienc*" OR (Noonan* N2 (syndrome* OR disease*)) OR ((Klinefelter* OR XXY OR XXYY OR XXXXY) N2 (syndrome* OR disease*)) OR trisom* OR (Down* W0 (syndrome* OR disease*)) OR angelman* OR "happy puppet syndrome*" OR "cfc syndrome*" OR "cardio facio cutan*" OR cardiofaciocutan* OR "de lange*" OR amstelodamensis OR ("x linked" W0 cdls*) OR "costello* syndrome*" OR "fcs syndrome*" OR faciocutaneoskeletal OR "facio cutaneous skeletal" OR (macrocephaly and (pseudopapilledema OR lipomas) and hemangiom*) OR "dysplastic gangliocytoma" OR "lhermitte duclos" OR hamartoma OR (ruvalcaba* N2 syndrome*) OR (bannayan* N2 syndrome*) OR (cowden* W0 (disease* OR syndrome*)) OR (5p N1 syndrome*) OR (chromosome 5* N3 syndrome*) OR (("cri du chat" OR "cat cry" OR "crying cat") W0 syndrome*) OR ((mar* x OR "martin bell*") W0 syndrome*) OR "x linked mental retardation" OR "fragile x" OR "frax* syndrome*" OR (friedreich* N2 (ataxia* OR disease*)) OR "hereditary spinal scleros*" OR ciliopath* OR "alstrom* syndrome*" OR "bardet biedl* syndrome*" OR "caroli* disease*" OR "ciliary motility disorder*" OR "kartagener* syndrome*" OR ("hippel lindau* W0 (disease* OR syndrome*)) OR joubert* OR "kabuki* syndrome*" OR sarcoglycanopath* OR "Phelan McDermid*" OR (22q13* N2 syndrome*) OR "pitt hopkin*" OR "labhart willi*" OR "prader willi*" OR "royer* syndrome*" OR ("autism dementia ataxi*" N3 syndrome*) OR "cerebroatrophic hyperammonemia*" OR (rett* W0 (disorder* OR syndrome*)) OR "17p11* monosomy" OR "17p* deletion syndrome*" OR "smith magenis*" OR "spinal amyotroph*" OR ((progressive OR myelopathic OR spinal OR spinobulbar OR "bulbo spinal" OR bulbospinal OR juvenile ot infantile) W0 "muscular atroph*") OR "neurogenic scapuloperoneal amyotroph*" OR "bulbospinal neuronopath*" OR "hereditary motor neuro*" OR ("spinal bulbar muscular atroph*") OR (kennedy* W0 (disease* OR syndrome*)) OR ("kugelberg welander*" W0 (disease* OR syndrome*)) OR "werdnig hoffman" OR "chromosomal triplication" OR trisom* OR ((22q13* N2 syndrome*) and xxx) OR "triple x" OR ((william* OR beuren*) N2 syndrome*) OR "aortic stenosis syndrome*" OR "hypercalcemia supravalvar aortic stenos*" OR "epileptic syndrome*" OR "familial epilep*" OR "myoclonic epilep*" OR "lafora* disease*" OR "merrf* syndrome*" OR "unverricht lundborg* syndrome*" OR "absence epilep*" OR "frontal lobe epilep*" OR "rolandic* epilep*" OR "temporal lobe epilep*" OR "landau kleffner* syndrome*" OR "lennox gastaut* syndrome*" OR "chronic progressive hereditary chorea" OR huntington* OR "excessive somnolence" OR hypersomnia OR "kleine levin* syndrome*" OR narcolepsy OR cataplexy OR phenylketonuria* OR ((bh4 OR pah OR qdpr OR dhpr OR tetrahydrobiopterin OR "dihydropteridine reductase" OR "phenylalanine hydroxylase deficiency") W0 deficienc*) OR folling* OR foelling* OR hpabh4c OR hyperphenylalaninaemia* OR hyperphenylalaninemia* OR oligophrenia phenylpyruvica OR megalencephal* OR megacephal* OR megalocephal* OR "cort* development malformation*" OR hemimegalencephal* OR microcephal* OR macrocephal* OR "tubero* scleros*" OR "cerebral scleros*" OR ((bourneville* OR bournaeville*) N1 (disease* OR syndrome* OR phacomatosis)) OR lissencephal* OR agyria OR microlissencephal* OR "Walker Warburg*" OR "Warburg* syndrome*" OR epiloia OR "periventricular nodular heterotopia" OR polymicrogyria OR porencephal* OR schizencephal* OR cortical dysplasia* OR adenoma sebaceum OR agyria OR double cortex OR "miller* dieker*" OR "dieker* miller*" OR heterotopia* OR fukuyama* OR "cod md syndrome*" OR "muscular dystroph*" OR "chemke* syndrome*" OR "pomt1 related" OR "hard* syndrome*" OR lgmd2k OR mddga1 OR ("muscle eye brain" W0 (disease* OR syndrome*)) OR "pagon* syndrome*" OR "alpha dystroglycanopath*" OR micropolygyria* OR polymicrogyria* OR "Alexander* disease*" OR ((musc* or myoton*) W0 dystroph*) OR "glycogen storage disease*" OR "lysosomal storage disease*" OR "sialic acid storage disease*" OR sarcoglycanopath* OR myodystroph* OR myopath* OR myoencephalopath* OR encephalomyopath* OR "muscle phosphofructokinase deficienc*" OR "pfkm deficienc*" OR "tarui* disease*" OR (limb-girdle W0 (muscular dystroph* or syndrome*)) OR "duchenne* OR "emery dreifuss*" OR ((facio-scapulo-humeral or facioscapulohumeral) W0 (dystroph* or atroph*)) or "landouzy dejerine*" OR "oculopharyngeal dystroph*" OR "Ricker* syndrome*" OR "Steinert* disease*" OR "myotonia atrophica*" OR "ragged red fibers" OR "kearn-say*" OR "kearn* syndrome*" OR ((oculocraniosomatic OR ophthalmoplegia) W0 (syndrome* or disease*)) OR (ophthalmoplegia and pigmentary and degeneration and retina and cardiomyopathy) OR ((melas OR merff) W0 syndrome*) OR (mitochondr* W0 (disease* or disorder*)) OR fucosidosis OR mucolipidoses OR sphingolipidoses OR "fabry disease*" OR "farber lipogranulomatosis" OR gangliosidoses OR "sandhoff* disease*" or "tay-sachs* disease*" or "gaucher* disease*" OR "globoid cell leukodystroph*" OR "niemann-pick* disease*" OR "sea-blue histiocyte syndrome*" OR sulfatidosis OR "metachromatic leukodystroph*" OR "sulfatase deficiency disease*" OR "Pompe* disease*" OR "acid maltase deficienc*" OR "glucosidase deficienc*" OR "gaa deficienc*" OR glycogenos* OR glucogenos* OR "glycogen disease*" OR "hereditary musc* disease*" OR "giant axonal neuropath*" OR "Refsum* disease*" OR "familial dysautonomia*" OR aicardi* OR "agenesis of corpus callosum" OR "callosal agenesis" OR "chorioretinal anomalies" OR Dravet* OR ((myoclon* OR epileptic) W0 encephalopath*) OR (myoclon* N1 epilep*) OR "doose* syndrome*" OR lafora* OR "acquired aphasia with convulsive disorder" OR "acquired epilepti* aphasia" OR "migrating partial epilepsy of infancy" OR "migrating partial seizures of infancy" OR "migrating focal seizure*" OR Ohtahara* OR (Sturge N1 Weber*) OR ((sturge* OR weber*) W0 (syndrome* OR disease* OR krabbe)) OR "oculoorbital-thalamic syndrome*" OR "hemangiomatosis syndrome*" OR "meningo oculo facial angiomatosis" OR "meningofacial angiomatosis" OR neuroretinoangiomatosis OR ((GLUT OR "glucose transporter") N3 syndrome*) OR "GLUT* deficienc*" OR "glucose transport* defect*" OR "De Vivo disease*" or "metabolic brain disease*" OR "metabolic encephalopath*" or "metabolic brain disease*" OR dysantonomia* or dysautonomia* OR "autonomic dysfunction*" OR "riley day*")

S2 (MH "Rare Diseases") OR (MH "Osteogenesis Imperfecta") OR (MH "Fibrous Dysplasia of Bone+") OR (MH "Achondroplasia") OR (MH "Marfan Syndrome") OR (MH "Ehlers-Danlos Syndrome") OR (MH "Loeys-Dietz Syndrome") OR (MH "Muscular Dystrophy+") OR (MH "Glycogen Storage Disease") OR (MH "Glucose Transporter 1 Deficiency Syndrome") OR (MH "Porphyrias+") OR (MH "Hemophilia+") OR (MH "Cystic Fibrosis") OR (MH "Neural Tube Defects+") OR (MH "Limb Deformities, Congenital+") OR (MH "Alstrom Syndrome") OR (MH "Charcot-Marie-Tooth Disease") OR (MH "Spastic Paraplegia, Hereditary") OR (MH "Neuropathies, Hereditary Motor and Sensory+") OR (MH "DiGeorge Syndrome") OR (MH "22q11 Deletion Syndrome+") OR (MH "Neurofibromatoses+") OR (MH "Turner's Syndrome") OR (MH "Mitochondrial Diseases+") OR (MH "Noonan Syndrome") OR (MH "Klinefelter's Syndrome") OR (MH "Angelman Syndrome") OR (MH "De Lange Syndrome") OR (MH "Costello Syndrome") OR (MH "Hamartoma Syndrome, Multiple+") OR (MH "Cri-Du-Chat Syndrome") OR (MH "Fragile X Syndrome") OR (MH "Friedreich's Ataxia") OR (MH "Ciliopathies") OR (MH "Bardet-Biedl Syndrome") OR (MH "Ciliary Motility Disorders") OR (MH "von Hippel-Lindau Disease") OR (MH "Prader-Willi Syndrome") OR (MH "Rett Syndrome") OR (MH "Smith-Magenis Syndrome") OR (MH "Muscular Atrophy, Spinal") OR (MH "Bulbo-Spinal Atrophy, X-Linked") OR (MH "Trisomy 13") OR (MH "Trisomy 18") OR (MH "Down Syndrome") OR (MH "Williams Syndrome") OR (MH "Epilepsies, Myoclonic+") OR (MH "Epilepsy, Absence") OR (MH "Epilepsy, Rolandic") OR (MH "Epilepsy, Temporal Lobe") OR (MH "Epilepsy, Partial") OR (MH "Epilepsy, Partial, Complex") OR (MH "Epilepsy, Partial, Focal") OR (MH "Landau-Kleffner Syndrome") OR (MH "Lennox-Gastaut Syndrome") OR (MH "Disorders of Excessive Somnolence") OR (MH "Kleine-Levin Syndrome") OR (MH "Narcolepsy") OR (MH "Huntington's Disease") OR (MH "Phenylketonuria") OR (MH "Malformations of Cortical Development") OR (MH "Lissencephaly") OR (MH "Brain Diseases, Metabolic, Inborn+") OR (MH "Aicardi Syndrome") OR (MH "Sturge-Weber Syndrome") OR (MH "Intellectual Disability+")

S3 S1 OR S2

S4 (MH "Information Literacy") OR (MH "Health Literacy") OR (MH "Attitude to Health") OR (MH "Information Seeking Behavior")

S5 ((health OR ehealth OR medic* OR disease* OR illness* OR drug*) N2 (literacy OR literate* OR numeracy OR illitera* OR competenc* OR knowledg* OR attitude* OR understand* OR cognition* OR aware* OR perception* OR perceiv* OR comprehend* OR comprehension*))

S6 ((health OR ehealth OR medic* OR disease* OR illness* OR drug*) N2 information N2 (access* OR seek* OR "use" OR using OR used OR evaluat* OR apply* OR apprais* OR obtain* OR process* OR need*))

S7 ((patient* OR client* OR relatives OR caregiver* OR carer* OR "next of kin*" OR significant other* OR sibling* OR spouse* OR parent* OR family OR families) W0 (know OR knowing OR knowledge OR understand*))

S8 S4 OR S5 OR S6 OR S7

S9 S3 AND S8 Limiters - Age Groups: Adult: 19-44 years, Middle Aged: 45-64 years, Aged: 65+ years, Aged, 80 and over

S10 S3 AND S8 Limiters - Age Groups: Infant, Newborn: birth-1 month, Infant: 1-23 months, Child, Preschool: 2-5 years, Child: 6-12 years, Adolescent: 13-18 years

S11 S10 NOT S9

S12 TI ((child* OR infant*) NOT ((child* OR infant*) AND adult*))

S13 S3 AND S8

S14 S13 NOT (S11 OR S12) Limiters - Published Date: 20100101-20210431; Language: Danish, English, Norwegian, Swedish

S15 S3 AND S8 Limiters - Publication Type: Anecdote, Bibliography, Biography, Book, Book Chapter, Book Review, Brief Item, Care Plan, Cartoon, Commentary, Diagnostic Images, Editorial, Games, Glossary, Individual Testimonial Website, Letter, Protocol, Website

S16 S14 NOT S15

**Database:** ERIC <1965 to January 2021>

**Dato for søk:** 20.04.2021

**Antall treff:** 22

1 ((rare adj3 (disease* or disorder* or diagnos* or condition*)) or (orphan adj (disease* or disorder* or diagnosis* or condition*)) or (osteogenesis adj imperfecta) or brittle bone disease* or fragilitas ossium or osteopsathyrosis or ((lobstein* or bruck*) adj (disease* or syndrome*)) or ((skeletal or fibrous) adj3 (dysplasia* or bone*)) or achondroplas* or Diaphyseal Aclasis or ((Hereditary or Familial or Multiple) adj2 (Exostos* or Chondrodysplas* or Osteochondroma*)) or Bessel-Hagen* Disease* or (fibro* adj dysplasia*) or Jaffe Lichtenstein* or (Marfan* adj2 (syndrome* or disease* or disorder* or abiotrophy)) or (Ehlers adj Danlos) or (Loeys adj Dietz) or (genetic adj3 aortic) or ((musc* or limb-girdle) adj dystroph*) or (Glycogen Storage Disease Type adj (II or "2")) or (glycogenos* adj2 (II or "2" or generali*)) or (Pompe adj (disease* or syndrome* or disorder*)) or porphyria* or (porphyrin adj (disorder* or disease* or syndrome*)) or hemophilia* or haemophilia* or ((cystic or pancreatic) adj2 fibros*) or mucoviscidosis or mucoviscoidosis or (pancrea* adj (fibrocystic or fibros* or cystic)) or myelomeningocele* or (neural tube adj2 defect*) or spina bifida* or (congenital adj3 (limb* or extremit*)) or ((limb* or extremit*) adj3 (deformi* or malform* or anomalit*)) or Arthrogryposis multiplex congenita or Amyoplasia* or (Charcot adj Marie) or (Roussy adj Levy) or Peroneal Muscular Atroph* or Hereditary Areflexic Dystasia* or ("hereditary motor and sensory" adj neuropat*) or (HMSN adj2 (I or II or 1A or 1B or "5" or V)) or HMSN1A or HMSN1B or ((Strumpel* or Struempel*) adj2 (disease* or syndrome*)) or ((familial or hereditary) adj1 spastic adj (paralys* or parapares* or paraplegia*)) or (dejerine sottas adj (syndrome* or disease*)) or (myoton* adj2 (dystroph* or atrophica* or myopathy*)) or (Steinert* adj disease*) or Ricker Syndrome* or PROMM* or (Hereditary adj4 (Spastic Paraplegia* or spastic parapares*)) or (Spastic Paraplegia adj1 Hypertrophic Motor Sensory Neuropathy) or (hereditary adj4 neuropath*) or "CMT with Pyramidal Features" or (Spastic Paraplegia adj2 ("2" or II)) or SPG2 or (rare adj3 hereditary ataxia*) or ((DiGeorge or Di George) adj (syndrome* or sequence or anomal*)) or ((velocardiofacial or Velo Cardio Facial or 22q11* or vcf or pharyngeal pouch or Thymic Aplasia or Sedlackova or Shprintzen) adj2 syndrome*) or "Autosomal Dominant Opitz G Bbb Syndrome" or "Conotruncal Anomaly Face Syndrome" or Catch22 or neurofibromatos* or recklinghausen* or (multiple adj1 neurofibroma*) or ((Turner* or Ullrich* or XO or 45X) adj3 (syndrome* or disease* or state or status or stigma*)) or (Gonadal Dysgenesis adj XO) or Monosomy X or (Bonnevie Ullrich* adj (syndrome* or status)) or (mitochondrial adj (disease* or disorder*)) or respiratory chain deficienc* or oxidative phosphorylation deficienc* or (Noonan* adj3 (syndrome* or disease*)) or ((Klinefelter* or XXY or XXYY or XXXXY) adj3 (syndrome* or disease*)) or angelman* or happy puppet syndrome* or cfc syndrome* or cardio-facio-cutan* or cardiofaciocutan* or de lange* or amstelodamensis or (x linked adj1 cdls*) or costello* syndrome* or fcs syndrome* or faciocutaneoskeletal or facio-cutaneous-skeletal or (macrocephaly and (pseudopapilledema or lipomas) and hemangiom*) or dysplastic gangliocytoma or lhermitte duclos or hamartoma* or (ruvalcaba* adj3 syndrome*) or (bannayan* adj3 syndrome*) or (cowden* adj (disease* or syndrome*)) or (5p adj2 syndrome*) or (chromosome 5* adj4 syndrome*) or ((cri du chat or cat cry or crying cat) adj syndrome*) or ((mar* x or martin bell*) adj syndrome*) or x linked mental retardation or fragile x or frax* syndrome* or (friedreich* adj3 (ataxia* or disease*)) or hereditary spinal scleros* or ciliopath* or alstrom* syndrome* or bardet-biedl* syndrome* or caroli* disease* or ciliary motility disorder* or kartagener* syndrome* or (von hippel-lindau* adj (disease* or syndrome*)) or joubert* or kabuki* syndrome* or sarcoglycanopath* or Phelan-McDermid* or (22q13* adj3 syndrome*) or pitt-hopkin* or labhart willi* or prader willi* or royer* syndrome* or (autism dementia ataxi* adj4 syndrome*) or cerebroatrophic hyperammonemia* or (rett* adj (disorder* or syndrome*)) or 17p11* monosomy or 17p* deletion syndrome* or smith magenis* or spinal amyotroph* or ((progressive or myelopathic or spinal or spinobulbar or bulbo spinal or bulbospinal or juvenile ot infantile) adj musc* atroph*) or (peron* adj3 atroph*) or (neurogenic adj2 (amyotroph* or atroph*)) or neurogenic muscle syndrome* or bulbospinal neuronopath* or hereditary motor neuro* or (spinal adj1 bulbar muscular atroph*) or (kennedy* adj (disease* or syndrome*)) or (kugelberg welander* adj (disease* or syndrome*)) or werdnig hoffman* or chromosomal triplication or trisom* or (down* adj (syndrome* or disease*)) or ((22q13* adj3 syndrome*) and xxx) or triple x or ((william* or beuren*) adj3 syndrome*) or aortic stenosis syndrome* or hypercalcemia supravalvar aortic stenos* or epileptic syndrome* or familial epilep* or (myoclon* adj2 epilep*) or lafora* disease* or merrf* syndrome* or unverricht lundborg* or absence epilep* or frontal lobe epilep* or rolandic* epilep* or temporal lobe epilep* or landau-kleffner* or lennox gastaut* or chronic progressive hereditary chorea or huntington* or excessive somnolence or hypersomnia or kleine-levin* syndrome* or narcolepsy or cataplexy or phenylketonuria* or ((bh4 or pah or qdpr or dhpr or tetrahydrobiopterin or dihydropteridine reductase or phenylalanine hydroxylase deficiency) adj deficienc*) or folling* or foelling* or hpabh4c or hyperphenylalaninaemia* or hyperphenylalaninemia* or oligophrenia phenylpyruvica or megalencephal* or megacephal* or megalocephal* or (malformation* adj1 cort* development) or hemimegalencephal* or microcephal* or macrocephal* or tubero* scleros* or cerebral scleros* or ((bourneville* or bournaeville*) adj2 (disease* or syndrome* or phacomatosis)) or lissencephal* or agyria or microlissencephal* or Walker-Warburg* or Warburg* syndrome* or epiloia or periventricular nodular heterotopia or polymicrogyria or porencephal* or schizencephal* or cortical dysplasia* or adenoma sebaceum or agyria or double cortex or (miller* adj1 dieker*) or heterotopia* or fukuyama* or cod md syndrome* or muscular dystroph* or chemke* syndrome* or pomt1-related or hard* syndrome* or lgmd2k or mddga1 or (muscle eye brain adj (disease* or syndrome*)) or pagon* syndrome* or alpha dystroglycanopath* or micropolygyria* or polymicrogyria* or Alexander* disease* or ((musc* or myoton*) adj dystroph*) or glycogen storage disease* or lysosomal storage disease* or sialic acid storage disease or sarcoglycanopath* or myodystroph* or myopath* or myoencephalopath* or encephalomyopath* or muscle phosphofructokinase deficienc* or pfkm deficienc* or tarui* disease* or (limb-girdle adj (muscular dystroph* or syndrome*)) or duchenne* or emery dreifuss* or ((facio-scapulo-humeral or facioscapulohumeral) adj (dystroph* or atroph*)) or landouzy dejerine* or oculopharyngeal dystroph* or Ricker* syndrome* or Steinert* disease* or myotonia atrophica* or ragged red fibers or kearn-say* or kearn* syndrome* or ((oculocraniosomatic or ophthalmoplegia) adj (syndrome* or disease*)) or (ophthalmoplegia and pigmentary and degeneration and retina and cardiomyopathy) or ((melas or merff) adj syndrome*) or (mitochondr* adj (disease* or disorder*)) or fucosidosis or mucolipidoses or sphingolipidoses or fabry disease* or farber lipogranulomatosis or gangliosidoses or sandhoff* disease* or tay-sachs* disease* or gaucher* disease* or globoid cell leukodystroph* or niemann-pick* disease* or sea-blue histiocyte syndrome* or sulfatidosis or metachromatic leukodystroph* or sulfatase deficiency disease* or Pompe* disease* or acid maltase deficienc* or glucosidase deficienc* or gaa deficienc* or glycogenos* or glucogenos* or glycogen disease* or hereditary musc* disease* or giant axonal neuropath* or Refsum* disease* or familial dysautonomia* or riley-day* syndrome* or aicardi* or agenesis of corpus callosum or callosal agenesis or chorioretinal anomalies or Dravet* or ((myoclon* or epileptic) adj encephalopath*) or (myoclon* adj2 epilep*) or doose* syndrome* or lafora* or acquired aphasia with convulsive disorder or acquired epilepti* aphasia or migrating partial epilepsy of infancy or migrating partial seizures of infancy or migrating focal seizure* or Ohtahara* or (Sturge adj2 Weber*) or ((sturge* or weber*) adj (syndrome* or disease* or krabbe)) or oculoorbital-thalamic syndrome* or hemangiomatosis syndrome* or meningo oculo facial angiomatosis or meningofacial angiomatosis or neuroretinoangiomatosis or ((GLUT or glucose transporter) adj4 syndrome*) or GLUT* deficienc* or glucose transport* defect* or De Vivo disease* or metabolic brain disease* or metabolic encephalopath* or metabolic brain disease* or dysantonomia* or dysautonomia* or autonomic dysfunction* or riley day*).mp.

2 ((health or ehealth or medic* or disease* or illness* or drug*) adj3 (literacy or literate* or numeracy or illitera* or competenc* or knowledg* or attitude* or understand* or cognition* or aware* or perception* or perceiv* or comprehend* or comprehension*)).mp.

3 ((health or ehealth or medic* or disease* or illness* or drug*) adj3 information adj3 (access* or seek* or "use" or using or used or evaluat* or apply* or apprais* or obtain* or process* or need*)).mp.

4 ((patient* or client* or relatives or caregiver* or carer* or next of kin* or significant other* or sibling* or spouse* or parent* or family or families) adj (know or knowing or knowledge or understand*)).mp.

5 or/2-4

6 1 and 5

7 limit 6 to ((danish or english or norwegian or swedish) and yr="2010 -Current")
