# Supplementary material for: A scoping review of health literacy in rare disorders: key issues and research directions
Source: Orphanet J Rare Dis. 2024 Sep 6;19:328. doi: 10.1186/s13023-024-03332-5 (PMC11380335; doi:10.1186/s13023-024-03332-5)
Supplement: Supplementary file 4 — Supplementary Material 4 [file 13023_2024_3332_MOESM4_ESM.docx]

Additional file 4.

Table 1. Included diagnoses.

| **Diagnosis** | **ORPHA** | **ICD-10** | **Number of reports** | **Number of patients** | **Number of carers** | **Author** |
| --- | --- | --- | --- | --- | --- | --- |
| Autoimmune Addison | 85138 | E27.1 | 1 | 5 |  | Mälstam 2018 [[1]](https://paperpile.com/c/iSaHpS/uP8In) |
| Charcot Marie Tooth disease | 166 | G60.0 | 1 | 24 |  | Socha Hernandez 2020 [[2]](https://paperpile.com/c/iSaHpS/Gj58s) |
| Churg-Strauss syndrome (Eosinophilic granulomatosis with polyangiitis) | 183 | M30.1 | 2 |  |  | Carpenter 2011 [[3]](https://paperpile.com/c/iSaHpS/jRwSA) |
|  |  |  |  | 29 |  |  |
|  |  |  |  | 3 |  | Mooney 2013 [[4]](https://paperpile.com/c/iSaHpS/EG7M1) |
| Congenital dysfibrinogenemia | 98891 | D68.2 | 1 | 1 |  | Arya 2021 [[5]](https://paperpile.com/c/iSaHpS/t1A48) |
| Congenital hypogonadotropic hypogonadism | 174590 | E23.0 | 1 | 105 |  | Dwyer 2014 [[6]](https://paperpile.com/c/iSaHpS/edkmx) |
| Cystic Fibrosis | 586 | E84.0, E84.1, E84.8, E84.9 | 14 | 49 | 50 | Beinke 2017 [[7]](https://paperpile.com/c/iSaHpS/jj0Rb) |
|  |  |  |  | 91 |  | Chaudry 2013 [[8]](https://paperpile.com/c/iSaHpS/nFDQh) |
|  |  |  |  | 42 |  | David 2015 [[9]](https://paperpile.com/c/iSaHpS/8AfYl) |
|  |  |  |  | 10 | 10 | Dellon 2018 [[10]](https://paperpile.com/c/iSaHpS/EKEYE) |
|  |  |  |  | 250 |  | Flewelling 2019 [[11]](https://paperpile.com/c/iSaHpS/beAHp) |
|  |  |  |  |  | 241 | Ioannou 2010 [[12]](https://paperpile.com/c/iSaHpS/XnKyc) |
|  |  |  |  | 251 |  | Jackson 2019 [[13]](https://paperpile.com/c/iSaHpS/jsDY3) |
|  |  |  |  | 22 |  | Kazmerski 2017 [[14]](https://paperpile.com/c/iSaHpS/ohqhm) |
|  |  |  |  | 24 |  | Keyte 2020 [[15]](https://paperpile.com/c/iSaHpS/FFJnW) |
|  |  |  |  | 97 | 182 | Kirk 2016 [[16]](https://paperpile.com/c/iSaHpS/YmSnm) |
|  |  |  |  | 45 |  | Lewis 2016 [[17]](https://paperpile.com/c/iSaHpS/3t4dl) |
|  |  |  |  | 78 | 65 | Lonabaugh 2018 [[18]](https://paperpile.com/c/iSaHpS/i4BJ8) |
|  |  |  |  | 42 |  | Pakhale 2016 [[19]](https://paperpile.com/c/iSaHpS/ywma5) |
|  |  |  |  | 39 |  | Sylvain 2016 [[20]](https://paperpile.com/c/iSaHpS/Eg2D9) |
| Diffuse cutaneous systemic sclerosis | 220393 | M34.0 | 1 | 10 |  | Gumuchian 2018 [[21]](https://paperpile.com/c/iSaHpS/Yj9yv) |
| Duchenne muscular dystrophy | 98896 | G71.0 | 1 | 9 | 9 | Hiermeier 2020 [[22]](https://paperpile.com/c/iSaHpS/P1xsD) |
| Epidermolysis bullosa | 303 | Q81.2 | 1 | 33 |  | Parvizi 2017 [[23]](https://paperpile.com/c/iSaHpS/jjcOp) |
| Erythropietic protoporphyria | 79270 | E80.0 | 1 | 10 | 14 | Naik 2019 [[24]](https://paperpile.com/c/iSaHpS/CX99b) |
| Familial amyloid polyneuropathy | 85447 | E85.1 | 1 | 8 | 2 | Theaudin 2014 [[25]](https://paperpile.com/c/iSaHpS/9gH2j) |
| Familial chylomicronemia syndrome | 444490 | E78.3 | 1 | 50 |  | Salvatore 2018 [[26]](https://paperpile.com/c/iSaHpS/meKVh) |
| Fragile X syndrome (symptomatic) | 449291 | Q99 | 1 | 142 |  | Smolich 2020 [[27]](https://paperpile.com/c/iSaHpS/DrqIz) |
| FXTAS syndrome carriers | 93256 | G11.2 | 1 | 10 |  | Walsh 2021 [[28]](https://paperpile.com/c/iSaHpS/0cDXL) |
| Glanzmanns thrombasthenia | 849 | D69.1 | 1 | 1 |  | Arya 2021 [[5]](https://paperpile.com/c/iSaHpS/t1A48) |
| Guillain-Barre syndrome | 2103 | G61.0 | 1 | 16 |  | Akanuwe 2020 [[29]](https://paperpile.com/c/iSaHpS/RUCcm) |
| Hemophilia | 448, 169808, 1690805, 169802, 169796, 169799, 169793 | D66, D67 | 5 | 4 |  | Kesselheim 2015 [[30]](https://paperpile.com/c/iSaHpS/pdfyS) |
|  |  |  |  | 17 |  | Le Dore 2021 [[31]](https://paperpile.com/c/iSaHpS/pSCR8) |
|  |  |  |  | 39 |  | Mohan 2021 [[32]](https://paperpile.com/c/iSaHpS/TlASj) |
|  |  |  |  | 23 |  | Torres-Ortuno 2018 [[33]](https://paperpile.com/c/iSaHpS/R5a1B) |
|  |  |  |  | 413 |  | Lindvall 2010 [[34]](https://paperpile.com/c/iSaHpS/PeeTo) |
| Hemophilia type A | 98878 | D66 | 5 | 78 |  | Arnold 2014 [[35]](https://paperpile.com/c/iSaHpS/oNVM2) |
|  |  |  |  | 54 |  | De la Corte-Rodriguez 2019 [[36]](https://paperpile.com/c/iSaHpS/63lsY) |
|  |  |  |  | 130 |  | Mohan 2021 [[32]](https://paperpile.com/c/iSaHpS/TlASj) |
|  |  |  |  | 23 |  | Mulders 2012 [[37]](https://paperpile.com/c/iSaHpS/CWJcI) |
|  |  |  |  | 12 |  | Van-Balen 2019 [[38]](https://paperpile.com/c/iSaHpS/fm2Kq) |
| Hemophilia type B | 98879 | D67 | 4 | 26 |  | Arnold 2014 [[35]](https://paperpile.com/c/iSaHpS/oNVM2) |
|  |  |  |  | 12 |  | De la Corte-Rodriguez 2019 [[36]](https://paperpile.com/c/iSaHpS/63lsY) |
|  |  |  |  | 7 |  | Mulders 2012 [[37]](https://paperpile.com/c/iSaHpS/CWJcI) |
|  |  |  |  | 6 |  | Van-Balen 2019 [[38]](https://paperpile.com/c/iSaHpS/fm2Kq) |
| Mild hemophilia A | 169808 | D66 | 1 | 8 |  | Bhatt 2021 [[39]](https://paperpile.com/c/iSaHpS/UVMfW) |
| Moderate hemophilia A | 169805 | D66 | 1 | 4 |  | Bhatt 2021 [[39]](https://paperpile.com/c/iSaHpS/UVMfW) |
| Severe hemophilia A | 169802 | D66 | 3 | 21 |  | Bhatt 2021 [[39]](https://paperpile.com/c/iSaHpS/UVMfW) |
|  |  |  |  | 11 |  | Hoefnagels 2020 [[40]](https://paperpile.com/c/iSaHpS/GHYFY) |
|  |  |  |  | 75 |  | Le Dore 2021 [[31]](https://paperpile.com/c/iSaHpS/pSCR8) |
| Mild hemophilia B | 169799 | D67 | 1 | 1 |  | Bhatt 2021 [[39]](https://paperpile.com/c/iSaHpS/UVMfW) |
| Moderate hemophilia B | 169796 | D67 | 1 | 2 |  | Bhatt 2021 [[39]](https://paperpile.com/c/iSaHpS/UVMfW) |
| Severe hemophilia B | 169793 | D67 | 1 | 5 |  | Bhatt 2021 [[39]](https://paperpile.com/c/iSaHpS/UVMfW) |
| Symptomatic hemophilia A carrier | 177926 | D66 | 1 | 5 |  | Arya 2021 [[5]](https://paperpile.com/c/iSaHpS/t1A48) |
| Symptomatic hemophilia B carrier | 177929 | D67 | 1 | 2 |  | Arya 2021 [[5]](https://paperpile.com/c/iSaHpS/t1A48) |
| Hermansky-Pudlak syndrome | 79430 | E70.3 | 1 | 1 |  | Arya 2021 [[5]](https://paperpile.com/c/iSaHpS/t1A48) |
| Huntington disease | 399 | G10.0 | 7 | 87 |  | Arran 2014 [[41]](https://paperpile.com/c/iSaHpS/Fu5nj) |
|  |  |  |  |  | 683 | Braisch 2016 [[42]](https://paperpile.com/c/iSaHpS/R9hJi) |
|  |  |  |  |  | 55 | Domaradzki 2016 [[43]](https://paperpile.com/c/iSaHpS/VICQF) |
|  |  |  |  | 15 | 9 | Etchegary 2011 [[44]](https://paperpile.com/c/iSaHpS/H1QKo) |
|  |  |  |  | 20 |  | Ringqvist 2021 [[45]](https://paperpile.com/c/iSaHpS/XlHCb) |
|  |  |  |  |  | 227 | Skirton 2010 [[46]](https://paperpile.com/c/iSaHpS/fOyps) |
|  |  |  |  |  | 58 | Smedley 2021 [[47]](https://paperpile.com/c/iSaHpS/8OAv1) |
| Hypermobile Ehler-Danlos syndrome | 285 | Q79.6 | 1 | 19 | 9 | Chaleat-Valayer 2019 [[48]](https://paperpile.com/c/iSaHpS/552OA) |
| Idiopathic basal ganglia calcification 3 | 1980 | G23.8 | 1 | 6 |  | Takeuchi 2016 [[49]](https://paperpile.com/c/iSaHpS/xZeI8) |
| Limited systemic sclerosis | 220407 | M34.0 | 1 | 7 |  | Gumuchian 2018 [[21]](https://paperpile.com/c/iSaHpS/Yj9yv) |
| Marfan syndrome | 558 | Q87.4 | 1 | 11 |  | Depping 2021 [[50]](https://paperpile.com/c/iSaHpS/yLaEa) |
| Microscopic polyangiitis | 727 | M31.7 | 2 | 18 |  | Carpenter 2011 [[3]](https://paperpile.com/c/iSaHpS/jRwSA) |
|  |  |  |  | 1 |  | Mooney 2013 [[4]](https://paperpile.com/c/iSaHpS/EG7M1) |
| Moebius syndrome | 570 | Q87.0 | 1 | 50 | 57 | Bogart 2017 [[51]](https://paperpile.com/c/iSaHpS/liMJM) |
| Monosomy 22q13.3 | 48652 | Q93.5 | 1 | 5 |  | Kesselheim 2015 [[30]](https://paperpile.com/c/iSaHpS/pdfyS) |
| Motor neuron disease | 98503 | G12.2 | 1 | 34 |  | Foley 2014 [[52]](https://paperpile.com/c/iSaHpS/1hHHE) |
| Myasthenia gravis | 589 | G70.0 | 2 | 51 |  | Katavic 2016 [[53]](https://paperpile.com/c/iSaHpS/MLdJN) |
|  |  |  |  | 5 |  | Katavic 2019 [[54]](https://paperpile.com/c/iSaHpS/E1bfM) |
| Myelomeningocele | 93969 | Q05.0-Q05.9 | 1 | 35 |  | Dicianno 2016 [[55]](https://paperpile.com/c/iSaHpS/8yWpK) |
| Myotonic dystrophy type 1 | 273 | G71.1 | 3 | 40 |  | Coathup 2016 [[56]](https://paperpile.com/c/iSaHpS/0HQIP) |
|  |  |  |  | 200 |  | Laberge 2010 [[57]](https://paperpile.com/c/iSaHpS/gksIZ) |
|  |  |  |  | 9 |  | LaDonna 2015 [[58]](https://paperpile.com/c/iSaHpS/hmxQ0) |
| Neurofibromatosis (type not defined) |  |  | 1 | 22 |  | Depping 2021 [[50]](https://paperpile.com/c/iSaHpS/yLaEa) |
| Neurofibromatosis type 1 | 636 | Q85.0 | 3 | 49 |  | Rosnau 2016 [[59]](https://paperpile.com/c/iSaHpS/LVgiH) |
|  |  |  |  | 41 |  | Merker 2018 [[60]](https://paperpile.com/c/iSaHpS/oe9KM) |
|  |  |  |  | 40 |  | Riklin 2017 [[61]](https://paperpile.com/c/iSaHpS/9iUCS) |
| Neurofibromatosis type 2 | 637 | Q85.0 | 2 | 34 |  | Merker 2018 [[60]](https://paperpile.com/c/iSaHpS/oe9KM) |
|  |  |  |  | 33 |  | Riklin 2017 [[61]](https://paperpile.com/c/iSaHpS/9iUCS) |
| Oculopharyngeal muscular dystrophy | 270 | G71.0 | 1 | 25 |  | Kurtz 2019 [[62]](https://paperpile.com/c/iSaHpS/ND6mJ) |
| Phenylketonuria | 716 | E70.0, E70.1 | 1 | 21 |  | Borghi 2020 [[63]](https://paperpile.com/c/iSaHpS/hGAGX) |
| Polyarteritis nodosa | 767 | M30.0 | 1 | 2 |  | Mooney 2013 [[4]](https://paperpile.com/c/iSaHpS/EG7M1) |
| Primary adrenal insufficiency | 101958 | many | 1 | 10 |  | Shepherd 2017 [[64]](https://paperpile.com/c/iSaHpS/EQ0lw) |
| Primary sclerosing cholangitis | 171 | K83.0 | 1 | 41 |  | Depping 2021 [[50]](https://paperpile.com/c/iSaHpS/yLaEa) |
| Pulmonary arterial hypertension | 182090 |  | 2 | 15 |  | Depping 2021 [[50]](https://paperpile.com/c/iSaHpS/yLaEa) |
|  |  |  |  | 3 |  | Kesselheim 2015 [[30]](https://paperpile.com/c/iSaHpS/pdfyS) |
| Rare diseases | not specified | many | 4 | 22 | 12 | Garrino 2015 [[65]](https://paperpile.com/c/iSaHpS/KvRcH) |
|  |  |  |  | 55 | 13 | Litzkendorf 2020 [[66]](https://paperpile.com/c/iSaHpS/jMC68) |
|  |  |  |  | 9 |  | Molster 2021 [[67]](https://paperpile.com/c/iSaHpS/ho6qK) |
|  |  |  |  | 37 |  | Rovira-Moreno 2020 [[68]](https://paperpile.com/c/iSaHpS/pPcdI) |
| Rare abdominal surgical disease | 165711 |  | 1 | 1 |  | Molster 2021 [[67]](https://paperpile.com/c/iSaHpS/ho6qK) |
| Rare allergic disease | 98050 |  | 1 | 17 |  | Molster 2021 [[67]](https://paperpile.com/c/iSaHpS/ho6qK) |
| Rare bone disease | 93419 |  | 2 | 19 |  | Bryson 2021 [[69]](https://paperpile.com/c/iSaHpS/ZhBhJ) |
|  |  |  |  | 5 |  | Molster 2021 [[67]](https://paperpile.com/c/iSaHpS/ho6qK) |
| Rare cardiac disease | 97929 |  | 1 | 2 |  | Bryson 2021 [[69]](https://paperpile.com/c/iSaHpS/ZhBhJ) |
| Rare circulatory system disease | 98028 |  | 1 | 1 |  | Molster 2021 [[67]](https://paperpile.com/c/iSaHpS/ho6qK) |
| Rare developmental defect during embryogenesis | 93890 |  | 1 | 80 |  | Molster 2021 [[67]](https://paperpile.com/c/iSaHpS/ho6qK) |
| Rare endocrine disease | 97978 |  | 2 | 36 |  | Bryson 2021 [[69]](https://paperpile.com/c/iSaHpS/ZhBhJ) |
|  |  |  |  | 39 |  | Molster 2021 [[67]](https://paperpile.com/c/iSaHpS/ho6qK) |
| Rare gastroenterologic disease | 97935 |  | 1 | 21 |  | Molster 2021 [[67]](https://paperpile.com/c/iSaHpS/ho6qK) |
| Rare genetic eye disease | 101435 |  | 2 | 2 |  | Bryson 2021 [[69]](https://paperpile.com/c/iSaHpS/ZhBhJ) |
|  |  |  |  | 1 |  | Molster 2021 [[67]](https://paperpile.com/c/iSaHpS/ho6qK) |
| Rare gynecologic or obstetric disease | 96344 |  | 1 | 1 |  | Molster 2021 [[67]](https://paperpile.com/c/iSaHpS/ho6qK) |
| Rare hematologic disease | 97992 |  | 2 | 11 |  | Bryson 2021 [[69]](https://paperpile.com/c/iSaHpS/ZhBhJ) |
|  |  |  |  | 21 |  | Molster 2021 [[67]](https://paperpile.com/c/iSaHpS/ho6qK) |
| Rare hepatic disease | 57146 |  | 2 | 9 |  | Bryson 2021 [[69]](https://paperpile.com/c/iSaHpS/ZhBhJ) |
|  |  |  |  | 9 |  | Molster 2021 [[67]](https://paperpile.com/c/iSaHpS/ho6qK) |
| Rare immune disease | 98004 |  | 2 | 63 |  | Bryson 2021 [[69]](https://paperpile.com/c/iSaHpS/ZhBhJ) |
|  |  |  |  | 41 |  | Molster 2021 [[67]](https://paperpile.com/c/iSaHpS/ho6qK) |
| Rare inborn errors of metabolism | 68367 |  | 2 | 21 |  | Bryson 2021 [[69]](https://paperpile.com/c/iSaHpS/ZhBhJ) |
|  |  |  |  | 50 |  | Molster 2021 [[67]](https://paperpile.com/c/iSaHpS/ho6qK) |
| Rare infectious disease | 68416 |  | 1 | 13 |  | Bryson 2021 [[69]](https://paperpile.com/c/iSaHpS/ZhBhJ) |
| Rare neoplastic disease | 250908 |  | 2 | 236 |  | Bryson 2021 [[69]](https://paperpile.com/c/iSaHpS/ZhBhJ) |
|  |  |  |  | 11 |  | Molster 2021 [[67]](https://paperpile.com/c/iSaHpS/ho6qK) |
| Rare neurologic disease | 98006 |  | 2 | 501 |  | Bryson 2021 [[69]](https://paperpile.com/c/iSaHpS/ZhBhJ) |
|  |  |  |  | 263 |  | Molster 2021 [[67]](https://paperpile.com/c/iSaHpS/ho6qK) |
| Rare otorhinolaryngologic disease | 98036 |  | 2 | 33 |  | Bryson 2021 [[69]](https://paperpile.com/c/iSaHpS/ZhBhJ) |
|  |  |  |  | 3 |  | Molster 2021 [[67]](https://paperpile.com/c/iSaHpS/ho6qK) |
| Rare pervasive developmental disease | 168778 |  | 1 | 59 |  | Bryson 2021 [[69]](https://paperpile.com/c/iSaHpS/ZhBhJ) |
| Rare renal disease | 93626 |  | 2 | 3 |  | Bryson 2021 [[69]](https://paperpile.com/c/iSaHpS/ZhBhJ) |
|  |  |  |  | 6 |  | Molster 2021 [[67]](https://paperpile.com/c/iSaHpS/ho6qK) |
| Rare respiratory disease | 97955 |  | 2 | 10 |  | Bryson 2021 [[69]](https://paperpile.com/c/iSaHpS/ZhBhJ) |
|  |  |  |  | 55 |  | Molster 2021 [[67]](https://paperpile.com/c/iSaHpS/ho6qK) |
| Rare skin disease | 89826 |  | 2 | 40 |  | Bryson 2021 [[69]](https://paperpile.com/c/iSaHpS/ZhBhJ) |
|  |  |  |  | 22 |  | Molster 2021 [[67]](https://paperpile.com/c/iSaHpS/ho6qK) |
| Rare systemic disease | 182222 |  | 1 | 90 |  | Bryson 2021 [[69]](https://paperpile.com/c/iSaHpS/ZhBhJ) |
| Rare systemic or rheumatologic disease | 98023 |  | 1 | 90 |  | Molster 2021 [[67]](https://paperpile.com/c/iSaHpS/ho6qK) |
| Rare urogenital disease | 101433 |  | 1 | 1 |  | Bryson 2021 [[69]](https://paperpile.com/c/iSaHpS/ZhBhJ) |
| Schwannomatosis | 93921 | Q85.0 | 2 | 7 |  | Merker 2018 [[60]](https://paperpile.com/c/iSaHpS/oe9KM) |
|  |  |  |  | 7 |  | Riklin 2017 [[61]](https://paperpile.com/c/iSaHpS/9iUCS) |
| Scleroderma | 801 |  | 4 | 297 |  | Delisle 2016 [[70]](https://paperpile.com/c/iSaHpS/fQt6i) |
|  |  |  |  | 5 |  | Gumuchian 2018 [[21]](https://paperpile.com/c/iSaHpS/Yj9yv) |
|  |  |  |  | 53 |  | Katavic 2016 [[53]](https://paperpile.com/c/iSaHpS/MLdJN) |
|  |  |  |  | 5 |  | Katavic 2019 [[54]](https://paperpile.com/c/iSaHpS/E1bfM) |
| Spina bifida | 823 | Q05.0-Q05.9 | 3 | 21 | 11 | Lindsay 2016 [[71]](https://paperpile.com/c/iSaHpS/45CHg) |
|  |  |  |  | 74 |  | O`Mahar 2010 [[72]](https://paperpile.com/c/iSaHpS/eJhWD) |
|  |  |  |  | 25 |  | Shoshan 2012 [[73]](https://paperpile.com/c/iSaHpS/iv5OB) |
| Spina bifida aperta | 268369 | Q05.0-Q05.9 | 1 | 37 |  | Stubberud 2015 [[74]](https://paperpile.com/c/iSaHpS/n2aoe) |
| Systemic lupus erythematosus | 536 | M32.0, M32.1, M32.8, M32.9 | 2 | 42 |  | Katavic 2016 [[53]](https://paperpile.com/c/iSaHpS/MLdJN) |
|  |  |  |  | 5 |  | Katavic 2019 [[54]](https://paperpile.com/c/iSaHpS/E1bfM) |
| Takayasu arteritis | 3287 | M31.4 | 1 | 12 |  | Carpenter 2011 [[3]](https://paperpile.com/c/iSaHpS/jRwSA) |
| von Willebrand disease type 1 | 166078 | D68.0 | 1 | 1 |  | Arya 2021 [[5]](https://paperpile.com/c/iSaHpS/t1A48) |
| von Willebrand disease type 2 | 166081 | D68.0 | 1 | 3 |  | Arya 2021 [[5]](https://paperpile.com/c/iSaHpS/t1A48) |
| Vulvar epidermal neoplasia | 137583 | D07.1 | 1 | 49 |  | Raphaelis 2018 [[75]](https://paperpile.com/c/iSaHpS/HNXhZ) |
| Wegener's granulomatosis (Granulomatosis with polyangiitis) | 900 | M31.3 | 2 | 138 |  | Carpenter 2011 [[3]](https://paperpile.com/c/iSaHpS/jRwSA) |
|  |  |  |  | 9 |  | Mooney 2013 [[4]](https://paperpile.com/c/iSaHpS/EG7M1) |
|  |  |  | Sum | 6223 | 1707 |  |

[1. Mälstam E, Bensing S, Asaba E. Everyday managing and living with autoimmune Addison’s disease: Exploring experiences using photovoice methods. Scand J Occup Ther [Internet]. 2018;25:358–70. Available from:](http://paperpile.com/b/iSaHpS/uP8In) <http://dx.doi.org/10.1080/11038128.2018.1502351>

[2. Socha Hernandez AV, Deeks LS, Shield AJ. Understanding medication safety and Charcot-Marie-Tooth disease: a patient perspective. Int J Clin Pharm [Internet]. 2020;42:1507–14. Available from:](http://paperpile.com/b/iSaHpS/Gj58s) <http://dx.doi.org/10.1007/s11096-020-01123-z>

[3. Carpenter DM, DeVellis RF, Hogan SL, Fisher EB, DeVellis BM, Jordan JM. Use and perceived credibility of medication information sources for patients with a rare illness: differences by gender. J Health Commun [Internet]. 2011;16:629–42. Available from:](http://paperpile.com/b/iSaHpS/jRwSA) <http://dx.doi.org/10.1080/10810730.2011.551995>

[4. Mooney J, Poland F, Spalding N, Scott DGI. “In One Ear and Out the Other–It”s a Lot to Take in’: A Qualitative Study Exploring the Informational Needs of Patients with ANCA‐Associated Vasculitis. Musculoskeletal [Internet]. 2013; Available from:](http://paperpile.com/b/iSaHpS/EG7M1) <https://onlinelibrary.wiley.com/doi/abs/10.1002/msc.1030>

[5. Arya S, Wilton P, Page D, Boma-Fischer L, Floros G, Winikoff R, et al. “They don’t really take my bleeds seriously”: Barriers to care for women with inherited bleeding disorders. J Thromb Haemost [Internet]. 2021;19:1506–14. Available from:](http://paperpile.com/b/iSaHpS/t1A48) <https://www.sciencedirect.com/science/article/pii/S1538783622007905>

[6. Dwyer AA, Quinton R, Morin D, Pitteloud N. Identifying the unmet health needs of patients with congenital hypogonadotropic hypogonadism using a web-based needs assessment: implications for online interventions and peer-to-peer support. Orphanet J Rare Dis [Internet]. 2014;9:83. Available from:](http://paperpile.com/b/iSaHpS/edkmx) <https://ojrd.biomedcentral.com/articles/10.1186/1750-1172-9-83>

[7. Beinke K, O’Callaghan F, Morrissey S. Illness Perceptions of Cystic Fibrosis: A Comparison of Young Adults with CF and Same-Aged Peers. Behav Med [Internet]. 2017;43:40–6. Available from:](http://paperpile.com/b/iSaHpS/jj0Rb) <http://dx.doi.org/10.1080/08964289.2015.1045824>

[8. Chaudhry SR, Keaton M, Nasr SZ. Evaluation of a cystic fibrosis transition program from pediatric to adult care. Pediatr Pulmonol [Internet]. 2013;48:658–65. Available from:](http://paperpile.com/b/iSaHpS/nFDQh) <http://dx.doi.org/10.1002/ppul.22647>

[9. David V, Feldman D, Danner-Boucher I, Rhun AL, Guyomarch B, Ravilly S, et al. Identifying the educational needs of lung transplant recipients with cystic fibrosis. Prog Transplant [Internet]. 2015;25:18–25. Available from:](http://paperpile.com/b/iSaHpS/8AfYl) <http://dx.doi.org/10.7182/pit2015526>

[10. Dellon EP, Helms SW, Hailey CE, Shay R, Carney SD, Schmidt HJ, et al. Exploring knowledge and perceptions of palliative care to inform integration of palliative care education into cystic fibrosis care. Pediatr Pulmonol [Internet]. 2018;53:1218–24. Available from:](http://paperpile.com/b/iSaHpS/EKEYE) <http://dx.doi.org/10.1002/ppul.24073>

[11. Flewelling KD, Sellers DE, Sawicki GS, Robinson WM, Dill EJ. Social support is associated with fewer reported symptoms and decreased treatment burden in adults with cystic fibrosis. J Cyst Fibros [Internet]. 2019;18:572–6. Available from:](http://paperpile.com/b/iSaHpS/beAHp) <http://dx.doi.org/10.1016/j.jcf.2019.01.013>

[12. Ioannou L, Massie J, Collins V, McClaren B, Delatycki MB. Population-based genetic screening for cystic fibrosis: attitudes and outcomes. Public Health Genomics [Internet]. 2010;13:449–56. Available from:](http://paperpile.com/b/iSaHpS/XnKyc) <http://dx.doi.org/10.1159/000276544>

[13. Jackson AD, Kirwan L, Gibney S, Jeleniewska P, Fletcher G, Doyle G. Associations between health literacy and patient outcomes in adolescents and young adults with cystic fibrosis. Eur J Public Health [Internet]. 2020;30:112–8. Available from:](http://paperpile.com/b/iSaHpS/jsDY3) <http://dx.doi.org/10.1093/eurpub/ckz148>

[14. Kazmerski TM, Gmelin T, Slocum B, Borrero S, Miller E. Attitudes and Decision Making Related to Pregnancy Among Young Women with Cystic Fibrosis. Matern Child Health J [Internet]. 2017;21:818–24. Available from:](http://paperpile.com/b/iSaHpS/ohqhm) <http://dx.doi.org/10.1007/s10995-016-2181-z>

[15. Keyte R, Egan H, Nash EF, Regan A, Jackson C, Mantzios M. An exploration into experiences and attitudes regarding risky health behaviours in an adult cystic fibrosis population. Psychol Health Med [Internet]. 2020;25:1013–9. Available from:](http://paperpile.com/b/iSaHpS/FFJnW) <http://dx.doi.org/10.1080/13548506.2019.1706750>

[16. Kirk S, Milnes L. An exploration of how young people and parents use online support in the context of living with cystic fibrosis. Health Expect [Internet]. 2016;19:309–21. Available from:](http://paperpile.com/b/iSaHpS/YmSnm) <http://dx.doi.org/10.1111/hex.12352>

[17. Lewis KL, John B, Condren M, Carter SM. Evaluation of Medication-related Self-care Skills in Patients With Cystic Fibrosis. J Pediatr Pharmacol Ther [Internet]. 2016;21:502–11. Available from:](http://paperpile.com/b/iSaHpS/3t4dl) <http://dx.doi.org/10.5863/1551-6776-21.6.502>

[18. Lonabaugh KP, O’Neal KS, McIntosh H, Condren M. Cystic fibrosis-related education: Are we meeting patient and caregiver expectations? Patient Educ Couns [Internet]. 2018;101:1865–70. Available from:](http://paperpile.com/b/iSaHpS/i4BJ8) <http://dx.doi.org/10.1016/j.pec.2018.06.004>

[19. Pakhale S, Baron J, Armstrong M, Tasca G, Gaudet E, Aaron SD, et al. Lost in translation? How adults living with Cystic Fibrosis understand treatment recommendations from their healthcare providers, and the impact on adherence to therapy. Patient Educ Couns [Internet]. 2016;99:1319–24. Available from:](http://paperpile.com/b/iSaHpS/ywma5) <http://dx.doi.org/10.1016/j.pec.2016.03.023>

[20. Sylvain C, Lamothe L, Berthiaume Y, Rabasa-Lhoret R. How patients’ representations of cystic fibrosis-related diabetes inform their health behaviours. Psychol Health [Internet]. 2016;31:1129–44. Available from:](http://paperpile.com/b/iSaHpS/Eg2D9) <http://dx.doi.org/10.1080/08870446.2016.1183008>

[21. Gumuchian ST, Peláez S, Delisle VC, Carrier M-E, Jewett LR, El-Baalbaki G, et al. Understanding coping strategies among people living with scleroderma: a focus group study. Disabil Rehabil [Internet]. 2018;40:3012–21. Available from:](http://paperpile.com/b/iSaHpS/Yj9yv) <http://dx.doi.org/10.1080/09638288.2017.1365954>

[22. Hiermeier UM, Baker C, Bourke JP. Exploring the acceptability of implantable defibrillators in patients with cardiac dystrophinopathy and carers. Open Heart [Internet]. 2020;7:e001230. Available from:](http://paperpile.com/b/iSaHpS/P1xsD) <http://dx.doi.org/10.1136/openhrt-2019-001230>

[23. Parvizi MM, Lankarani KB, Handjani F, Ghahramani S, Parvizi Z, Rousta S. Health literacy in patients with epidermolysis bullosa in Iran. J Educ Health Promot [Internet]. 2017;6:105. Available from:](http://paperpile.com/b/iSaHpS/jjcOp) <http://dx.doi.org/10.4103/jehp.jehp_64_17>

[24. Naik H, Shenbagam S, Go AM, Balwani M. Psychosocial issues in erythropoietic protoporphyria - the perspective of parents, children, and young adults: A qualitative study. Mol Genet Metab [Internet]. 2019;128:314–9. Available from:](http://paperpile.com/b/iSaHpS/CX99b) <http://dx.doi.org/10.1016/j.ymgme.2019.01.023>

[25. Théaudin M, Cauquil C, Antonini T, Algalarrondo V, Labeyrie C, Aycaguer S, et al. Familial amyloid polyneuropathy: elaboration of a therapeutic patient education programme, “EdAmyl.” Amyloid [Internet]. 2014;21:225–30. Available from:](http://paperpile.com/b/iSaHpS/9gH2j) <http://dx.doi.org/10.3109/13506129.2014.941463>

[26. Salvatore V, Gilstrap A, Williams KR, Thorat S, Stevenson M, Gwosdow AR, et al. Evaluating the impact of peer support and connection on the quality of life of patients with familial chylomicronemia syndrome. Expert Opinion on Orphan Drugs [Internet]. 2018;6:497–505. Available from:](http://paperpile.com/b/iSaHpS/meKVh) <https://doi.org/10.1080/21678707.2018.1505495>

[27. Smolich L, Charen K, Sherman SL. Health knowledge of women with a fragile X premutation: Improving understanding with targeted educational material. J Genet Couns [Internet]. 2020;29:983–91. Available from:](http://paperpile.com/b/iSaHpS/DrqIz) <http://dx.doi.org/10.1002/jgc4.1222>

[28. Walsh MB, Charen K, Shubeck L, McConkie-Rosell A, Ali N, Bellcross C, et al. Men with an FMR1 premutation and their health education needs. J Genet Couns [Internet]. 2021;30:1156–67. Available from:](http://paperpile.com/b/iSaHpS/0cDXL) <http://dx.doi.org/10.1002/jgc4.1399>

[29. Akanuwe JNA, Laparidou D, Curtis F, Jackson J, Hodgson TL, Siriwardena AN. Exploring the experiences of having Guillain-Barré Syndrome: A qualitative interview study. Health Expect [Internet]. 2020;23:1338–49. Available from:](http://paperpile.com/b/iSaHpS/RUCcm) <https://onlinelibrary.wiley.com/doi/10.1111/hex.13116>

[30. Kesselheim AS, McGraw S, Thompson L, O’Keefe K, Gagne JJ. Development and use of new therapeutics for rare diseases: views from patients, caregivers, and advocates. Patient [Internet]. 2015;8:75–84. Available from:](http://paperpile.com/b/iSaHpS/pdfyS) <http://dx.doi.org/10.1007/s40271-014-0096-6>

[31. le Doré S, Grinda N, Ferré E, Roussel-Robert V, Frotscher B, Chamouni P, et al. The hemarthrosis-simulating knee model: A useful tool for individualized education in patients with hemophilia (GEFACET study). J Blood Med [Internet]. 2021;12:133–8. Available from:](http://paperpile.com/b/iSaHpS/pSCR8) <https://www.tandfonline.com/doi/abs/10.2147/JBM.S280032>

[32. Mohan R, Radhakrishnan N, Varadarajan M, Anand S. Assessing the current knowledge, attitude and behaviour of adolescents and young adults living with haemophilia. Haemophilia [Internet]. 2021;27:e180–6. Available from:](http://paperpile.com/b/iSaHpS/TlASj) <http://dx.doi.org/10.1111/hae.14229>

[33. Torres-Ortuño A, Cuesta-Barriuso R, Nieto-Munuera J, Galindo-Piñana P, López-Pina J-A. The behaviour and perception of illness: modulating variables of adherence in patients with haemophilia. Vox Sang [Internet]. 2018; Available from:](http://paperpile.com/b/iSaHpS/R5a1B) <http://dx.doi.org/10.1111/vox.12669>

[34. Lindvall K, Colstrup L, Loogna K, Wollter I, Grönhaug S. Knowledge of disease and adherence in adult patients with haemophilia. Haemophilia [Internet]. 2010;16:592–6. Available from:](http://paperpile.com/b/iSaHpS/PeeTo) <http://dx.doi.org/10.1111/j.1365-2516.2009.02189.x>

[35. Arnold E, Lane S, Webert KE, Chan A, Walker I, Tufts J, et al. What should men living with haemophilia need to know? The perspectives of Canadian men with haemophilia. Haemophilia [Internet]. 2014;20:219–25. Available from:](http://paperpile.com/b/iSaHpS/oNVM2) <https://onlinelibrary.wiley.com/doi/10.1111/hae.12297>

[36. De la Corte-Rodriguez H, Rodriguez-Merchan EC, Alvarez-Roman T, Martin-Salces M, Garcia-Barcenilla S, Jimenez-Yuste V. Health education and empowerment in adult patients with haemophilia. Expert Rev Hematol [Internet]. 2019;12:989–95. Available from:](http://paperpile.com/b/iSaHpS/63lsY) <http://dx.doi.org/10.1080/17474086.2019.1650640>

[37. Mulders G, de Wee EM, Vahedi Nikbakht-Van de Sande MCVM, Kruip MJHA, Elfrink EJ, Leebeek FWG. E-learning improves knowledge and practical skills in haemophilia patients on home treatment: a randomized controlled trial. Haemophilia [Internet]. 2012;18:693–8. Available from:](http://paperpile.com/b/iSaHpS/CWJcI) <https://onlinelibrary.wiley.com/doi/10.1111/j.1365-2516.2012.02786.x>

[38. van Balen EC, Krawczyk M, Gue D, Jackson S, Gouw SC, van der Bom JG, et al. Patient-centred care in haemophilia: Patient perspectives on visualization and participation in decision-making. Haemophilia [Internet]. 2019;25:938–45. Available from:](http://paperpile.com/b/iSaHpS/fm2Kq) <http://dx.doi.org/10.1111/hae.13830>

[39. Bhatt N, Boggio L, Simpson ML. Using an educational intervention to assess and improve disease-specific knowledge and health literacy and numeracy in adolescents and young adults with haemophilia A and B. Haemophilia [Internet]. 2021;27:229–36. Available from:](http://paperpile.com/b/iSaHpS/UVMfW) <https://onlinelibrary.wiley.com/doi/abs/10.1111/hae.14228>

[40. Hoefnagels JW, Fischer K, Bos RAT, Driessens MHE, Meijer SLA, Schutgens REG, et al. A feasibility study on two tailored interventions to improve adherence in adults with haemophilia. Pilot Feasibility Stud [Internet]. 2020;6:189. Available from:](http://paperpile.com/b/iSaHpS/GHYFY) <http://dx.doi.org/10.1186/s40814-020-00723-w>

[41. Arran N, Craufurd D, Simpson J. Illness perceptions, coping styles and psychological distress in adults with Huntington’s disease. Psychol Health Med [Internet]. 2014;19:169–79. Available from:](http://paperpile.com/b/iSaHpS/Fu5nj) <http://dx.doi.org/10.1080/13548506.2013.802355>

[42. Braisch U, Martinez-Horta S, MacDonald M, Orth M. Important but not enough - information about HD related topics and peer and professional support for young adults from HD families. J Huntingtons Dis [Internet]. 2016 [cited 2023 Jun 6];5:379–87. Available from:](http://paperpile.com/b/iSaHpS/R9hJi) <https://content.iospress.com/articles/journal-of-huntingtons-disease/jhd160218>

[43. Domaradzki J. Family caregivers’ experiences with healthcare services--a case of Huntington’s disease. Psychiatr Pol [Internet]. 2016;50:375–91. Available from:](http://paperpile.com/b/iSaHpS/VICQF) <http://dx.doi.org/10.12740/PP/59103>

[44. Etchegary H. Healthcare experiences of families affected by Huntington disease: need for improved care. Chronic Illn [Internet]. 2011;7:225–38. Available from:](http://paperpile.com/b/iSaHpS/H1QKo) <http://dx.doi.org/10.1177/1742395311403637>

[45. Ringqvist K, Borg K, Möller MC. Tolerability and psychological effects of a multimodal day-care rehabilitation program for persons with Huntington’s disease. J Rehabil Med [Internet]. 2021;53:jrm00143. Available from:](http://paperpile.com/b/iSaHpS/XlHCb) <http://dx.doi.org/10.2340/16501977-2748>

[46. Skirton H, Williams JK, Jackson Barnette J, Paulsen JS. Huntington disease: families’ experiences of healthcare services. J Adv Nurs [Internet]. 2010;66:500–10. Available from:](http://paperpile.com/b/iSaHpS/fOyps) <http://dx.doi.org/10.1111/j.1365-2648.2009.05217.x>

[47. Smedley RM, Coulson NS. Genetic testing for Huntington’s disease: A thematic analysis of online support community messages. J Health Psychol [Internet]. 2021;26:580–94. Available from:](http://paperpile.com/b/iSaHpS/8OAv1) <http://dx.doi.org/10.1177/1359105319826340>

[48. Chaleat-Valayer E, Amélie Z, Marie-Hélène B, Perretant I, Sandrine T. Therapeutic education program for patients with hypermobile Ehlers-Danlos syndrome: Feasibility and satisfaction of the participants. Education thérapeutique du patient - Therapeutic patient education [Internet]. 2019 [cited 2023 Jun 14];11:10202. Available from:](http://paperpile.com/b/iSaHpS/552OA) <https://www.researchgate.net/publication/332063782_Therapeutic_education_program_for_patients_with_hypermobile_Ehlers-Danlos_syndrome_Feasibility_and_satisfaction_of_the_participants>

[49. Takeuchi T, Muraoka K, Yamada M, Nishio Y, Hozumi I. Living with idiopathic basal ganglia calcification 3: a qualitative study describing the lives and illness of people diagnosed with a rare neurological disease. Springerplus [Internet]. 2016;5:1713. Available from:](http://paperpile.com/b/iSaHpS/xZeI8) <http://dx.doi.org/10.1186/s40064-016-3390-z>

[50. Depping MK, Uhlenbusch N, Härter M, Schramm C, Löwe B. Efficacy of a Brief, Peer-Delivered Self-management Intervention for Patients With Rare Chronic Diseases: A Randomized Clinical Trial. JAMA Psychiatry [Internet]. 2021;78:607–15. Available from:](http://paperpile.com/b/iSaHpS/yLaEa) <http://dx.doi.org/10.1001/jamapsychiatry.2020.4783>

[51. Bogart KR, Frandrup E, Locke T, Thompson H, Weber N, Yates J, et al. “Rare place where I feel normal”: Perceptions of a social support conference among parents of and people with Moebius syndrome. Res Dev Disabil [Internet]. 2017;64:143–51. Available from:](http://paperpile.com/b/iSaHpS/liMJM) <https://www.sciencedirect.com/science/article/pii/S0891422217300963>

[52. Foley G, Timonen V, Hardiman O. Understanding psycho-social processes underpinning engagement with services in motor neurone disease: a qualitative study. Palliat Med [Internet]. 2014;28:318–25. Available from:](http://paperpile.com/b/iSaHpS/1hHHE) <http://dx.doi.org/10.1177/0269216313512013>

[53. Katavic SS, Tanackovic SF, Badurina B. Illness perception and information behaviour of patients with rare chronic diseases. Inflamm Res [Internet]. 2016 [cited 2023 Jun 14];21. Available from:](http://paperpile.com/b/iSaHpS/MLdJN) <http://dx.doi.org/10.1111/hir.12261>

[54. Stanarević Katavić S. Health information behaviour of rare disease patients: seeking, finding and sharing health information. Health Info Libr J [Internet]. 2019;36:341–56. Available from:](http://paperpile.com/b/iSaHpS/E1bfM) <http://dx.doi.org/10.1111/hir.12261>

[55. Dicianno BE, Lovelace J, Peele P, Fassinger C, Houck P, Bursic A, et al. Effectiveness of a Wellness Program for Individuals With Spina Bifida and Spinal Cord Injury Within an Integrated Delivery System. Arch Phys Med Rehabil [Internet]. 2016;97:1969–78. Available from:](http://paperpile.com/b/iSaHpS/8yWpK) <http://dx.doi.org/10.1016/j.apmr.2016.05.014>

[56. Coathup V, Teare HJA, Minari J, Yoshizawa G, Kaye J, Takahashi MP, et al. Using digital technologies to engage with medical research: views of myotonic dystrophy patients in Japan. BMC Med Ethics [Internet]. 2016;17:51. Available from:](http://paperpile.com/b/iSaHpS/0HQIP) <http://dx.doi.org/10.1186/s12910-016-0132-2>

[57. Laberge L, Prévost C, Perron M, Mathieu J, Auclair J, Gaudreault M, et al. Clinical and genetic knowledge and attitudes of patients with myotonic dystrophy type 1. Public Health Genomics [Internet]. 2010;13:424–30. Available from:](http://paperpile.com/b/iSaHpS/gksIZ) <http://dx.doi.org/10.1159/000316238>

[58. LaDonna KA, Ghavanini AA, Venance SL. Truths and misinformation: a qualitative exploration of myotonic dystrophy. Can J Neurol Sci [Internet]. 2015;42:187–94. Available from:](http://paperpile.com/b/iSaHpS/hmxQ0) <http://dx.doi.org/10.1017/cjn.2015.26>

[59. Rosnau K, Hashmi SS, Northrup H, Slopis J, Noblin S, Ashfaq M. Knowledge and Self-Esteem of Individuals with Neurofibromatosis Type 1 (NF1). J Genet Couns [Internet]. 2017;26:620–7. Available from:](http://paperpile.com/b/iSaHpS/LVgiH) <http://dx.doi.org/10.1007/s10897-016-0036-9>

[60. Merker VL, McDannold S, Riklin E, Talaei-Khoei M, Sheridan MR, Jordan JT, et al. Health literacy assessment in adults with neurofibromatosis: electronic and short-form measurement using FCCHL and Health LiTT. J Neurooncol [Internet]. 2018;136:335–42. Available from:](http://paperpile.com/b/iSaHpS/oe9KM) <http://dx.doi.org/10.1007/s11060-017-2657-8>

[61. Riklin E, Talaei-Khoei M, Merker VL, Sheridan MR, Jordan JT, Plotkin SR, et al. First report of factors associated with satisfaction in patients with neurofibromatosis. Am J Med Genet A [Internet]. 2017;173:671–7. Available from:](http://paperpile.com/b/iSaHpS/9iUCS) <http://dx.doi.org/10.1002/ajmg.a.38079>

[62. Kurtz NS, Cote C, Heatwole C, Gagnon C, Youssof S. Patient-reported disease burden in oculopharyngeal muscular dystrophy. Muscle Nerve [Internet]. 2019;60:724–31. Available from:](http://paperpile.com/b/iSaHpS/ND6mJ) <https://onlinelibrary.wiley.com/doi/abs/10.1002/mus.26712>

[63. Borghi L, Moreschi C, Toscano A, Comber P, Vegni E. The PKU & ME study: A qualitative exploration, through co-creative sessions, of attitudes and experience of the disease among adults with phenylketonuria in Italy. Molecular Genetics and Metabolism Reports [Internet]. 2020;23:100585. Available from:](http://paperpile.com/b/iSaHpS/hGAGX) <https://www.sciencedirect.com/science/article/pii/S2214426920300318>

[64. Shepherd LM, Tahrani AA, Inman C, Arlt W, Carrick-Sen DM. Exploration of knowledge and understanding in patients with primary adrenal insufficiency: a mixed methods study. BMC Endocr Disord [Internet]. 2017;17:47. Available from:](http://paperpile.com/b/iSaHpS/EQ0lw) <http://dx.doi.org/10.1186/s12902-017-0196-0>

[65. Garrino L, Picco E, Finiguerra I, Rossi D, Simone P, Roccatello D. Living with and treating rare diseases: experiences of patients and professional health care providers. Qual Health Res [Internet]. 2015;25:636–51. Available from:](http://paperpile.com/b/iSaHpS/KvRcH) <http://dx.doi.org/10.1177/1049732315570116>

[66. Litzkendorf S, Frank M, Babac A, Rosenfeldt D, Schauer F, Hartz T, et al. Use and importance of different information sources among patients with rare diseases and their relatives over time: a qualitative study. BMC Public Health [Internet]. 2020;20:860. Available from:](http://paperpile.com/b/iSaHpS/jMC68) <http://dx.doi.org/10.1186/s12889-020-08926-9>

[67. Molster C, Urwin D, Di Pietro L, Fookes M, Petrie D, van der Laan S, et al. Survey of healthcare experiences of Australian adults living with rare diseases. Orphanet J Rare Dis [Internet]. 2016;11:30. Available from:](http://paperpile.com/b/iSaHpS/ho6qK) <http://dx.doi.org/10.1186/s13023-016-0409-z>

[68. Rovira-Moreno E, Abuli A, Codina-Sola M, Valenzuela I, Serra-Juhe C, Cuscó I, et al. Beyond the disease itself: A cross-cutting educational initiative for patients and families with rare diseases. J Genet Couns [Internet]. 2021;30:693–700. Available from:](http://paperpile.com/b/iSaHpS/pPcdI) <http://dx.doi.org/10.1002/jgc4.1354>

[69. Bryson B, Bogart K, Atwood M, Fraser K, Locke T, Pugh K, et al. Navigating the unknown: A content analysis of the unique challenges faced by adults with rare diseases. J Health Psychol [Internet]. 2021;26:623–35. Available from:](http://paperpile.com/b/iSaHpS/ZhBhJ) <http://dx.doi.org/10.1177/1359105319828150>

[70. Delisle VC, Gumuchian ST, Pelaez S, Malcarne VL, El-Baalbaki G, Körner A, et al. Reasons for non-participation in scleroderma support groups. Clin Exp Rheumatol [Internet]. 2016;34 Suppl 100:56–62. Available from:](http://paperpile.com/b/iSaHpS/fQt6i) <https://www.ncbi.nlm.nih.gov/pubmed/26950221>

[71. Lindsay S, Fellin M, Cruickshank H, McPherson A, Maxwell J. Youth and parents’ experiences of a new inter-agency transition model for spina bifida compared to youth who did not take part in the model. Disabil Health J [Internet]. 2016;9:705–12. Available from:](http://paperpile.com/b/iSaHpS/45CHg) <http://dx.doi.org/10.1016/j.dhjo.2016.05.009>

[72. O’Mahar K, Holmbeck GN, Jandasek B, Zukerman J. A camp-based intervention targeting independence among individuals with spina bifida. J Pediatr Psychol [Internet]. 2010;35:848–56. Available from:](http://paperpile.com/b/iSaHpS/eJhWD) <http://dx.doi.org/10.1093/jpepsy/jsp125>

[73. Shoshan L, Ben-Zvi D, Meyer S, Katz-Leurer M. Sexuality in relation to independence in daily functions among young people with spina bifida living in Israel. Rehabil Nurs [Internet]. 2012;37:11–7; quiz 17–8. Available from:](http://paperpile.com/b/iSaHpS/iv5OB) <http://dx.doi.org/10.1002/RNJ.00002>

[74. Stubberud J, Langenbahn D, Levine B, Stanghelle J, Schanke A-K. Emotional health and coping in spina bifida after goal management training: a randomized controlled trial. Rehabil Psychol [Internet]. 2015;60:1–16. Available from:](http://paperpile.com/b/iSaHpS/n2aoe) <http://dx.doi.org/10.1037/rep0000018>

[75. Raphaelis S, Mayer H, Ott S, Hornung R, Senn B. Effects of Written Information and Counseling on Illness-Related Uncertainty in Women With Vulvar Neoplasia. Oncol Nurs Forum [Internet]. 2018;45:748–60. Available from:](http://paperpile.com/b/iSaHpS/HNXhZ) <http://dx.doi.org/10.1188/18.ONF.748-760>
