# Supplementary material for: A scoping review of health literacy in rare disorders: key issues and research directions
Source: Orphanet J Rare Dis. 2024 Sep 6;19:328. doi: 10.1186/s13023-024-03332-5 (PMC11380335; doi:10.1186/s13023-024-03332-5)
Supplement: Supplementary file 5 — Supplementary Material 5 [file 13023_2024_3332_MOESM5_ESM.docx]

Additional file 5

Table 3. Data extraction of included reports.

| **First Author**  **Year**  **Coun-try** | **Aim of Study** | **Study Design**  | **«Health Literacy definitions» or understanding of relevance to this review** | **Author-reported results** |
| --- | --- | --- | --- | --- |
| Akanuwe 2020  UK [[1]](https://paperpile.com/c/jHvZKb/yyhKq) | To explore the experiences of people with Guillain-Barrè Syndrome in the UK | Qualitative: face-to-face and telephone interviews | How effective care influence coping and understanding of own health status  Communication as an integral part of care | Early diagnosis and treatment: Participants emphasized that early diagnosis and treatment helped speed up the recovery and prevented potential medical complications.  Safe and effective inpatient care: Participants reported that receiving safe and effective inpatient care improved their expectations and strengthened coping strategies and the understanding of their health status.  Effective communication: Effective communication was an important factor in both patient satisfaction and complaints. Participants expressed concerns about poor communication and information provision, and improved communication was identified as an important factor for high-quality healthcare experiences. |
| Arnold  2014  Canada [[2]](https://paperpile.com/c/jHvZKb/R0xJi) | To determine current knowledge levels, needs and gaps among Canadian individuals with haemophilia to facilitate optimal disease management | Quantitative: cross-sectional survey | Knowledge levels and knowledge seeking were measured | Positive correlation between knowledge seeking and disease severity: The report found a positive association between the severity of the disease and the participants' motivation to seek knowledge about it, suggesting that individuals with more severe conditions are more eager to educate themselves about their illness.  Targeting specific areas for knowledge sharing: The report pinpointed certain areas that should be targeted in knowledge-sharing initiatives, such as timing of infusions, sexual activity and ageing. |
| Arran  2014  UK [[3]](https://paperpile.com/c/jHvZKb/vwWIQ) | To explore the biopsychosocial factors related to psychological distress in people with Huntington's disease, and further examine the relationship between illness perceptions, coping and psychological distress | Quantitative: cross-sectional survey | Illness perceptions  Feelings of control of the illness | Strong illness identity and perceived negative consequences: The report revealed that individuals with Huntington's had an illness perception that was characterised by a strong illness identity and that they perceived negative consequences and beliefs that their illness had a long disease duration.  Perception of limited control: Participants reported a lack of personal control, as well as a lack of control over the treatment of their illness. |
| Arya  2021  Canada [[4]](https://paperpile.com/c/jHvZKb/KCG5d) | To evaluate and describe barriers to care for women with bleeding disorders | Qualitative: telephone interviews analyzed with thematic analysis | Health-care providers awareness of symptoms  Access to care  Needs for self-education | Perceived barriers to care:  Lack of healthcare provider awareness of bleeding disorders.  Health-care provider dismissal of symptoms.  Limited access to specialized care and treatment plans.  Need for self-education and advocacy. |
| Beinke  2017  Australia [[5]](https://paperpile.com/c/jHvZKb/Rh7q9) | To identify whether individuals  who have known someone with Cystic Fibrosis have illness perceptions that are more similar to those of Cystic Fibrosis patients or to those of individuals without experience with Cystic Fibrosis | Quantitative: cross-sectional survey | Knowledge and perception of diagnosis  Experience with diagnose and social support | Positive illness perception: The report revealed that individuals with Cystic Fibrosis generally hold the most positive illness perception, followed by peers who had experience with knowing someone with Cystic Fibrosis, and then peers without experience with Cystic Fibrosis.  Need for public education and tailored support: The findings highlighted that public education and tailoring of the support given by peers is needed to improve the social support received by adolescents and young adults with cystic fibrosis. |
| Bhatt  2021  USA [[6]](https://paperpile.com/c/jHvZKb/r4OHq) | To test the ability of an educational intervention to improve knowledge, health literacy, health numeracy, adherence and joint health in persons with haemophilia | Quantitative: single center, longitudinal cohort pilot study with a pre-post assessment | Health literacy defined as:  ‘the capacity of individuals to obtain, process and understand the basic health information and services needed to make appropriate health decisions’ (US Department of Health and Human Services)  ‘the cognitive and social skills which determine the motivation and ability of individuals to gain access to, understand and use information in ways which promote and maintain good health’ (World Health Organization)  Health numeracy is a component of health literacy and is defined as ‘one's ability to handle basic probability and numerical concepts, including computations, estimation, logic and problem solving | Benefits of audiovisual education for participants with low health literacy and numeracy: The report found that participants with low health literacy and/or health numeracy may benefit from alternate methods of education such as audiovisual material.  Limited impact of audiovisual education on therapy adherence: While education using audiovisual materials improved knowledge and health numeracy in this study; however, this did not affect adherence to prescribed therapy. |
| Bogart  2017  USA [[7]](https://paperpile.com/c/jHvZKb/uxUeK) | To examine reasons for attending, benefits, and limitations of support conferences for persons with Moebius syndrome | Quantitative: cross-sectional online survey | Companionship and informational support  Mutual learning and insight | Companionship and support: Companionship and informational support were most frequently mentioned as reasons for and benefits of attending. Participants felt normalized by being surrounded by others like them. The companionship support obtained at the conference reduced feelings of isolation. Participants also mentioned emotional support benefits, including support for anxiety, depression, self-esteem, social skills, “feeling understood,” and “gain[ing] confidence.”. Participants also sought instrumental support in the form of speech and occupational therapy, or surgery consultations.  Insight and understanding: Being around others with Moebius syndrome also provided insight into seeing how others see them. People with Moebius syndrome revealed a unique feeling of “being understood.”  Reciprocity of support: Although many participants initially attended to gain support, over time they also reported offering their support and encouragement to others. |
| Borghi  2020  Italy [[8]](https://paperpile.com/c/jHvZKb/aSxtO) | To explore the experience of adults living with phenylketonuria in order to gain insights related to their adherence to diet and engagement in managing their condition | Qualitative: co-creation exercises with focus groups analyzed using content analysis | Needs and wishes regarding communication and information in health care settings  Engagement facilitators in managing phenylketonuria | Adherence and positive mindset: Besides the behavioral dictates of following a strict diet, adherent adults reported a positive mental approach and organizational rigor; they seemed aware of the consequences of high-phenylalanine levels, reporting that it can affect mood and consequently social interactions which they value highly.  Non-adherence and lack of acceptance: In the non-adherent group, the individuals seemed to not fully accept their disease: they were aware of the consequences of non-adherence in children but not in adults, they felt the management of PKU was an individual burden and they experienced a feeling of ‘diversity’ in the social context (related to eating) that caused emotional distress.  Desire for dedicated healthcare: All participants expressed the desire for assistance in a healthcare setting specifically dedicated to adults with PKU. |
| Braisch  2016  Germany [[9]](https://paperpile.com/c/jHvZKb/TUYBT) | To survey the opinion of young adults from Huntington’s disease families about the relevance and availability of information and support regarding several aspects of the disease | Quantitative: cross-sectional online survey | Information about the disease  General information  Information about research  Peer and professional support | Importance of information and support: Information about disease-related topics and the availability of peer and professional support are crucial for young adults.  Insufficient information and support: The majority of respondents stated that they lacked sufficient information and support. |
| Bryson  2021  USA [[10]](https://paperpile.com/c/jHvZKb/aJkrj) | To explore, in participants’ own words, the nature and frequency of challenges experienced by people with rare diseases and how they differ across the following factors identified in the introduction as potentially important: disease categories, gender, years since diagnosis, and symptom duration | Multi method: a cross-sectional survey with open-ended questions analyzed with content analysis | Access to information  Social support  Information of diagnose/prognose  Difficult to find doctors with knowledge about rare diseases  Doctor-patient communication  Awareness of the disease | Management of symptoms and activity limitations: Individuals with rare diseases face challenges in managing various symptoms, including living with pain, fatigue, weakness and physical changes. These symptoms often lead to activity limitations, including environmental barriers and the inability to engage in solitary leisure activities. Difficulties in finding the right treatments, medications, tests and procedures while simultaneously dealing with the uncertainty or fear about the future, misdiagnosis, or disease progression also contribute to the complexity of managing their condition.  Lack of information and support: Participants expressed a lack of informational support, including a lack of received information/education on symptoms, course, and treatment options. They also mentioned receiving unhelpful information from well-intended sources, such as friends and family, that might de-legitimize the disease or in other ways be unhelpful for the individual with a rare disease. Additionally, many reported difficulties in finding doctors knowledgeable in rare diseases. Another thing mentioned was issues in doctor-patient communication, and problems with healthcare providers listening to, believing, and understanding patients, leading to patients with rare disorders feeling misunderstood.  Need for increased awareness and social support: The findings highlight the importance of increased awareness about rare diseases among outsiders, who may lack understanding and make judgments. Individuals with rare diseases often have to explain to others what the diagnosis is, why they are experiencing symptoms, or why they are not able to participate. |
| Carpenter  2011  USA [[11]](https://paperpile.com/c/jHvZKb/d8RqX) | To describe persons with vasculitis` most frequently used medication information sources, determine which sources patients perceive as credible, and explore gender differences in source use and perceived credibility | Quantitative: cross-sectional online survey | Medication information sources | Preferred sources of medication information: Patients predominantly relied on physicians and the Internet to obtain medication information, and rated them as the most credible sources.  Gender differences in information sources: Male patients used their spouse/partner more often and rated them as more credible than female patients did. Female patients were more likely to use medication package inserts and the Internet and were less likely to use nurses than male patients. Because male patients view their spouse/partner as a credible information source, providers may want to involve the spouse/partner in prescription decision-making.  Comparisons with other patient populations: The report suggests that there are both similarities and differences in the information-seeking behaviours of vasculitis patients compared to other patient populations. |
| Chaleat-Valayer  2019  France [[12]](https://paperpile.com/c/jHvZKb/ah01q) | To describe a therapeutic education program for patients with hypermobile Ehlers-Danlos syndrome and evaluate feasibility and effect on satisfaction and disease management in daily life | Quantitative: prospective observational study | Knowing and understanding the symptoms of the disease and the genetic transmission of the disease  Knowing and better anticipating management of the disease  Be able to exchange and sharing of experience  Knowing and managing drug treatment in case of crisis and daily medication  Knowing own`s rights | The intervention demonstrated success in multiple areas, including terms of recognition, satisfaction, increase of knowledge and adaptive skills. |
| Chaudhry  2013  USA [[13]](https://paperpile.com/c/jHvZKb/YrUf8) | To evaluate the experiences and opinions of patients in an adult Cystic Fibrosis center who went through a formal transition versus those who did not, in an attempt to evaluate the overall process and to identify means for improvement | Quantitative: cross-sectional survey | Understanding of the transition process from pediatric to adult programs  Navigating and coordinating health care | The formal transition process and patient satisfaction: On average, patients who participated in a formal transition process had higher satisfaction with both Cystic Fibrosis programs, perceived health status, and independence.  Anxiety levels and transition to the adult program: There were no significant differences in the level of anxiety between those who participated in a formal transition program, and those who did not.  Patient involvement in care transfer: The patients' opinions regarding the timing of care transfer were considered more often in patients who participated in a transition program. |
| Coathup  2016  Japan [[14]](https://paperpile.com/c/jHvZKb/eAYez) | To understand patients’ views and attitudes to using digital tools in patient registries and engagement with medical research in Japan | Quantitative: cross-sectional survey | Current satisfaction with information from clinicians and researchers  Current use of electronic devices  Acceptability of using electronic devices to engage with healthcare professionals and researchers | Information needs: The majority of patients felt that they were not receiving the information they wanted from their clinicians, which included recent medical research findings and opportunities to participate in clinical trials.  Engaging with digital technologies: 88 % of patients indicated they would be willing to engage with digital technologies to receive relevant medical information.  Data control: Patients also expressed an interest in having control over when and how they received this information, as well as being informed of how their data is used and shared with other researchers. |
| David  2015  France [[15]](https://paperpile.com/c/jHvZKb/gGqth) | To elucidate the education needs of patients who have undergone lung  transplant for cystic fibrosis while participating in the development of therapeutic education programs in French transplant centers | Multi method: qualitative interviews analyzed with descriptive statistics and quotations from interviewees | Knowledge about the disease and treatments  Experience of the disease | Patient knowledge and self-management: The patients had acquired much usable knowledge about managing their disease.  Educational needs on specific topics: Educational needs regarding transplant-related complications and management of immunosuppressive drugs were evident.  Additional support requirements: The analysis also demonstrated the need to better inform patients about pregnancy and new social rights and to offer them psychological support in adjusting to their new health status. |
| De la Corte-Rodriguez, 2019  Spain [[16]](https://paperpile.com/c/jHvZKb/xbkJH) | To evaluate the degree of education and empowerment of the patients of the Haemophilia Unit | Quantitative: cross-sectional survey | Knowledge about the disease  Making health decisions | Adequate knowledge about the disease: The results of the report indicate that, overall, patients with haemophilia possess ample knowledge about their disease.  Positive perception of institutional support: More than half of the patient sample considered that they benefited from adequate institutional support to help them control the factors influencing their health.  Patient empowerment in decision-making: The patients felt capable of making decisions on matters affecting their health. |
| Delisle  2016  Canada [[17]](https://paperpile.com/c/jHvZKb/FFTpG) | To identify reasons why people with systemic sclerosis do not participate in systemic sclerosis support groups | Quantitative: cross-sectional survey | Limitations of accessability and effectiveness of peer-led support groups | Key reasons for not participating in support groups included:  Not interested or no perceived need (36%)  No local support group available (35%)  Lack of awareness of support groups existence (13%)  Practical barriers (6%)  Emotional factors (4%)  Uncertainty about whether to attend (4%)  Negative perceptions about support groups (3%) |
| Dellon  2018  USA [[18]](https://paperpile.com/c/jHvZKb/5cSj6) | To: 1) explore knowledge and perceptions of palliative care among patients with CF, caregivers, and CF care providers; 2) solicit opinions about incorporating palliative care into routine CF care; and 3) solicit recommendations for CF-specific palliative care education for patients and caregivers | Qualitative: semi-structured interviews analyzed using content analysis | Knowledge about palliative care | Knowledge gap in palliative care: Many patients with Cystic Fibrosis, caregivers, and providers lacked knowledge about palliative care.  Positive perception of palliative care: After learning more about palliative care, patients, caregivers and providers thought it could be helpful, and that it should be introduced earlier. |
| Depping  2021  Germany [[19]](https://paperpile.com/c/jHvZKb/yQdqR) | To evaluate the efficacy of a brief, transdiagnostic, peer-delivered intervention for patients with rare diseases in addition to care as usual compared with care as usual only | Quantitative: randomized clinical trial with two arms and measures at baseline, 6 weeks, and 6 months | Social support  Impact of health education  Illness perception and cognition | Increased acceptance of the disease: The intervention group had significantly higher rates of acceptance of the disease compared with the control group.  Improved secondary outcomes: Several secondary outcomes, including different coping strategies, social support, and mental quality of life, were significantly higher after the intervention compared with the control group. |
| Dicianno  2016  USA [[20]](https://paperpile.com/c/jHvZKb/aUXam) | To determine whether an evidence-based wellness program would improve health outcomes and patient experience of care, result in a positive return on investment and provide evidence for scalability | Quantitative: non-randomized non-controlled cohort study with measures at baseline, 6, 12, 18, and 24 months | Patients experience of care | Long-term improvements: This report founds that significant improvements were observed in all main outcome measures were seen after two years of enrolment.  Return on investment: Although the costs in year one of enrolment increased because of hospitalizations and the overall return on investment was negative, a small positive return on investment was seen in year two of enrolment. |
| Domaradzki  2016  Poland [[21]](https://paperpile.com/c/jHvZKb/DIUcI) | To explore the perception of healthcare services of family caregivers | Qualitative: interviews analyzed with content and thematic analysis | Family caregivers` experiences with healthcare services | Lack of knowledge from physicians: Caregivers were frustrated by the lack of knowledge about the diagnosis from physicians.  Lack of empathy and support: Caregivers reported that physicians did not show enough empathy and support towards them.  Lack of trust in the healthcare system: Caregivers felt let down by the system and expressed a general lack of trust in the quality of healthcare. |
| Dwyer  2014  Switzerland [[22]](https://paperpile.com/c/jHvZKb/Vr9L8) | To: 1) determine if web-based platforms could be effectively used to conduct an online needs assessment; 2) identify the unmet health and informational needs and; 3) assess patient acceptability regarding patient-centred, web-based interventions to bridge shortfalls in care | Mixed methods: cross-sectional online survey, followed by focus groups analyzed with thematic analysis | Health and informational needs  Patient acceptability of web-based interventions to bridge shortfalls in care | Online information seeking: The respondents of the survey were active internet users, nearly all had sought information about their diagnoses online.  Reliance on online support: The survey respondents rated the internet, healthcare providers, and online community as equally important information sources on their diagnosis.  Positivity to online support: Focus group participants were overwhelmingly positive regarding online interventions/support with links to reach expert healthcare providers and peer-to-peer support. |
| Etchegary  2011  Canada [[23]](https://paperpile.com/c/jHvZKb/SGJDM) | To explore the healthcare experiences of families, and elicit their suggestions for improvement in the quality of care provided to them | Qualitative: interviews analyzed with qualitative description | Family members experience with healthcare | Frustration concerning lack of knowledge: Participants expressed frustration at the lack of knowledge about the diagnosis displayed by their family physicians.  Difficulties in accessing healthcare and support: Participants described numerous difficulties accessing appropriate healthcare and other supports, and anticipated access difficulties in the future.  Suggestions for improvement of care: Participants offered several suggestions to improve the quality of care to their families, including better education of healthcare professionals about the complex nature of the disease and the provision of regular follow-up support. |
| Flewelling  2019  USA [[24]](https://paperpile.com/c/jHvZKb/2nE7M) | Explore the relationship between social support, mental health, physical health, treatment activity, and disease-specific quality of life | Quantitative: cross-sectional survey | Social support | Impact of social support on health outcomes: Greater social support was associated with fewer self-reported mental and physical health symptoms, digestive symptoms, and eating disturbances over time.  Benefits of social support on functioning and body image: Social support also was associated with elevated emotional, social, and role functioning as well as vitality and improved body image.  Perception of treatment burden and health: Those who reported more support perceived less treatment burden and better overall perceptions of their health. |
| Foley  2014  Ireland [[25]](https://paperpile.com/c/jHvZKb/MTsUv) | Identify key psycho-social processes that underpin how people with motor neuron disease engage with healthcare services | Qualitative: interviews analyzed using a grounded theory approach | Understanding and use of healthcare services | Key variables that shape how people with motor neurone disease engage with healthcare services:  Control in care to cope with loss  Reassurance about end-of-life care  Questioned the benefit of life-sustaining interventions in palliative care |
| Garrino  2015  Italia [[26]](https://paperpile.com/c/jHvZKb/USEvG) | To explore the experiences of illness of patients with rare diseases and the health professionals | Qualitative: interviews analyzed with a phenomenological approach | Dealing with disease development  Living with the disease  Everyday living  Relating to others  Relations to professional healthcare providers | Disease management challenges: Dealing with disease development, getting a diagnosis, treatment approach, and seesaw progression between remission and relapse. Living with the disease, adaptation strategies, the quest for independence, the need to “know the disease.  Impact on daily life: Challenges in everyday living; functional limitations, experiencing pain, influence on work.  Support and relationships: Challenges in relating to others; the role of the family, and comparisons with other patients. Relations to professional healthcare providers; admission and follow-up, features of the relationship, the need to receive information. |
| Gumuchian  2018  Canada [[27]](https://paperpile.com/c/jHvZKb/eUS6t) | To gain a greater understanding of the coping strategies  employed by people living with scleroderma | Qualitative: focus groups analyzed with content analysis | Knowledge of coping strategies  Access to support services | Coping strategies: Participants reported using a combination of problem-focused (e.g., professional help; seeking disease-related information), emotion-focused (e.g., social support; adaptive distraction techniques), and meaning-focused coping strategies (e.g., benefit finding; goal reappraisal) to help them to cope with and manage their disease.  Challenges in accessing support services: Many of the patients reported having difficulty accessing support services. |
| Hiermeier  2020  UK [[28]](https://paperpile.com/c/jHvZKb/N9M0G) | To determine the views of Duchenne Muscular Dystrophy patients and their caregivers about discussing the risk of sudden death and their acceptance of implantable cardiac defibrillators | Qualitative: focus groups analyzed with thematic analysis | Access to quality of information provided by professionals and patients who had already received implantable cardiac defibrillators  Decision-making about implantable cardiac defibrillators  Individuals` own lived experience | Discussion of risks: Patients wanted to have their risk of sudden arrhythmic death discussed, when relevant.  Individualized discussions: If implantable cardiac defibrillators therapy were established as beneficial, they would welcome an individualized discussion about its appropriateness for them. |
| Hoefnagels  2020  The Netherlands [[29]](https://paperpile.com/c/jHvZKb/Drrpi) | To test the feasibility and effects of two interventions focused on acceptance and self-management | Quantitative: feasibility study of two tailored interventions (face-to-face and online) with no control group | Acceptance and self-management of disease | Feasible face-to-face interventions: The face-to-face intervention was evaluated as feasible; the preliminary results were promising as adherence, quality of life and illness perception all improved.  Challenges with online interventions: The online intervention was evaluated as not feasible because of difficulties with the enrolment, recruitment, and retention. |
| Ioannou  2010  Australia [[30]](https://paperpile.com/c/jHvZKb/FbPsE) | To explore reasons  for having screening, knowledge of cystic fibrosis, recollection, understanding and impact of carrier status and communication of results to family members | Quantitative: cross-sectional survey | Knowledge, recollection and meaning of carrier status | Knowledge and carriers: Carriers answered the knowledge questions correctly more often than non-carriers.  Anxiety levels: There was no difference in anxiety between carriers and non-carriers.  Information sharing: The majority of carriers informed relatives of their increased risk of being a carrier. Participants’ attitude towards carrier screening for cystic fibrosis was generally very positive. |
| Jackson  2020  Irland [[31]](https://paperpile.com/c/jHvZKb/A1Lks) | To examine associations between health literacy and cystic fibrosis outcomes | Quantitative: a cross-sectional survey of a registry cohort study with an age/sex matched comparison group from the general population | Health literacy describes an intermediate outcome of health education and health promotion and refers to an individual’s capacities, skills and motivation to make judgements and decisions in everyday life concerning healthcare, disease prevention and health promotion  Interactive health literacy is examined in this study, as it encompasses the cognitive, literacy and social skills that enable individuals to access, understand, appraise and apply different forms of health information to changing circumstances  It describes e.g. the ability to find information on symptoms that are concerning, understand a doctor/pharmacist’s instruction on taking prescribed medication, judge how information from your doctor applies to you and the ability to follow the instructions on medication. It also reflects dimensions of patient empowerment, such as perceived meaning and importance of health information, perceived competence to perform healthy behaviours and self-determination | Health literacy and healthcare utilization: Sufficient health literacy was associated with fewer outpatient visits, days hospitalized, days of intravenous antibiotics and reduced healthcare resource utilization in the 12 months preceding the assessment of health literacy.  Health literacy and quality of life: Participants with sufficient health literacy had a significantly better quality of life compared to those with lower health literacy.  Health literacy and patient involvement: better health literacy is correlated with greater patient involvement, in decision-making, patient knowledge, sense of responsibility and patient empowerment. |
| Katavic  2016  Croatia [[32]](https://paperpile.com/c/jHvZKb/RJ302) | Find out what aspects of information behaviour are associated with more and less positive beliefs and feelings about rare diseases | Quantitative: cross-sectional survey | Health information behaviour | Health information seeking and concern about illness: Health information seeking was associated with more concern about the illness, but was not associated with personal control over the illness or patients’ ability to understand the illness.  Information sources and understanding of illness: Using patient organizations as an information source was associated with a better understanding of the illness.  Avoiding health information: Participants who avoided health information had less personal control over the illness and less ability to understand the illness, but no association was found with concern about the illness. |
| Katavic  2019  Croatia [[33]](https://paperpile.com/c/jHvZKb/t7KX4) | To describe distinctive aspects of health information behaviour of rare disease patients and specific challenges they face when seeking health information | Qualitative: interviews analyzed with thematic analysis | Health information behaviour (seeking, finding and sharing) | Information seeking and sharing: Health information behaviour of rare disease patients are characterised by independent and continuous health information seeking and sharing.  Importance of connecting with other patients: Connecting with other patients and getting realistic insight into the condition after diagnosis, advice for everyday life, comfort and hope and confirmation that their symptoms are ‘normal’ is of particular importance.  Challenges in support: Lack of specific advice for daily life, inaccessible new knowledge, lack of information about drugs and encountering severe health information are common challenges patients face due to insufficient support from health care professionals. |
| Kazmerski  2017  USA [[34]](https://paperpile.com/c/jHvZKb/gW49Y) | To explore attitudes and decision-making regarding pregnancy | Qualitative: interviews analyzed with thematic analysis | Understanding of the effect of diagnosis on fertility and pregnancy  Shared decision-making  Ability to actively engage with health care providers | Important factor in decision-making: Participants indicated cystic fibrosis as a major factor in pregnancy decision-making.  Lack of clarity: Although women acknowledged that cystic fibrosis influences attitudes toward pregnancy, many expressed confusion about how cystic fibrosis can affect fertility/pregnancy.  Perceived disapproval: Many perceived disapproval from cystic fibrosis providers regarding pregnancy and were dissatisfied with reproductive care. |
| Kesselheim  2015  USA [[35]](https://paperpile.com/c/jHvZKb/sghIR) | To explore rare disease patients’, caregivers’, and advocates’ experiences with their conditions and the health care system, in addition to their perspectives on drug development | Qualitative: focus groups analyzed for common themes | Learn to live with a poorly understood condition  Seek and find information and care  Ability to actively engage with health care providers | Living with a poorly understood condition: Participants described the challenges of learning to live with a poorly understood condition for which treatment is limited.  Willingness to accept risks and frustrations with the costs of care: Rare disease patients were willing to accept certain risks in their care in the hopes of finding some benefit, but also expressed frustrations with the costs of their care and the lack of scientific data about their treatments.  A desire for effective treatments and improved quality of life: Participants were concerned that the development and testing of therapies should, as quickly as possible, yield effective treatments to advance their quality of life. |
| Keyte  2020  UK [[36]](https://paperpile.com/c/jHvZKb/gQqvi) | To explore beliefs about healthy risk behaviours | Qualitative: interviews analyzed using thematic analysis | Knowledge about consequences of healthy risk behaviours | A desire for normalcy and engagement in risky behaviors: A desire for normalcy was evident, often accompanied by engagement in everyday health risky behaviours as a method of minimising the illness identity.  Life-oriented illness perspective: Evidence of a life-orientated illness perspective was also prevalent, with participants engaging in some risky behaviours for fun.  Lack of knowledge on the consequences of risky behaviours: Overall, there was a lack of knowledge on the consequences of healthy risk behaviours, with many participants reporting not being informed of these by clinicians. |
| Kirk  2016  UK [[37]](https://paperpile.com/c/jHvZKb/7Ed62) | To explore how online peer support is used by young people and parents to support self-care in relation to cystic fibrosis | Qualitative: online ethnography observing, downloading and analyzing discussion group postings | Online social peer support | Role of online support groups: Online support groups appear to supplement professional support in relation to self-management.  Sharing experiences: Online support forums are suitable platforms to share experiences.  Information and support seeking: Parents sought information and support on managing specific therapies/services and ways of maintaining their child’s health, the information and support. Young people's desires appeared to be more directed at how to ‘fit’ cystic fibrosis into their everyday lives. |
| Kurtz 2019  Mexico [[38]](https://paperpile.com/c/jHvZKb/NyU4c) | Document disease burden related to oculopharyngeal muscular dystrophy from the patient’s perspective | Qualitative: interviews analyzed using the framework technique | Access to disease information  Knowledge about coping strategies | Coping strategies and limited access to disease information: Two emergent themes were coping strategies and limited access to disease information, particularly concerning dysphagia.  Important aspects of the condition: Pelvic girdle weakness, ptosis, difficulty speaking, pain, shoulder girdle weakness and fatigue were also important aspects.  Focus on impairments versus the complete disease burden: The traditional medical perspective, including medical research, mainly focuses on patients' impairments, rather than on the whole disease burden, which includes coping strategies. |
| Laberge  2010  Canada [[39]](https://paperpile.com/c/jHvZKb/WgRgD) | To measure the clinical and genetic knowledge of Myotonic Dystrophy type 1 patients and compare it to Myotonic Dystrophy type 1 noncarriers | Quantitative: cross-sectional study of persons with diagnose, compared to clinically normal noncarriers and controls | Genetic knowledge | Genetic knowledge and education level: Myotonic dystrophy type 1 patients’ genetic knowledge is significantly dependent of the level of education and the number of CTG repeats. |
| LaDonna  2015  Canada [[40]](https://paperpile.com/c/jHvZKb/QtFKC) | To conduct an in-depth exploration of participants’ understanding about myotonic dystrophy 1 and to identify knowledge gaps that may challenge patient-centred care provision | Qualitative: photovoice and interviews | Health literacy is defined as “the degree to which individuals have the ability to obtain, process, and understand basic health information and services needed to make appropriate decisions” | Good core knowledge: Findings showed that participants had good core knowledge with respect to their disease and its implications.  Fragments of misinformation: However, each participant held fragments of misinformation that influenced their decision-making process.  Need for further knowledge and education: The report emphasized the importance of increased education and awareness about symptoms, genetic information and treatment strategies for patients, their family members, and healthcare providers. |
| Le Doré  2021  France [[41]](https://paperpile.com/c/jHvZKb/wMyim) | To evaluate the impact of a hemarthrosis-simulating artificial knee | Quantitative: observational study with three-time points – inclusion, at hemarthrosis-simulating artificial knee and after 6 months after hemarthrosis-simulating artificial knee | Knowledge about diagnose  Knowledge about management | Benefits from individualized training courses: An individualized training course can enhance the understanding of haemophilia in patients of all ages, especially in children and teenagers.  Improving disease management with a hemarthrosis-simulating artificial knee: The hemarthrosis-simulating artificial knee may assist in improving patients’ management of their disease, by simulating joint bleeding. |
| Lewis  2016  USA [[42]](https://paperpile.com/c/jHvZKb/OWQ6v) | The goal of this project was to assess  the current level of medication-related knowledge and independence with self-care skills in patients with cystic fibrosis | Quantitative: cross-sectional survey | Knowledge about medication and self-care skills | Age and medication-related knowledge: Scores on medication-related knowledge and skills generally increased with increasing age.  Deficiencies in medication-related self-care skills among young adults: Many young adults were not able to complete many essential medication-related self-care skills. |
| Lindsay  2016  Canada [[43]](https://paperpile.com/c/jHvZKb/k1qQm) | To explore youth and parent experiences of a new transition model for youth with spina bifida, compared to the experiences of young adults with spina bifida who did not participate in the model | Qualitative: interviews analyzed with thematic analysis | Information and knowledge to promote the development of self-care, self-advocacy, and life skills in the transition from youth to adult health care | Helpful transition model: The new transition model for youth with spina bifida can help enhance participants’ transition experiences and preparation for adulthood. |
| Lindvall  2010  Sweden, Denmark and Norway [[44]](https://paperpile.com/c/jHvZKb/TLAot) | To investigate patient`s knowledge of their disease | Quantitative: cross-sectional survey | Knowledge of disease | Knowledge based on severity: This study has shown that the level of knowledge about haemophilia and its treatment are lowest among patients with mild haemophilia.  Specific knowledge gaps in more severe cases: Patients with severe and moderate diseases have a lack of knowledge in specific areas. |
| Litzkendorf 2020  Germany [[45]](https://paperpile.com/c/jHvZKb/qtT9o) | To examine the use of information sources for different rare diseases, how they rate and use information sources | Qualitative: interviews analyzed with content analysis | Use of information sources | A variety of important information sources: Various information sources, such as the internet, self-help organizations, and doctors, have been confirmed as important access channels for people living with a rare disease and their families. |
| Lonabaugh  2018  USA [[46]](https://paperpile.com/c/jHvZKb/MJZh5) | To improve patient education in clinic by evaluating patient perceptions of education provided, and patient self-confidence related to cystic fibrosis | Quantitative: cross-sectional survey | Knowledge about diagnosis | Lacking information on mental health: Participants reported frequent education on all topics except for reproductive effects of cystic fibrosis and mental health.  Correlation between confidence and knowledge in adolescent caregivers: There was a statistically significant correlation in overall confidence in cystic fibrosis-related knowledge and performance on a validated knowledge scale for adolescent caregivers, but not for the other groups. |
| Mälstam  2018  Sweden [[47]](https://paperpile.com/c/jHvZKb/Ub991) | To generate knowledge about challenges and possibilities situated in everyday life, by fully engaging persons with autoimmune Addison`s disease in the research process as experts | Qualitative: photovoice and focus groups analyzed using thematic analysis | Knowledge about the diagnosis  Knowledge about coping-strategies | Five themes emerged: individual and fine-tuning in everyday life; it is not how it was; the power of knowledge and support; becoming the expert in an uncertain context; and finding balance and paving new ways.  Complex everyday life: The findings showed that everyday life with autoimmune Addison`s disease was more complex than earlier portrayed; entailing several barriers and negotiations. |
| Merker  2018  USA [[48]](https://paperpile.com/c/jHvZKb/F0MxJ) | The purpose of this study was to comprehensively describe health literacy | Quantitative: cross-sectional electronic survey | Definition of Health literacy—“the degree to which individuals have the capacity to obtain, process, and understand basic health information and services needed to make appropriate health decisions” | Impact of patient characteristics on health literacy: Patients with neurofibromatosis, those with lower education and those with learning disabilities had lower scores on health literacy.  Predictors of health literacy: In multivariate analysis, learning disability and education remained significant predictors of health literacy scores.  Association with adapted critical health literacy: Lower education was associated with lower adapted critical health literacy scores. |
| Mohan  2020  India [[49]](https://paperpile.com/c/jHvZKb/SR6SC) | To explore the status of haemophilia and knowledge, attitude and behaviour of haemophilia patients towards their condition | Quantitative: cross-sectional survey | Access to fundamental knowledge (general) about the diagnoses  Access to technical knowledge (symptoms etc)  Access to knowledge about management  Access to treatment | Impact of haemophilia: Most respondents suffered from severe haemophilia and co-morbidities such as anxiety, stress, chronic pain and headache. All of them felt that haemophilia interfered with living a normal life and perceived a grim future.  Support needed for young people in India: Young people in India need technical, financial and psychological support to prevent complications related to haemophilia.  Behavioural changes for improved quality of life: While most of them take responsibility for their health, more behavioural changes need to be introduced to improve their quality of life. |
| Molster  2016  Australia [[50]](https://paperpile.com/c/jHvZKb/MJmjI) | To explore the experiences of Australian adults living with rare diseases in relation to diagnosis, information provision at the time of diagnosis, use of health and support services and involvement in research on their condition | Quantitative: cross-sectional online survey | Diagnostic information at the time at the diagnosis  Support in transition from pediatric to adult services  Timely post-diagnosis information  Access to health-professionals that are aware and acknowledgeable about the diagnoses  Follow-up and the quality of contact with health- and support services | These findings suggest that some crucial healthcare needs of people living with rare diseases are being unmet:  30.0 % waited five or more years for a diagnosis.  66.2 % had seen three or more doctors to get a diagnosis.  45.9 % had received at least one incorrect diagnosis.  Almost three quarters (72.1 %) received no or not enough information at the time of diagnosis.  15.4 % of respondents had ever used paediatric services, 52.8 % of these had experienced problems in the transition from paediatric to adult services.  20.3 % knew of a patient registry for their condition and 24.8 % were informed of clinical trials. |
| Mooney  2013  UK [[51]](https://paperpile.com/c/jHvZKb/FI2wI) | To explore the informational needs of patients with anti-neutrophil cytoplasmic antibody-associated vasculitis | Qualitative: focus groups and individual interviews analyzed using the Framework technique | Access to information to knowledgeable health- practitioners  Possibilities to discuss diseases and management  Access to written information | Reaction to diagnosis: When given the initial diagnosis, all patients described themselves as being too ill to take in information and that they later found it difficult to find information.  Need for written information on disease management: Most information received at diagnosis was in the form of verbal information given by the hospital doctor. Patients wanted access to knowledgeable practitioners and positive but truthful information, in the form of a booklet. |
| Mulders  2012  The Netherlands [[52]](https://paperpile.com/c/jHvZKb/waPPC) | To investigate whether or not an educational e-learning program improves the knowledge and skills of  adult patients with haemophilia on home treatment | Quantitative: randomized controlled trial with measures at baseline and one month | Knowledge of treatment and complications as well as practical skills | Improving knowledge and skills through e-learning: In patients with haemophilia, who are on home treatment, knowledge of haemophilia treatment and complications as well as practical skills can be improved by an educational e-learning program. |
| Naik  2019  USA [[53]](https://paperpile.com/c/jHvZKb/I8YSJ) | To explore psychosocial issues | Qualitative: focus groups analyzed with thematic analysis | Importance of real diagnosis  Knowledge about symptoms and progression of the disease  Knowledge about how to manage the disease  Social support by meeting others with the same diagnosis | Reduced isolation: young adults felt less isolated by being given a real diagnosis.  Coping with the diagnosis: To think of the diagnosis as an “invisible disability” helped them cope with the disease and how they manage it, as well as how they explained it to others.  Knowledge gaps and peer support: young adults expressed embarrassment over having to explain the disease to others. The young adults felt that overall, they knew how to control their disease, but did not have a good understanding of the pathophysiology of the disease and how it causes phototoxicity symptoms and affects the liver. Meeting/talking to others with the same diagnosis was also useful to share tips on how to manage symptoms, and how to talk about it with others. |
| O`Mahar  2010  USA [[54]](https://paperpile.com/c/jHvZKb/9Xbav) | To design and evaluate a camp-based intervention, the goal of which was to increase independence among children, adolescents, and adults with spina bifida | Quantitative: one-group pretest-posttest | Knowledge about self-care and management of the disease | Significant improvement in goals, responsibilities, and independence: Adults with spina bifida made significant gains in individual goals, management of spina bifida responsibilities, and independence with general spina bifida tasks, with medium effect sizes observed in goal attainment. |
| Pakhale  2016  Canada [[55]](https://paperpile.com/c/jHvZKb/JbQBZ) | To examine how well adults living  with Cystic Fibrosis understand the treatment recommendations and how this information may play a role in  impacting self-reported adherence to therapy | Quantitative: observational cohort study | Knowledge about treatment recommendations  Health professionals must use effective communication strategies | Misunderstanding treatment recommendations: A significant number of adults misunderstand treatment recommendations and this likely plays an important role in treatment adherence.  Overall correct understanding: At least 70% of participants were correct in understanding whether a treatment was recommended to them.  Significant variation in recommendation frequency understanding: Participants’ understanding of the recommendation frequency varied widely across all treatments (12.2–100%). |
| Parvizi  2017  Iran [[56]](https://paperpile.com/c/jHvZKb/xPYI4) | To assess health literacy in patients with epidermolysis bullosa in Iran | Quantitative: cross-sectional survey | Health literacy defined as “the patients’ skills on reading, listening, analysing decisions making and applying these skills to the situation related to health monitoring and coordination for strategy plan in term of health promotion” | Low health literacy level: This report revealed a low health literacy level (57.6%) and inadequate health literacy level (15.2%) among the participants.  Variations based on disease type and geographic region: There were variations in health literacy level according to the type of disease and the geographic region. |
| Raphaelis  2018  Switzerland and Austria [[57]](https://paperpile.com/c/jHvZKb/ZBcTR) | To determine whether written information and/or counselling decreases illness-related uncertainty in women with vulvar neoplasia | Quantitative: a multicenter randomized study with two groups with measures at baseline and six months | Information regarding disease treatment, common symptoms, assessment and management. | Improvements in the counselling group, but not in the written information-group: The counselling group experienced significant improvements over time, within the subscales of ambiguity, inconsistency, and unpredictability. No such improvement was seen within the written information group.  Counseling's effectiveness in addressing specific uncertainties: In addition, counselling improved inconsistency over time, and total uncertainty, inconsistency, and unpredictability at distinct time points more efficiently than written information. |
| Riklin,  2017  USA [[58]](https://paperpile.com/c/jHvZKb/MRsMG) | To test the relationship of psychosocial characteristics, clinical-demographic variables, and health literacy to  patient satisfaction in adults with neurofibromatosis 1 | Quantitative: cross-sectional survey | Health literacy is defined as the «capacity of individuals to access, understand, and use health information to make informed and appropriate health-related decisions» | Psychosocial functioning improvement: The participants report better psychosocial functioning. However, no increase in physical functioning was seen.  Association between health literacy and satisfaction: Better overall functional, communicative, and critical health literacy was significantly associated with higher satisfaction with the medical visit. |
| Ringqvist  2021  Sweden [[59]](https://paperpile.com/c/jHvZKb/bQoRK) | To determine whether the psychological benefits of intense, inpatient, multimodal rehabilitation for persons with Huntington’s disease, as found in earlier reports, also apply in a shorter, daycare setting | Quantitative: prospective, non-randomized, non-controlled cohort with measures at baseline and after 8 weeks | Intervention with focus on making life more understandable, manageable and meaningful | Impact of multimodal day-care rehabilitation: The results of this study indicate that an 8-week multimodal day-care rehabilitation programme can be tolerable, reduce psychiatric symptoms (anxiety and depression) and improve health-related quality of life for people with Huntington’s disease.  Relationship between sense of coherence and attendance rate: A sense of coherence seems to be related to attendance rate, indicating that efforts to make life more understandable, manageable, and meaningful for people with Huntington’s disease might increase participation in treatments. |
| Rosnau K  2017  USA [[60]](https://paperpile.com/c/jHvZKb/uAQtI) | To investigate the association between knowledge of neurofibromatosis 1 and self-esteem | Quantitative: cross-sectional online survey | Access to knowledge about the diagnoses.  Access to support groups and friends  Receiving genetic counselling  Increased self-esteem | High degree of knowledge of the diagnose  No correlation between knowledge and self-esteem  Self-esteem was positively influenced by:  Having friends with neurofibromatosis  Being in a neurofibromatosis support group  Having received genetic counselling |
| Rovira-Moreno  2020  Spain [[61]](https://paperpile.com/c/jHvZKb/bOYFm) | To assess the overall impact of an educational training program | Quantitative: noncontrolled pre-post intervention study with three measurement times not specified | Knowledge about diagnose and management | Knowledge improvement and psychological impact management: The results show improvements in knowledge and better management of the psychological impact.  Non-significant decrease in anxiety: The mean score for anxiety decreased but was not statistically significant.  Positive evaluation and interest in advanced editions: Overall, the program was evaluated by the participants as a highly beneficial experience and all of them were interested in attending advanced editions |
| Salvatore  2018  USA [[62]](https://paperpile.com/c/jHvZKb/oj9o4) | To assess if quality of life can be improved by patients being connected to other patients (in diagnose-focused support organization) | Quantitative: retrospective cross-sectional survey | Social support - contact with peer support groups and/or other patients  Support from their physicians in having contact with peer support groups | Improved perceptions of overall health and well-being: Connected respondents showed significantly improved perceptions of overall health, disease severity, motivation to take care of health and emotional well-being.  Active connection leads to greater benefits: Any level of connection produced noticeable benefits, but the active connection in the form of regular interaction with other patients reported the greatest improvements.  Higher satisfaction with a primary treating physician: Respondents reported higher levels of satisfaction with their primary treating physician after being connected. Also, the majority of patients (62%) reported joining support groups following referrals from their physicians. |
| Shepherd 2017  UK [[63]](https://paperpile.com/c/jHvZKb/QTft0) | To explore patients’ primary adrenal insufficiency knowledge and understanding of the condition, steroid replacement adjustment during acute illness or stress and provided education | Mixed method: using qualitative interviews and hospital case note review analyzed with content analysis | Knowledge and understanding of the condition | Good level of knowledge but poor application: All participants had a good level of knowledge and understanding of required medication however application in times of need was poor.  Importance of support from family and health professionals: Medication adherence and prevention of a crisis relied not only on patient knowledge and application but also the support of family and health professionals. |
| Shoshan  2012  Israel [[64]](https://paperpile.com/c/jHvZKb/c2HkT) | To describe the basic knowledge of body anatomy and functioning, and the sexual knowledge of young adults with spina bifida and to investigate  the associations between independence in daily functions and communication skills on the one hand, and sexual knowledge and activity on the other | Mixed method: cross-sectional survey followed by individual interviews- analyses method not described? | Knowledge of body anatomy and functioning  Sexual knowledge  Communication skills | Limited knowledge of body anatomy and sexual information: All of the participants were exposed to some form of sex education, but their actual knowledge of body anatomy and functions and sexual knowledge was found lacking.  Age-related differences in sexual knowledge: The sexual knowledge, motor and functional abilities were statistically significantly better in participants aged above 20, compared to participants aged under 20. |
| Skirton  2010  USA [[65]](https://paperpile.com/c/jHvZKb/wuwkK) | To explore the perceptions of family caregivers regarding the availability and adequacy of health and social care services for their family member with Huntington disease | Quantitative: cross-sectional survey | Availability and adequacy of health and social services  Community resources  Knowledge about the diagnose | Factors influencing caregiving in Huntington disease: Three main factors were derived: ‘community resources’, ‘individualized care’ and ‘knowledge of Huntington disease’.  Concerns about healthcare professionals' knowledge and available services: Carers had concerns about the knowledge of healthcare professionals providing care and thought that there were insufficient services to support them and the affected person.  Challenges faced by carers with long-term neurodegenerative conditions: There were different challenges for carers when the affected person had a long-term neurodegenerative condition because these carers were also likely to have responsibilities for earning and caring for children. |
| Smedley 2021  UK [[66]](https://paperpile.com/c/jHvZKb/iKCP3) | To investigate how the  process of genetic testing is discussed within Huntington disease forums | Quantitative: cross-sectional survey | Information about genetic testing  Decision-making  Social support | Themes in discussions surrounding genetic testing: Discussions examined three themes: deciding to be tested (enquiring about symptoms and starting a new family), preparing for the test (information seeking and attending appointments) and receiving the results.  Role of forums in genetic testing Forums can reduce the uncertainty of ambiguous symptoms and provide ongoing personalised support before, during and after a genetic test. |
| Smolich  US  2020 [[67]](https://paperpile.com/c/jHvZKb/w6EQp) | To determine the effectiveness of that booklet in improving fragile X premutation-related health knowledge among women with a fragile X premutation | Quantitative: noncontrolled pre-post study with one group and measures at baseline and after 3 months | Written targeted education information about the disease | Impact of booklet on health knowledge: Health knowledge scores were significantly increased after receipt of the booklet.  Participants answered that the booklet was ‘very helpful’ (44.6%) or ‘somewhat helpful’ (38.5%).  Usage of the booklet: Twenty-four participants (34.8%) reported using the booklet to explain concepts to family members.  Gaps in knowledge: Although we found that the booklet provided women with needed information, we found that gaps in knowledge still exist. |
| Socha Hernandez  2020  Australia [[68]](https://paperpile.com/c/jHvZKb/ak9YJ) | To investigate the experience of Australian individuals with Charcot-Marie-Tooth disease in using medications, including perceived impact of drug-induced adverse effects;  and to determine whether individuals with Charcot-Marie-Tooth disease feel adequately supported to make decisions about medication safety | Qualitative: focus groups and individual interviews, analyzed with thematic analysis | Obtaining safety information about medication  Enough attention and good information from professionals so patients could make informed decisions | Sources of medication information: Participants sought medication information primarily from general practitioners or neurologists.  Barriers to medication information: The main barriers identified by participants were a perceived poor understanding in non-specialist health professionals about Charcot-Marie-Tooth disease and a lack of attention to medication safety concerns in people with Charcot-Marie-Tooth disease; this resulted in dissatisfaction with the advice provided.  Alternative information sources: Many individuals who faced uncertainty in obtaining and understanding medicines information turned to Internet resources, peer groups, and the use of complementary and alternative medicines to self-manage Charcot-Marie-Tooth exacerbations. |
| Stubberud  2015  Norway [[69]](https://paperpile.com/c/jHvZKb/p63HC) | To determine the efficacy of goal management training on aspects of perceived emotional health and coping in individuals with Spina Bifida | Quantitative: randomized controlled trial with a wait-list control group and measures at baseline, postintervention and after 6 months | Knowledge about how manage the disease | Effects of goal management training: Findings indicated positive effects of goal management training relative to the control group on measures of emotional health. The intervention group showed significant improvement, compared with control subjects, on a self-report inventory of depressive and anxiety symptoms after training, lasting at least 6 months posttreatment.  Improvements in mental health: Both the intervention group and the control group showed improvements in mental health components of health-related quality of life after training.  Coping strategies: The intervention group showed a significant increase in task-focused coping and a decrease in avoidant coping after training. |
| Sylvain  2016  Canada [[70]](https://paperpile.com/c/jHvZKb/Mxyfc) | To use Leventhal’s Common-Sense Model to explore patients’ representations of cystic fibrosis related diabetes to better understand the discrepancy between patients’ expected and observed health behaviours | Qualitative: interviews analyzed with Miles, Huberman and Saldanas method | Information about the diagnoses- specified related to the individual needs  Adapting to the situation | Information needs on cystic fibrosis-related diabetes: Patients require specific information on cystic fibrosis-related diabetes.  Importance of the screening phase: The screening phase could be a crucial time to help patients adjust their representations to fit the reality of cystic fibrosis-related diabetes. |
| Takeuchi  2016  Japan [[71]](https://paperpile.com/c/jHvZKb/KQQEk) | To describe the lives and illness of people with idiopathic basal ganglia calcification | Qualitative: interviews analyzed with unspecified qualitative methods | Access to disease information  Establishing peer and family support system | Worries and attitudes: The qualitative data clarified the patients’ worries and attitudes towards their lives, anxiety about clinical features, and worries about marriage and childbirth. Worries about disease changes, not receiving enough information about the diseases on the internet or books to understand the diagnoses.  Information gaps and dissatisfaction: The more correct information they received, the more satisfied the patients were. By receiving adequate knowledge, the patient's attitude towards the diagnosis was able to gradually become more positive.  Need for a genetic and mental support system: The findings emphasized that a support system addressing genetic and mental health aspects should be established. |
| Théaudin  2014  Frankrike [[72]](https://paperpile.com/c/jHvZKb/peNAk) | The purpose was through a qualitative study on patients` needs to construct a therapeutic patient education program | Qualitative: interviews analyzed with unspecified qualitative methods | Information and knowledge disease (symptoms, impact of genetic testing, impact of liver transplantation, impact of physical and mental health)  . | Knowledge of the disease: The participants had fairly good knowledge of the disease but had information gaps when it comes to all the symptoms associated with it.  Confusion about symptoms: The participants expressed confusion regarding symptoms related to disease or treatment.  Patient education program preferences: The participants expressed what specific themes they would like to be included in a patient education program: (1) symptoms of the disease; (2) liver transplant; (3) cardiac involvement and role of a pacemaker; (4) how to apply for social welfare; (5) treatment of the disease other than liver transplant, mechanisms of action; (6) management in the case of ongoing difficulties with medications; (7) post-liver transplant follow-up; (8) cardiac follow-up; (9) symptomatic medications. |
| Torres-Ortuño 2018  Spain [[73]](https://paperpile.com/c/jHvZKb/VD66O) | To see if disease behavior and disease perception together with coping strategies modulate adherence to treatment | Quantitative: cross-sectional survey | Understanding of patient’s perception, behaviour and attitude towards the disease to achieve a better adaptation of their coping resources | Adherence rate: 56% of the patients were found to be adherent to their prescribed treatment.  Age not a factor: No significant associations were found between age and adherence.  Quality of life impact: Poor adherence was associated with a lower quality of life. |
| Van Balen 2018  Canada [[50,74]](https://paperpile.com/c/jHvZKb/MJmjI+BfIZ7) | To explore how visual information on an individual’s pharmacokinetic profile and bleed history, and encouragement in treatment decisions could change the patients understanding of their disease | Qualitative: interviews analysed with content analysis | Communication and information about the disease | Helpful approach: Overall, the participants perceived the patient-centred prophylaxis approach as helpful.  Improved understanding: The patient-centred prophylaxis approach enhanced communication with the clinical team, increased the understanding of haemophilia and pharmacokinetics of coagulation factor and facilitated treatment decisions. |
| Walsh  2021  USA [[75]](https://paperpile.com/c/jHvZKb/J4tx9) | To describe diagnostic experiences and identify specific health information needs of male premutation carriers | Qualitative: interviews analyzed with direct content analysis | Needs of information  Facilitators and barriers to obtain health information | Information needs: Participants desired information about inheritance, symptoms, expectations for disease, and actions available to slow the progression.  Facilitators to obtaining health information: Facilitators to obtaining health information included healthcare provider knowledge, positive experiences with providers, beneficial family dynamics, participating in research, and access to experts. Barriers to obtaining health information: Barriers to obtaining health information included lack of personal knowledge, lack of healthcare provider knowledge, negative experiences with providers, and uncertainty. |

[1. Akanuwe JNA, Laparidou D, Curtis F, Jackson J, Hodgson TL, Siriwardena AN. Exploring the experiences of having Guillain-Barré Syndrome: A qualitative interview study. Health Expect [Internet]. 2020;23:1338–49. Available from:](http://paperpile.com/b/jHvZKb/yyhKq) <https://onlinelibrary.wiley.com/doi/10.1111/hex.13116>

[2. Arnold E, Lane S, Webert KE, Chan A, Walker I, Tufts J, et al. What should men living with haemophilia need to know? The perspectives of Canadian men with haemophilia. Haemophilia [Internet]. 2014;20:219–25. Available from:](http://paperpile.com/b/jHvZKb/R0xJi) <https://onlinelibrary.wiley.com/doi/10.1111/hae.12297>

[3. Arran N, Craufurd D, Simpson J. Illness perceptions, coping styles and psychological distress in adults with Huntington’s disease. Psychol Health Med [Internet]. 2014;19:169–79. Available from:](http://paperpile.com/b/jHvZKb/vwWIQ) <http://dx.doi.org/10.1080/13548506.2013.802355>

[4. Arya S, Wilton P, Page D, Boma-Fischer L, Floros G, Winikoff R, et al. “They don’t really take my bleeds seriously”: Barriers to care for women with inherited bleeding disorders. J Thromb Haemost [Internet]. 2021;19:1506–14. Available from:](http://paperpile.com/b/jHvZKb/KCG5d) <https://www.sciencedirect.com/science/article/pii/S1538783622007905>

[5. Beinke K, O’Callaghan F, Morrissey S. Illness Perceptions of Cystic Fibrosis: A Comparison of Young Adults with CF and Same-Aged Peers. Behav Med [Internet]. 2017;43:40–6. Available from:](http://paperpile.com/b/jHvZKb/Rh7q9) <http://dx.doi.org/10.1080/08964289.2015.1045824>

[6. Bhatt N, Boggio L, Simpson ML. Using an educational intervention to assess and improve disease-specific knowledge and health literacy and numeracy in adolescents and young adults with haemophilia A and B. Haemophilia [Internet]. 2021;27:229–36. Available from:](http://paperpile.com/b/jHvZKb/r4OHq) <https://onlinelibrary.wiley.com/doi/abs/10.1111/hae.14228>

[7. Bogart KR, Frandrup E, Locke T, Thompson H, Weber N, Yates J, et al. “Rare place where I feel normal”: Perceptions of a social support conference among parents of and people with Moebius syndrome. Res Dev Disabil [Internet]. 2017;64:143–51. Available from:](http://paperpile.com/b/jHvZKb/uxUeK) <https://www.sciencedirect.com/science/article/pii/S0891422217300963>

[8. Borghi L, Moreschi C, Toscano A, Comber P, Vegni E. The PKU & ME study: A qualitative exploration, through co-creative sessions, of attitudes and experience of the disease among adults with phenylketonuria in Italy. Molecular Genetics and Metabolism Reports [Internet]. 2020;23:100585. Available from:](http://paperpile.com/b/jHvZKb/aSxtO) <https://www.sciencedirect.com/science/article/pii/S2214426920300318>

[9. Braisch U, Martinez-Horta S, MacDonald M, Orth M. Important but not enough - information about HD related topics and peer and professional support for young adults from HD families. J Huntingtons Dis [Internet]. 2016 [cited 2023 Jun 6];5:379–87. Available from:](http://paperpile.com/b/jHvZKb/TUYBT) <https://content.iospress.com/articles/journal-of-huntingtons-disease/jhd160218>

[10. Bryson B, Bogart K, Atwood M, Fraser K, Locke T, Pugh K, et al. Navigating the unknown: A content analysis of the unique challenges faced by adults with rare diseases. J Health Psychol [Internet]. 2021;26:623–35. Available from:](http://paperpile.com/b/jHvZKb/aJkrj) <http://dx.doi.org/10.1177/1359105319828150>

[11. Carpenter DM, DeVellis RF, Hogan SL, Fisher EB, DeVellis BM, Jordan JM. Use and perceived credibility of medication information sources for patients with a rare illness: differences by gender. J Health Commun [Internet]. 2011;16:629–42. Available from:](http://paperpile.com/b/jHvZKb/d8RqX) <http://dx.doi.org/10.1080/10810730.2011.551995>

[12. Chaleat-Valayer E, Amélie Z, Marie-Hélène B, Perretant I, Sandrine T. Therapeutic education program for patients with hypermobile Ehlers-Danlos syndrome: Feasibility and satisfaction of the participants. Education thérapeutique du patient - Therapeutic patient education [Internet]. 2019 [cited 2023 Jun 14];11:10202. Available from:](http://paperpile.com/b/jHvZKb/ah01q) <https://www.researchgate.net/publication/332063782_Therapeutic_education_program_for_patients_with_hypermobile_Ehlers-Danlos_syndrome_Feasibility_and_satisfaction_of_the_participants>

[13. Chaudhry SR, Keaton M, Nasr SZ. Evaluation of a cystic fibrosis transition program from pediatric to adult care. Pediatr Pulmonol [Internet]. 2013;48:658–65. Available from:](http://paperpile.com/b/jHvZKb/YrUf8) <http://dx.doi.org/10.1002/ppul.22647>

[14. Coathup V, Teare HJA, Minari J, Yoshizawa G, Kaye J, Takahashi MP, et al. Using digital technologies to engage with medical research: views of myotonic dystrophy patients in Japan. BMC Med Ethics [Internet]. 2016;17:51. Available from:](http://paperpile.com/b/jHvZKb/eAYez) <http://dx.doi.org/10.1186/s12910-016-0132-2>

[15. David V, Feldman D, Danner-Boucher I, Rhun AL, Guyomarch B, Ravilly S, et al. Identifying the educational needs of lung transplant recipients with cystic fibrosis. Prog Transplant [Internet]. 2015;25:18–25. Available from:](http://paperpile.com/b/jHvZKb/gGqth) <http://dx.doi.org/10.7182/pit2015526>

[16. De la Corte-Rodriguez H, Rodriguez-Merchan EC, Alvarez-Roman T, Martin-Salces M, Garcia-Barcenilla S, Jimenez-Yuste V. Health education and empowerment in adult patients with haemophilia. Expert Rev Hematol [Internet]. 2019;12:989–95. Available from:](http://paperpile.com/b/jHvZKb/xbkJH) <http://dx.doi.org/10.1080/17474086.2019.1650640>

[17. Delisle VC, Gumuchian ST, Pelaez S, Malcarne VL, El-Baalbaki G, Körner A, et al. Reasons for non-participation in scleroderma support groups. Clin Exp Rheumatol [Internet]. 2016;34 Suppl 100:56–62. Available from:](http://paperpile.com/b/jHvZKb/FFTpG) <https://www.ncbi.nlm.nih.gov/pubmed/26950221>

[18. Dellon EP, Helms SW, Hailey CE, Shay R, Carney SD, Schmidt HJ, et al. Exploring knowledge and perceptions of palliative care to inform integration of palliative care education into cystic fibrosis care. Pediatr Pulmonol [Internet]. 2018;53:1218–24. Available from:](http://paperpile.com/b/jHvZKb/5cSj6) <http://dx.doi.org/10.1002/ppul.24073>

[19. Depping MK, Uhlenbusch N, Härter M, Schramm C, Löwe B. Efficacy of a Brief, Peer-Delivered Self-management Intervention for Patients With Rare Chronic Diseases: A Randomized Clinical Trial. JAMA Psychiatry [Internet]. 2021;78:607–15. Available from:](http://paperpile.com/b/jHvZKb/yQdqR) <http://dx.doi.org/10.1001/jamapsychiatry.2020.4783>

[20. Dicianno BE, Lovelace J, Peele P, Fassinger C, Houck P, Bursic A, et al. Effectiveness of a Wellness Program for Individuals With Spina Bifida and Spinal Cord Injury Within an Integrated Delivery System. Arch Phys Med Rehabil [Internet]. 2016;97:1969–78. Available from:](http://paperpile.com/b/jHvZKb/aUXam) <http://dx.doi.org/10.1016/j.apmr.2016.05.014>

[21. Domaradzki J. Family caregivers’ experiences with healthcare services--a case of Huntington’s disease. Psychiatr Pol [Internet]. 2016;50:375–91. Available from:](http://paperpile.com/b/jHvZKb/DIUcI) <http://dx.doi.org/10.12740/PP/59103>

[22. Dwyer AA, Quinton R, Morin D, Pitteloud N. Identifying the unmet health needs of patients with congenital hypogonadotropic hypogonadism using a web-based needs assessment: implications for online interventions and peer-to-peer support. Orphanet J Rare Dis [Internet]. 2014;9:83. Available from:](http://paperpile.com/b/jHvZKb/Vr9L8) <https://ojrd.biomedcentral.com/articles/10.1186/1750-1172-9-83>

[23. Etchegary H. Healthcare experiences of families affected by Huntington disease: need for improved care. Chronic Illn [Internet]. 2011;7:225–38. Available from:](http://paperpile.com/b/jHvZKb/SGJDM) <http://dx.doi.org/10.1177/1742395311403637>

[24. Flewelling KD, Sellers DE, Sawicki GS, Robinson WM, Dill EJ. Social support is associated with fewer reported symptoms and decreased treatment burden in adults with cystic fibrosis. J Cyst Fibros [Internet]. 2019;18:572–6. Available from:](http://paperpile.com/b/jHvZKb/2nE7M) <http://dx.doi.org/10.1016/j.jcf.2019.01.013>

[25. Foley G, Timonen V, Hardiman O. Understanding psycho-social processes underpinning engagement with services in motor neurone disease: a qualitative study. Palliat Med [Internet]. 2014;28:318–25. Available from:](http://paperpile.com/b/jHvZKb/MTsUv) <http://dx.doi.org/10.1177/0269216313512013>

[26. Garrino L, Picco E, Finiguerra I, Rossi D, Simone P, Roccatello D. Living with and treating rare diseases: experiences of patients and professional health care providers. Qual Health Res [Internet]. 2015;25:636–51. Available from:](http://paperpile.com/b/jHvZKb/USEvG) <http://dx.doi.org/10.1177/1049732315570116>

[27. Gumuchian ST, Peláez S, Delisle VC, Carrier M-E, Jewett LR, El-Baalbaki G, et al. Understanding coping strategies among people living with scleroderma: a focus group study. Disabil Rehabil [Internet]. 2018;40:3012–21. Available from:](http://paperpile.com/b/jHvZKb/eUS6t) <http://dx.doi.org/10.1080/09638288.2017.1365954>

[28. Hiermeier UM, Baker C, Bourke JP. Exploring the acceptability of implantable defibrillators in patients with cardiac dystrophinopathy and carers. Open Heart [Internet]. 2020;7:e001230. Available from:](http://paperpile.com/b/jHvZKb/N9M0G) <http://dx.doi.org/10.1136/openhrt-2019-001230>

[29. Hoefnagels JW, Fischer K, Bos RAT, Driessens MHE, Meijer SLA, Schutgens REG, et al. A feasibility study on two tailored interventions to improve adherence in adults with haemophilia. Pilot Feasibility Stud [Internet]. 2020;6:189. Available from:](http://paperpile.com/b/jHvZKb/Drrpi) <http://dx.doi.org/10.1186/s40814-020-00723-w>

[30. Ioannou L, Massie J, Collins V, McClaren B, Delatycki MB. Population-based genetic screening for cystic fibrosis: attitudes and outcomes. Public Health Genomics [Internet]. 2010;13:449–56. Available from:](http://paperpile.com/b/jHvZKb/FbPsE) <http://dx.doi.org/10.1159/000276544>

[31. Jackson AD, Kirwan L, Gibney S, Jeleniewska P, Fletcher G, Doyle G. Associations between health literacy and patient outcomes in adolescents and young adults with cystic fibrosis. Eur J Public Health [Internet]. 2020;30:112–8. Available from:](http://paperpile.com/b/jHvZKb/A1Lks) <http://dx.doi.org/10.1093/eurpub/ckz148>

[32. Katavic SS, Tanackovic SF, Badurina B. Illness perception and information behaviour of patients with rare chronic diseases. Inflamm Res [Internet]. 2016 [cited 2023 Jun 14];21. Available from:](http://paperpile.com/b/jHvZKb/RJ302) <http://dx.doi.org/10.1111/hir.12261>

[33. Stanarević Katavić S. Health information behaviour of rare disease patients: seeking, finding and sharing health information. Health Info Libr J [Internet]. 2019;36:341–56. Available from:](http://paperpile.com/b/jHvZKb/t7KX4) <http://dx.doi.org/10.1111/hir.12261>

[34. Kazmerski TM, Gmelin T, Slocum B, Borrero S, Miller E. Attitudes and Decision Making Related to Pregnancy Among Young Women with Cystic Fibrosis. Matern Child Health J [Internet]. 2017;21:818–24. Available from:](http://paperpile.com/b/jHvZKb/gW49Y) <http://dx.doi.org/10.1007/s10995-016-2181-z>

[35. Kesselheim AS, McGraw S, Thompson L, O’Keefe K, Gagne JJ. Development and use of new therapeutics for rare diseases: views from patients, caregivers, and advocates. Patient [Internet]. 2015;8:75–84. Available from:](http://paperpile.com/b/jHvZKb/sghIR) <http://dx.doi.org/10.1007/s40271-014-0096-6>

[36. Keyte R, Egan H, Nash EF, Regan A, Jackson C, Mantzios M. An exploration into experiences and attitudes regarding risky health behaviours in an adult cystic fibrosis population. Psychol Health Med [Internet]. 2020;25:1013–9. Available from:](http://paperpile.com/b/jHvZKb/gQqvi) <http://dx.doi.org/10.1080/13548506.2019.1706750>

[37. Kirk S, Milnes L. An exploration of how young people and parents use online support in the context of living with cystic fibrosis. Health Expect [Internet]. 2016;19:309–21. Available from:](http://paperpile.com/b/jHvZKb/7Ed62) <http://dx.doi.org/10.1111/hex.12352>

[38. Kurtz NS, Cote C, Heatwole C, Gagnon C, Youssof S. Patient-reported disease burden in oculopharyngeal muscular dystrophy. Muscle Nerve [Internet]. 2019;60:724–31. Available from:](http://paperpile.com/b/jHvZKb/NyU4c) <https://onlinelibrary.wiley.com/doi/abs/10.1002/mus.26712>

[39. Laberge L, Prévost C, Perron M, Mathieu J, Auclair J, Gaudreault M, et al. Clinical and genetic knowledge and attitudes of patients with myotonic dystrophy type 1. Public Health Genomics [Internet]. 2010;13:424–30. Available from:](http://paperpile.com/b/jHvZKb/WgRgD) <http://dx.doi.org/10.1159/000316238>

[40. LaDonna KA, Ghavanini AA, Venance SL. Truths and misinformation: a qualitative exploration of myotonic dystrophy. Can J Neurol Sci [Internet]. 2015;42:187–94. Available from:](http://paperpile.com/b/jHvZKb/QtFKC) <http://dx.doi.org/10.1017/cjn.2015.26>

[41. le Doré S, Grinda N, Ferré E, Roussel-Robert V, Frotscher B, Chamouni P, et al. The hemarthrosis-simulating knee model: A useful tool for individualized education in patients with hemophilia (GEFACET study). J Blood Med [Internet]. 2021;12:133–8. Available from:](http://paperpile.com/b/jHvZKb/wMyim) <https://www.tandfonline.com/doi/abs/10.2147/JBM.S280032>

[42. Lewis KL, John B, Condren M, Carter SM. Evaluation of Medication-related Self-care Skills in Patients With Cystic Fibrosis. J Pediatr Pharmacol Ther [Internet]. 2016;21:502–11. Available from:](http://paperpile.com/b/jHvZKb/OWQ6v) <http://dx.doi.org/10.5863/1551-6776-21.6.502>

[43. Lindsay S, Fellin M, Cruickshank H, McPherson A, Maxwell J. Youth and parents’ experiences of a new inter-agency transition model for spina bifida compared to youth who did not take part in the model. Disabil Health J [Internet]. 2016;9:705–12. Available from:](http://paperpile.com/b/jHvZKb/k1qQm) <http://dx.doi.org/10.1016/j.dhjo.2016.05.009>

[44. Lindvall K, Colstrup L, Loogna K, Wollter I, Grönhaug S. Knowledge of disease and adherence in adult patients with haemophilia. Haemophilia [Internet]. 2010;16:592–6. Available from:](http://paperpile.com/b/jHvZKb/TLAot) <http://dx.doi.org/10.1111/j.1365-2516.2009.02189.x>

[45. Litzkendorf S, Frank M, Babac A, Rosenfeldt D, Schauer F, Hartz T, et al. Use and importance of different information sources among patients with rare diseases and their relatives over time: a qualitative study. BMC Public Health [Internet]. 2020;20:860. Available from:](http://paperpile.com/b/jHvZKb/qtT9o) <http://dx.doi.org/10.1186/s12889-020-08926-9>

[46. Lonabaugh KP, O’Neal KS, McIntosh H, Condren M. Cystic fibrosis-related education: Are we meeting patient and caregiver expectations? Patient Educ Couns [Internet]. 2018;101:1865–70. Available from:](http://paperpile.com/b/jHvZKb/MJZh5) <http://dx.doi.org/10.1016/j.pec.2018.06.004>

[47. Mälstam E, Bensing S, Asaba E. Everyday managing and living with autoimmune Addison’s disease: Exploring experiences using photovoice methods. Scand J Occup Ther [Internet]. 2018;25:358–70. Available from:](http://paperpile.com/b/jHvZKb/Ub991) <http://dx.doi.org/10.1080/11038128.2018.1502351>

[48. Merker VL, McDannold S, Riklin E, Talaei-Khoei M, Sheridan MR, Jordan JT, et al. Health literacy assessment in adults with neurofibromatosis: electronic and short-form measurement using FCCHL and Health LiTT. J Neurooncol [Internet]. 2018;136:335–42. Available from:](http://paperpile.com/b/jHvZKb/F0MxJ) <http://dx.doi.org/10.1007/s11060-017-2657-8>

[49. Mohan R, Radhakrishnan N, Varadarajan M, Anand S. Assessing the current knowledge, attitude and behaviour of adolescents and young adults living with haemophilia. Haemophilia [Internet]. 2021;27:e180–6. Available from:](http://paperpile.com/b/jHvZKb/SR6SC) <http://dx.doi.org/10.1111/hae.14229>

[50. Molster C, Urwin D, Di Pietro L, Fookes M, Petrie D, van der Laan S, et al. Survey of healthcare experiences of Australian adults living with rare diseases. Orphanet J Rare Dis [Internet]. 2016;11:30. Available from:](http://paperpile.com/b/jHvZKb/MJmjI) <http://dx.doi.org/10.1186/s13023-016-0409-z>

[51. Mooney J, Poland F, Spalding N, Scott DGI. “In One Ear and Out the Other–It”s a Lot to Take in’: A Qualitative Study Exploring the Informational Needs of Patients with ANCA‐Associated Vasculitis. Musculoskeletal [Internet]. 2013; Available from:](http://paperpile.com/b/jHvZKb/FI2wI) <https://onlinelibrary.wiley.com/doi/abs/10.1002/msc.1030>

[52. Mulders G, de Wee EM, Vahedi Nikbakht-Van de Sande MCVM, Kruip MJHA, Elfrink EJ, Leebeek FWG. E-learning improves knowledge and practical skills in haemophilia patients on home treatment: a randomized controlled trial. Haemophilia [Internet]. 2012;18:693–8. Available from:](http://paperpile.com/b/jHvZKb/waPPC) <https://onlinelibrary.wiley.com/doi/10.1111/j.1365-2516.2012.02786.x>

[53. Naik H, Shenbagam S, Go AM, Balwani M. Psychosocial issues in erythropoietic protoporphyria - the perspective of parents, children, and young adults: A qualitative study. Mol Genet Metab [Internet]. 2019;128:314–9. Available from:](http://paperpile.com/b/jHvZKb/I8YSJ) <http://dx.doi.org/10.1016/j.ymgme.2019.01.023>

[54. O’Mahar K, Holmbeck GN, Jandasek B, Zukerman J. A camp-based intervention targeting independence among individuals with spina bifida. J Pediatr Psychol [Internet]. 2010;35:848–56. Available from:](http://paperpile.com/b/jHvZKb/9Xbav) <http://dx.doi.org/10.1093/jpepsy/jsp125>

[55. Pakhale S, Baron J, Armstrong M, Tasca G, Gaudet E, Aaron SD, et al. Lost in translation? How adults living with Cystic Fibrosis understand treatment recommendations from their healthcare providers, and the impact on adherence to therapy. Patient Educ Couns [Internet]. 2016;99:1319–24. Available from:](http://paperpile.com/b/jHvZKb/JbQBZ) <http://dx.doi.org/10.1016/j.pec.2016.03.023>

[56. Parvizi MM, Lankarani KB, Handjani F, Ghahramani S, Parvizi Z, Rousta S. Health literacy in patients with epidermolysis bullosa in Iran. J Educ Health Promot [Internet]. 2017;6:105. Available from:](http://paperpile.com/b/jHvZKb/xPYI4) <http://dx.doi.org/10.4103/jehp.jehp_64_17>

[57. Raphaelis S, Mayer H, Ott S, Hornung R, Senn B. Effects of Written Information and Counseling on Illness-Related Uncertainty in Women With Vulvar Neoplasia. Oncol Nurs Forum [Internet]. 2018;45:748–60. Available from:](http://paperpile.com/b/jHvZKb/ZBcTR) <http://dx.doi.org/10.1188/18.ONF.748-760>

[58. Riklin E, Talaei-Khoei M, Merker VL, Sheridan MR, Jordan JT, Plotkin SR, et al. First report of factors associated with satisfaction in patients with neurofibromatosis. Am J Med Genet A [Internet]. 2017;173:671–7. Available from:](http://paperpile.com/b/jHvZKb/MRsMG) <http://dx.doi.org/10.1002/ajmg.a.38079>

[59. Ringqvist K, Borg K, Möller MC. Tolerability and psychological effects of a multimodal day-care rehabilitation program for persons with Huntington’s disease. J Rehabil Med [Internet]. 2021;53:jrm00143. Available from:](http://paperpile.com/b/jHvZKb/bQoRK) <http://dx.doi.org/10.2340/16501977-2748>

[60. Rosnau K, Hashmi SS, Northrup H, Slopis J, Noblin S, Ashfaq M. Knowledge and Self-Esteem of Individuals with Neurofibromatosis Type 1 (NF1). J Genet Couns [Internet]. 2017;26:620–7. Available from:](http://paperpile.com/b/jHvZKb/uAQtI) <http://dx.doi.org/10.1007/s10897-016-0036-9>

[61. Rovira-Moreno E, Abuli A, Codina-Sola M, Valenzuela I, Serra-Juhe C, Cuscó I, et al. Beyond the disease itself: A cross-cutting educational initiative for patients and families with rare diseases. J Genet Couns [Internet]. 2021;30:693–700. Available from:](http://paperpile.com/b/jHvZKb/bOYFm) <http://dx.doi.org/10.1002/jgc4.1354>

[62. Salvatore V, Gilstrap A, Williams KR, Thorat S, Stevenson M, Gwosdow AR, et al. Evaluating the impact of peer support and connection on the quality of life of patients with familial chylomicronemia syndrome. Expert Opinion on Orphan Drugs [Internet]. 2018;6:497–505. Available from:](http://paperpile.com/b/jHvZKb/oj9o4) <https://doi.org/10.1080/21678707.2018.1505495>

[63. Shepherd LM, Tahrani AA, Inman C, Arlt W, Carrick-Sen DM. Exploration of knowledge and understanding in patients with primary adrenal insufficiency: a mixed methods study. BMC Endocr Disord [Internet]. 2017;17:47. Available from:](http://paperpile.com/b/jHvZKb/QTft0) <http://dx.doi.org/10.1186/s12902-017-0196-0>

[64. Shoshan L, Ben-Zvi D, Meyer S, Katz-Leurer M. Sexuality in relation to independence in daily functions among young people with spina bifida living in Israel. Rehabil Nurs [Internet]. 2012;37:11–7; quiz 17–8. Available from:](http://paperpile.com/b/jHvZKb/c2HkT) <http://dx.doi.org/10.1002/RNJ.00002>

[65. Skirton H, Williams JK, Jackson Barnette J, Paulsen JS. Huntington disease: families’ experiences of healthcare services. J Adv Nurs [Internet]. 2010;66:500–10. Available from:](http://paperpile.com/b/jHvZKb/wuwkK) <http://dx.doi.org/10.1111/j.1365-2648.2009.05217.x>

[66. Smedley RM, Coulson NS. Genetic testing for Huntington’s disease: A thematic analysis of online support community messages. J Health Psychol [Internet]. 2021;26:580–94. Available from:](http://paperpile.com/b/jHvZKb/iKCP3) <http://dx.doi.org/10.1177/1359105319826340>

[67. Smolich L, Charen K, Sherman SL. Health knowledge of women with a fragile X premutation: Improving understanding with targeted educational material. J Genet Couns [Internet]. 2020;29:983–91. Available from:](http://paperpile.com/b/jHvZKb/w6EQp) <http://dx.doi.org/10.1002/jgc4.1222>

[68. Socha Hernandez AV, Deeks LS, Shield AJ. Understanding medication safety and Charcot-Marie-Tooth disease: a patient perspective. Int J Clin Pharm [Internet]. 2020;42:1507–14. Available from:](http://paperpile.com/b/jHvZKb/ak9YJ) <http://dx.doi.org/10.1007/s11096-020-01123-z>

[69. Stubberud J, Langenbahn D, Levine B, Stanghelle J, Schanke A-K. Emotional health and coping in spina bifida after goal management training: a randomized controlled trial. Rehabil Psychol [Internet]. 2015;60:1–16. Available from:](http://paperpile.com/b/jHvZKb/p63HC) <http://dx.doi.org/10.1037/rep0000018>

[70. Sylvain C, Lamothe L, Berthiaume Y, Rabasa-Lhoret R. How patients’ representations of cystic fibrosis-related diabetes inform their health behaviours. Psychol Health [Internet]. 2016;31:1129–44. Available from:](http://paperpile.com/b/jHvZKb/Mxyfc) <http://dx.doi.org/10.1080/08870446.2016.1183008>

[71. Takeuchi T, Muraoka K, Yamada M, Nishio Y, Hozumi I. Living with idiopathic basal ganglia calcification 3: a qualitative study describing the lives and illness of people diagnosed with a rare neurological disease. Springerplus [Internet]. 2016;5:1713. Available from:](http://paperpile.com/b/jHvZKb/KQQEk) <http://dx.doi.org/10.1186/s40064-016-3390-z>

[72. Théaudin M, Cauquil C, Antonini T, Algalarrondo V, Labeyrie C, Aycaguer S, et al. Familial amyloid polyneuropathy: elaboration of a therapeutic patient education programme, “EdAmyl.” Amyloid [Internet]. 2014;21:225–30. Available from:](http://paperpile.com/b/jHvZKb/peNAk) <http://dx.doi.org/10.3109/13506129.2014.941463>

[73. Torres-Ortuño A, Cuesta-Barriuso R, Nieto-Munuera J, Galindo-Piñana P, López-Pina J-A. The behaviour and perception of illness: modulating variables of adherence in patients with haemophilia. Vox Sang [Internet]. 2018; Available from:](http://paperpile.com/b/jHvZKb/VD66O) <http://dx.doi.org/10.1111/vox.12669>

[74. van Balen EC, Krawczyk M, Gue D, Jackson S, Gouw SC, van der Bom JG, et al. Patient-centred care in haemophilia: Patient perspectives on visualization and participation in decision-making. Haemophilia [Internet]. 2019;25:938–45. Available from:](http://paperpile.com/b/jHvZKb/BfIZ7) <http://dx.doi.org/10.1111/hae.13830>

[75. Walsh MB, Charen K, Shubeck L, McConkie-Rosell A, Ali N, Bellcross C, et al. Men with an FMR1 premutation and their health education needs. J Genet Couns [Internet]. 2021;30:1156–67. Available from:](http://paperpile.com/b/jHvZKb/J4tx9) <http://dx.doi.org/10.1002/jgc4.1399>
