# Supplementary material for: A scoping review of health literacy in rare disorders: key issues and research directions
Source: Orphanet J Rare Dis. 2024 Sep 6;19:328. doi: 10.1186/s13023-024-03332-5 (PMC11380335; doi:10.1186/s13023-024-03332-5)
Supplement: Supplementary file 6 — Supplementary Material 6 [file 13023_2024_3332_MOESM6_ESM.docx]

Additional file 6

Table 4. Standardised outcome measures.

| Outcome | Diagnosis | Outcome measure | Reference |
| --- | --- | --- | --- |
| Quality of life | Huntington’s disease  Hypermobile Ehlers-Danlos syndrome  People with rare chronic diseases  Spina Bifida and Spinar Cord Injury  Cystic fibrosis  Haemophilia  Spina Bifida | SF36  SF12  World Health Organization Quality of Life-Brief Form (WHOQOL-BREF)  Cystic Fibrosis Questionnaire – Revised (CFQ-R)  EuroQol Visual Analogue Scale (EQ-VAS) | 1. Arran et al 2014 [[1]](https://paperpile.com/c/Qzhbp1/te8cJ)  2. Chaleat-Valayer 2019 [[2]](https://paperpile.com/c/Qzhbp1/XqnRJ)  3. Depping 2021 [[3]](https://paperpile.com/c/Qzhbp1/quD6L)  4. Dicianno 2016 [[4]](https://paperpile.com/c/Qzhbp1/G8RGT)  5. Flewelling 2019 [[5]](https://paperpile.com/c/Qzhbp1/gx31d)  6. Hoefnagels 2020 [[6]](https://paperpile.com/c/Qzhbp1/Pcwfm)  7. Jackson 2020 [[7]](https://paperpile.com/c/Qzhbp1/wzXHk)  8. Ringqvist 2021 [[8]](https://paperpile.com/c/Qzhbp1/Jy23I)  9. Stubberud 2015 [[9]](https://paperpile.com/c/Qzhbp1/9HEKo)  10. Torres-Ortuno 2018 [[10]](https://paperpile.com/c/Qzhbp1/0dVRx) |
| Anxiety | Huntington’s disease  Hypermobile Ehlers-Danlos syndrome  People with rare chronic diseases  Cystic fibrosis  Spina Bifida | Hospital anxiety and depression scale (HADS)  Generalized Anxiety Disorder Scale  State Trait Anxiety Inventory (STAI)  Hopkins Symptom Checklist 25 (HSCL-25) | 1. Arran et al 2014 [[1]](https://paperpile.com/c/Qzhbp1/te8cJ)  2. Chaleat-Valayer 2019 [[2]](https://paperpile.com/c/Qzhbp1/XqnRJ)  3. Depping 2021 [[3]](https://paperpile.com/c/Qzhbp1/quD6L)  4. Ioannou 2010 [[11]](https://paperpile.com/c/Qzhbp1/Ss4O9)  5. Ringqvist 2021 [[8]](https://paperpile.com/c/Qzhbp1/Jy23I)  6. Rovira-Moreno  2020 [[12]](https://paperpile.com/c/Qzhbp1/xNhA3)  7. Stubberud 2015 [[9]](https://paperpile.com/c/Qzhbp1/9HEKo) |
| Depression | Huntington’s disease  Hypermobile Ehlers-Danlos syndrome  People with rare chronic diseases  Spina Bifida and Spinar Cord Injury  Spina Bifida | Hospital anxiety and depression scale (HADS)  Patient Health Questionnaire (PHQ-9)  Beck Depression Inventory-II (BDI-II)  Hopkins Symptom Checklist 25 (HSCL-25) | 1. Arran et al 2014 [[1]](https://paperpile.com/c/Qzhbp1/te8cJ)  2. Chaleat-Valayer 2019 [[2]](https://paperpile.com/c/Qzhbp1/XqnRJ)  3. Depping 2021 [[3]](https://paperpile.com/c/Qzhbp1/quD6L)  4. Dicianno 2016 [[4]](https://paperpile.com/c/Qzhbp1/G8RGT)  5. Ringqvist 2021 [[8]](https://paperpile.com/c/Qzhbp1/Jy23I)  6. Stubberud 2015 [[9]](https://paperpile.com/c/Qzhbp1/9HEKo) |
| Illness Perceptions | Huntington’s disease  Cystic fibrosis  People with rare chronic diseases  Haemophilia  Rare diagnoses (systemic lupus erythematous, Scleroderma and myasthenia gravis) | Illness Perceptions Questionnaire-Revised (IPQ-R)  Brief Illness Perception Questionnaire (Brief IPQ) | 1. Arran et al 2014 [[1]](https://paperpile.com/c/Qzhbp1/te8cJ)  2. Beinke, 2017 [[13]](https://paperpile.com/c/Qzhbp1/52hJF)  3. Depping 2021 [[3]](https://paperpile.com/c/Qzhbp1/quD6L)  4. Hoefnagels 2020 [[6]](https://paperpile.com/c/Qzhbp1/Pcwfm)  5. Katavic 2016 [[14]](https://paperpile.com/c/Qzhbp1/R3bXF)  6. Torres-Ortuno 2018 [[10]](https://paperpile.com/c/Qzhbp1/0dVRx) |
| Health literacy | Haemophilia  Congenital hypogonadotropic hypogonadism  Cystic Fibrosis  Neurofibromatosis (NF1 and NF2) and schwannomatosis  Epidermolysis bullosa (EB) | Rapid Estimate of Adolescent Literacy in Medicine (REALM-Teen)  HLS-EUQ16  Adapted functional, communicative, and critical health literacy scale (adapted FCCHL)  Health literacy assessment using talking touchscreen technology (Health LiTT)  Health Literacy for Iranian Adults (HELIA) | 1. Bhatt, 2021 [[15]](https://paperpile.com/c/Qzhbp1/0CZrE)  2. Dwyer 2014 [[16]](https://paperpile.com/c/Qzhbp1/dcUZH)  3. Jackson 2020 [[7]](https://paperpile.com/c/Qzhbp1/wzXHk)  4. Merker 2018 + Riklin,  2017 [[17,18]](https://paperpile.com/c/Qzhbp1/FTkFY+is7Oo)  5. Parvizi 2017 [[19]](https://paperpile.com/c/Qzhbp1/A38fZ) |
| Coping | Huntington’s disease  Hypermobile Ehlers-Danlos syndrome  Spina Bifida  Haemophilia | Brief COPE Coping Strategies Questionnaire (CSQ)  General Coping Questionnaire (GCQ)  Coping strategies inventory (CSI) | 1. Arran et al 2014 [[1]](https://paperpile.com/c/Qzhbp1/te8cJ)  2. Chaleat-Valayer 2019 [[2]](https://paperpile.com/c/Qzhbp1/XqnRJ)  3. Stubberud 2015 [[9]](https://paperpile.com/c/Qzhbp1/9HEKo)  4. Torres-Ortuno 2018 [[10]](https://paperpile.com/c/Qzhbp1/0dVRx) |
| Self-management skills | People with rare chronic diseases  Haemophilia  Cystic fibrosis | Health Education Impact Questionnaire (heiQ)  Modified Transition Readiness Assessment Questionnaire (TRAQ) | 1. Depping 2021 [[3]](https://paperpile.com/c/Qzhbp1/quD6L)  2. Hoefnagels 2020 [[6]](https://paperpile.com/c/Qzhbp1/Pcwfm)  3. Lewis 2016 [[20]](https://paperpile.com/c/Qzhbp1/3b8df) |
| Medication adherence | Cystic fibrosis  Haemophilia | Tool for Adherence Behaviour Screening (TABS)  Hemophilia Regimen Treatment Adherence Scale –Prophylaxis (VERITAS-Pro) questionnaire | 1. Flewelling 2019 [[5]](https://paperpile.com/c/Qzhbp1/gx31d)  2. Hoefnagels 2020 [[6]](https://paperpile.com/c/Qzhbp1/Pcwfm)  3. Torres-Ortuno 2018 [[10]](https://paperpile.com/c/Qzhbp1/0dVRx) |
| Social Support | People with rare chronic diseases  Cystic fibrosis | Social Support Questionnaire  Inter-personal Support Evaluation List (ISEL) | 1. Depping 2021 [[3]](https://paperpile.com/c/Qzhbp1/quD6L)  2. Flewelling 2019 [[5]](https://paperpile.com/c/Qzhbp1/gx31d) |
| cognition | Haemophilia | Montreal Cognitive Assessment (MoCA) | Bhatt, 2021 [[15]](https://paperpile.com/c/Qzhbp1/0CZrE) |
| arthropathy | Haemophilia | Haemophilia Joint Health Score (HJHS) | Bhatt, 2021 [[15]](https://paperpile.com/c/Qzhbp1/0CZrE) |
| Body image | Hypermobile Ehlers-Danlos syndrome | Body Image Questionnaire (QIC) | Chaleat-Valayer 2019 [[2]](https://paperpile.com/c/Qzhbp1/XqnRJ) |
| Fatigue | Hypermobile Ehlers-Danlos syndrome | Fatigue Impact Scale | Chaleat-Valayer 2019 [[2]](https://paperpile.com/c/Qzhbp1/XqnRJ) |
| Goal attainment | Hypermobile Ehlers-Danlos syndrome | Goal attainment Scale (GAS) | Chaleat-Valayer 2019 [[2]](https://paperpile.com/c/Qzhbp1/XqnRJ) |
| Illness beliefs | People with rare chronic diseases | Illness Cognition Questionnaire (ICQ) | Depping  2021 [[3]](https://paperpile.com/c/Qzhbp1/quD6L) |
| Degree of handicap/degree of community participation | Spina Bifida and Spinar Cord Injury | Craig Handicap Assessment and Reporting Technique-Short Form (CHART-SF) | Dicianno 2016 [[4]](https://paperpile.com/c/Qzhbp1/G8RGT) |
| Perception of patient-centered care | Spina Bifida and Spinar Cord Injury | The Patient Assessment of Chronic Illness Care (PACIC) | Dicianno 2016 [[4]](https://paperpile.com/c/Qzhbp1/G8RGT) |
| Frequency, severity, and distress of medical symptoms | Cystic fibrosis | Memorial Symptom Assessment Scale (MSAS) | Flewelling 2019 [[5]](https://paperpile.com/c/Qzhbp1/gx31d) |
| Knowledge of disease management | Cystic fibrosis | Knowledge of Disease Management-CF (KDM_CF) scale | Lonabaugh 2018 [[21]](https://paperpile.com/c/Qzhbp1/806uw) |
| Knowledge of disease | Spina Bifida | Knowledge of Spina Bifida Measure KOSB | O`Mahar 2010 [[22]](https://paperpile.com/c/Qzhbp1/lQoo6) |
| Independence in spina-bifida related tasks | Spina Bifida | Spina Bifida Independence Survey (SBIS) | O`Mahar 2010 [[22]](https://paperpile.com/c/Qzhbp1/lQoo6) |
| Illness-related uncertainty | Vulvar neoplasia | Mishel Uncertainty in Illness Scale (MUIS-A) | Raphaelis 2018 [[23]](https://paperpile.com/c/Qzhbp1/nzkQ0) |
| Satisfaction  with the medical visit | Neurofibro-  matosis type 1, neurofibromatosis type 2, or schwannomatosis | Medical Interview Satisfaction Scale (MISS) | Riklin, 2017 [[18]](https://paperpile.com/c/Qzhbp1/is7Oo) |
| Medical provider’s communication skills | Neurofibro-  matosis type 1, neurofibromatosis type 2, or schwannomatosis | Adapted Consumer Assessment of Healthcare Providers and Systems Health Literacy Item Set (CAHPS-HL) | Riklin, 2017 [[18]](https://paperpile.com/c/Qzhbp1/is7Oo) |
| Sense of Coherence | Huntington’s disease | Sense of Coherence (SOC-29) | Ringqvist 2021 [[8]](https://paperpile.com/c/Qzhbp1/Jy23I) |
| Balance | Huntington’s disease | MiniBalance Evaluation Systems Test (Mini-BEST)  + Timed Up  and Go test (TUG) | Ringqvist 2021 [[8]](https://paperpile.com/c/Qzhbp1/Jy23I) |
| Aerobic capacity and endurance | Huntington’s disease | 6-Minute Walk Test (6MWT) | Ringqvist 2021 [[8]](https://paperpile.com/c/Qzhbp1/Jy23I) |
| Self-esteem | Neurofibromatosis 1 (NF1) | Rosenberg Self-Esteem Scale (RSES) | Rosnau K  2017 [[24]](https://paperpile.com/c/Qzhbp1/Xhu60) |
| Confidence with genetic knowledge | Rare disorders | Psychological Adaptation to Genetic Information Scale (PAGIS) | Rovira-Moreno  2020 [[12]](https://paperpile.com/c/Qzhbp1/xNhA3) |
| Knowledge of body parts and sexual knowledge and experience | Spina Bifida | The Sexual Knowledge Interview Schedule (SKIS) | Shoshan,  2012 [[25]](https://paperpile.com/c/Qzhbp1/ujeo4) |
| Independence in activities of daily living | Spina Bifida | The Functional Independence Measure (FIM) | Shoshan,  2012 [[25]](https://paperpile.com/c/Qzhbp1/ujeo4) |
| Availability and adequacy of community resources to support health care of the person with HD | Huntington disease | Community Health Care Services Scale | Skirton  2010 [[26]](https://paperpile.com/c/Qzhbp1/7oajv) |
| Dysexecutive syndrome | Spina Bifida | Dysexecutive Questionnaire (DEX) | Stubberud 2015 [[9]](https://paperpile.com/c/Qzhbp1/9HEKo) |
| Illness behavior | Haemophilia | Illness behavior questionnaire IBQ | Torres-Ortuno 2018 [[10]](https://paperpile.com/c/Qzhbp1/0dVRx) |
| Beliefs about medications | Haemophilia | Beliefs about Medication Questionnaire (BMQ) | Torres-Ortuno 2018 [[10]](https://paperpile.com/c/Qzhbp1/0dVRx) |

[1. Arran N, Craufurd D, Simpson J. Illness perceptions, coping styles and psychological distress in adults with Huntington’s disease. Psychol Health Med [Internet]. 2014;19:169–79. Available from:](http://paperpile.com/b/Qzhbp1/te8cJ) <http://dx.doi.org/10.1080/13548506.2013.802355>

[2. Chaleat-Valayer E, Amélie Z, Marie-Hélène B, Perretant I, Sandrine T. Therapeutic education program for patients with hypermobile Ehlers-Danlos syndrome: Feasibility and satisfaction of the participants. Education thérapeutique du patient - Therapeutic patient education [Internet]. 2019 [cited 2023 Jun 14];11:10202. Available from:](http://paperpile.com/b/Qzhbp1/XqnRJ) <https://www.researchgate.net/publication/332063782_Therapeutic_education_program_for_patients_with_hypermobile_Ehlers-Danlos_syndrome_Feasibility_and_satisfaction_of_the_participants>

[3. Depping MK, Uhlenbusch N, Härter M, Schramm C, Löwe B. Efficacy of a Brief, Peer-Delivered Self-management Intervention for Patients With Rare Chronic Diseases: A Randomized Clinical Trial. JAMA Psychiatry [Internet]. 2021;78:607–15. Available from:](http://paperpile.com/b/Qzhbp1/quD6L) <http://dx.doi.org/10.1001/jamapsychiatry.2020.4783>

[4. Dicianno BE, Lovelace J, Peele P, Fassinger C, Houck P, Bursic A, et al. Effectiveness of a Wellness Program for Individuals With Spina Bifida and Spinal Cord Injury Within an Integrated Delivery System. Arch Phys Med Rehabil [Internet]. 2016;97:1969–78. Available from:](http://paperpile.com/b/Qzhbp1/G8RGT) <http://dx.doi.org/10.1016/j.apmr.2016.05.014>

[5. Flewelling KD, Sellers DE, Sawicki GS, Robinson WM, Dill EJ. Social support is associated with fewer reported symptoms and decreased treatment burden in adults with cystic fibrosis. J Cyst Fibros [Internet]. 2019;18:572–6. Available from:](http://paperpile.com/b/Qzhbp1/gx31d) <http://dx.doi.org/10.1016/j.jcf.2019.01.013>

[6. Hoefnagels JW, Fischer K, Bos RAT, Driessens MHE, Meijer SLA, Schutgens REG, et al. A feasibility study on two tailored interventions to improve adherence in adults with haemophilia. Pilot Feasibility Stud [Internet]. 2020;6:189. Available from:](http://paperpile.com/b/Qzhbp1/Pcwfm) <http://dx.doi.org/10.1186/s40814-020-00723-w>

[7. Jackson AD, Kirwan L, Gibney S, Jeleniewska P, Fletcher G, Doyle G. Associations between health literacy and patient outcomes in adolescents and young adults with cystic fibrosis. Eur J Public Health [Internet]. 2020;30:112–8. Available from:](http://paperpile.com/b/Qzhbp1/wzXHk) <http://dx.doi.org/10.1093/eurpub/ckz148>

[8. Ringqvist K, Borg K, Möller MC. Tolerability and psychological effects of a multimodal day-care rehabilitation program for persons with Huntington’s disease. J Rehabil Med [Internet]. 2021;53:jrm00143. Available from:](http://paperpile.com/b/Qzhbp1/Jy23I) <http://dx.doi.org/10.2340/16501977-2748>

[9. Stubberud J, Langenbahn D, Levine B, Stanghelle J, Schanke A-K. Emotional health and coping in spina bifida after goal management training: a randomized controlled trial. Rehabil Psychol [Internet]. 2015;60:1–16. Available from:](http://paperpile.com/b/Qzhbp1/9HEKo) <http://dx.doi.org/10.1037/rep0000018>

[10. Torres-Ortuño A, Cuesta-Barriuso R, Nieto-Munuera J, Galindo-Piñana P, López-Pina J-A. The behaviour and perception of illness: modulating variables of adherence in patients with haemophilia. Vox Sang [Internet]. 2018; Available from:](http://paperpile.com/b/Qzhbp1/0dVRx) <http://dx.doi.org/10.1111/vox.12669>

[11. Ioannou L, Massie J, Collins V, McClaren B, Delatycki MB. Population-based genetic screening for cystic fibrosis: attitudes and outcomes. Public Health Genomics [Internet]. 2010;13:449–56. Available from:](http://paperpile.com/b/Qzhbp1/Ss4O9) <http://dx.doi.org/10.1159/000276544>

[12. Rovira-Moreno E, Abuli A, Codina-Sola M, Valenzuela I, Serra-Juhe C, Cuscó I, et al. Beyond the disease itself: A cross-cutting educational initiative for patients and families with rare diseases. J Genet Couns [Internet]. 2021;30:693–700. Available from:](http://paperpile.com/b/Qzhbp1/xNhA3) <http://dx.doi.org/10.1002/jgc4.1354>

[13. Beinke K, O’Callaghan F, Morrissey S. Illness Perceptions of Cystic Fibrosis: A Comparison of Young Adults with CF and Same-Aged Peers. Behav Med [Internet]. 2017;43:40–6. Available from:](http://paperpile.com/b/Qzhbp1/52hJF) <http://dx.doi.org/10.1080/08964289.2015.1045824>

[14. Katavic SS, Tanackovic SF, Badurina B. Illness perception and information behaviour of patients with rare chronic diseases. Inflamm Res [Internet]. 2016 [cited 2023 Jun 14];21. Available from:](http://paperpile.com/b/Qzhbp1/R3bXF) <http://dx.doi.org/10.1111/hir.12261>

[15. Bhatt N, Boggio L, Simpson ML. Using an educational intervention to assess and improve disease-specific knowledge and health literacy and numeracy in adolescents and young adults with haemophilia A and B. Haemophilia [Internet]. 2021;27:229–36. Available from:](http://paperpile.com/b/Qzhbp1/0CZrE) <https://onlinelibrary.wiley.com/doi/abs/10.1111/hae.14228>

[16. Dwyer AA, Quinton R, Morin D, Pitteloud N. Identifying the unmet health needs of patients with congenital hypogonadotropic hypogonadism using a web-based needs assessment: implications for online interventions and peer-to-peer support. Orphanet J Rare Dis [Internet]. 2014;9:83. Available from:](http://paperpile.com/b/Qzhbp1/dcUZH) <https://ojrd.biomedcentral.com/articles/10.1186/1750-1172-9-83>

[17. Merker VL, McDannold S, Riklin E, Talaei-Khoei M, Sheridan MR, Jordan JT, et al. Health literacy assessment in adults with neurofibromatosis: electronic and short-form measurement using FCCHL and Health LiTT. J Neurooncol [Internet]. 2018;136:335–42. Available from:](http://paperpile.com/b/Qzhbp1/FTkFY) <http://dx.doi.org/10.1007/s11060-017-2657-8>

[18. Riklin E, Talaei-Khoei M, Merker VL, Sheridan MR, Jordan JT, Plotkin SR, et al. First report of factors associated with satisfaction in patients with neurofibromatosis. Am J Med Genet A [Internet]. 2017;173:671–7. Available from:](http://paperpile.com/b/Qzhbp1/is7Oo) <http://dx.doi.org/10.1002/ajmg.a.38079>

[19. Parvizi MM, Lankarani KB, Handjani F, Ghahramani S, Parvizi Z, Rousta S. Health literacy in patients with epidermolysis bullosa in Iran. J Educ Health Promot [Internet]. 2017;6:105. Available from:](http://paperpile.com/b/Qzhbp1/A38fZ) <http://dx.doi.org/10.4103/jehp.jehp_64_17>

[20. Lewis KL, John B, Condren M, Carter SM. Evaluation of Medication-related Self-care Skills in Patients With Cystic Fibrosis. J Pediatr Pharmacol Ther [Internet]. 2016;21:502–11. Available from:](http://paperpile.com/b/Qzhbp1/3b8df) <http://dx.doi.org/10.5863/1551-6776-21.6.502>

[21. Lonabaugh KP, O’Neal KS, McIntosh H, Condren M. Cystic fibrosis-related education: Are we meeting patient and caregiver expectations? Patient Educ Couns [Internet]. 2018;101:1865–70. Available from:](http://paperpile.com/b/Qzhbp1/806uw) <http://dx.doi.org/10.1016/j.pec.2018.06.004>

[22. O’Mahar K, Holmbeck GN, Jandasek B, Zukerman J. A camp-based intervention targeting independence among individuals with spina bifida. J Pediatr Psychol [Internet]. 2010;35:848–56. Available from:](http://paperpile.com/b/Qzhbp1/lQoo6) <http://dx.doi.org/10.1093/jpepsy/jsp125>

[23. Raphaelis S, Mayer H, Ott S, Hornung R, Senn B. Effects of Written Information and Counseling on Illness-Related Uncertainty in Women With Vulvar Neoplasia. Oncol Nurs Forum [Internet]. 2018;45:748–60. Available from:](http://paperpile.com/b/Qzhbp1/nzkQ0) <http://dx.doi.org/10.1188/18.ONF.748-760>

[24. Rosnau K, Hashmi SS, Northrup H, Slopis J, Noblin S, Ashfaq M. Knowledge and Self-Esteem of Individuals with Neurofibromatosis Type 1 (NF1). J Genet Couns [Internet]. 2017;26:620–7. Available from:](http://paperpile.com/b/Qzhbp1/Xhu60) <http://dx.doi.org/10.1007/s10897-016-0036-9>

[25. Shoshan L, Ben-Zvi D, Meyer S, Katz-Leurer M. Sexuality in relation to independence in daily functions among young people with spina bifida living in Israel. Rehabil Nurs [Internet]. 2012;37:11–7; quiz 17–8. Available from:](http://paperpile.com/b/Qzhbp1/ujeo4) <http://dx.doi.org/10.1002/RNJ.00002>

[26. Skirton H, Williams JK, Jackson Barnette J, Paulsen JS. Huntington disease: families’ experiences of healthcare services. J Adv Nurs [Internet]. 2010;66:500–10. Available from:](http://paperpile.com/b/Qzhbp1/7oajv) <http://dx.doi.org/10.1111/j.1365-2648.2009.05217.x>
