# Supplementary material for: A scoping review of health literacy in rare disorders: key issues and research directions
Source: Orphanet J Rare Dis. 2024 Sep 6;19:328. doi: 10.1186/s13023-024-03332-5 (PMC11380335; doi:10.1186/s13023-024-03332-5)
Supplement: Supplementary file 7 — Supplementary Material 7 [file 13023_2024_3332_MOESM7_ESM.docx]

Additional file 7

Table 5. Study-specific outcome measures.

| Outcome | Diagnosis | Reference |
| --- | --- | --- |
| Knowledge levels, needs and gaps | Haemophilia | Arnold, 2014 [[1]](https://paperpile.com/c/lgSrJa/vElJV) |
| Health numeracy | Haemophilia | Bhatt, 2021 [[2]](https://paperpile.com/c/lgSrJa/AQINf) |
| Relevance and availability of information and support | Huntington | Braisch 2016 [[3]](https://paperpile.com/c/lgSrJa/amYgo) |
| Medication information sources | Vasculitis | Carpenter, 2011 [[4]](https://paperpile.com/c/lgSrJa/Iyllx) |
| Satisfaction with care transition | Cystic Fibrosis | Chaudhry 2013 [[5]](https://paperpile.com/c/lgSrJa/sdgQO) |
| Views and attitudes to using digital tools in patient registries | Myotonic Dystrophy | Coathup 2016 [[6]](https://paperpile.com/c/lgSrJa/HGhX5) |
| Degree of education and empowerment | Haemophilia | De la Corte-Rodriguez 2019 [[7]](https://paperpile.com/c/lgSrJa/Q9bb2) |
| Reasons for non-participation support groups | Systemic Sclerosis | Delisle 2016 [[8]](https://paperpile.com/c/lgSrJa/cFkRL) |
| Self-rated health | Spina Bifida and Spinar Cord Injury | Dicianno 2016 [[9]](https://paperpile.com/c/lgSrJa/W2sat) |
| Knowledge about preventable conditions | Spina Bifida and Spinar Cord Injury | Dicianno 2016  [[9]](https://paperpile.com/c/lgSrJa/W2sat) |
| Health information-seeking patterns | Congenital hypogonadotropic hypogonadism | Dwyer 2014 [[10]](https://paperpile.com/c/lgSrJa/Oj7kw) |
| Interactions with healthcare system/providers | Congenital hypogonadotropic hypogonadism | Dwyer 2014 [[10]](https://paperpile.com/c/lgSrJa/Oj7kw) |
| Adherence to treatment/healthcare | Congenital hypogonadotropic hypogonadism | Dwyer 2014 [[10]](https://paperpile.com/c/lgSrJa/Oj7kw) |
| Reasons for screening, knowledge of CF, recollection, understanding/impact of carrier status and communication of results to family | Cystic fibrosis | Ioannou 2010 [[11]](https://paperpile.com/c/lgSrJa/y7ShV) |
| Health information-seeking behaviour | Rare diagnoses (=systemic lupus erythematous, Scleroderma and myasthenia gravis) | Katavic 2016 [[12]](https://paperpile.com/c/lgSrJa/0jwFF) |
| Information avoidance | Rare diagnoses (=systemic lupus erythematous, Scleroderma and myasthenia gravis) | Katavic 2016 [[12]](https://paperpile.com/c/lgSrJa/0jwFF) |
| (1) Self-assessed knowledge of DM1, (2) perception of disease severity, (3) knowledge of the DM1 mode of inheritance, and (4) evaluation of the frequency of impairments/disabilities characteristic of DM1 | Myotonic Dystrophy type 1 (DM1) | Laberge 2010 [[13]](https://paperpile.com/c/lgSrJa/iYdAM) |
| Knowledge of haemophilia and management of hemarthrosis | Haemophilia | Le Doré 2021 [[14]](https://paperpile.com/c/lgSrJa/3HOIQ) |
| Knowledge of disease and treatment, perceptions of their disease and contact with healthcare facilities | Haemophilia | Lindvall 2010 [[15]](https://paperpile.com/c/lgSrJa/aaC0R) |
| 1) Received CF education, 2) confidence in CF-related knowledge, 3) educational sources 4) at what age educational goals related to CF should be met. | Cystic fibrosis | Lonabaugh 2018 [[16]](https://paperpile.com/c/lgSrJa/ragAS) |
| Knowledge, attitude and behaviour towards their condition. | Haemophilia | Mohan 2020 [[17]](https://paperpile.com/c/lgSrJa/AxWpd) |
| Knowledge of the patients on home treatment | Haemophilia | Mulders 2012 [[18]](https://paperpile.com/c/lgSrJa/uS7x6) |
| Type of diagnosis, experiences of diagnosis/symptoms, information received, information preferences, experiences of healthcare services | People with rare diagnosis | Molster 2016 [[19]](https://paperpile.com/c/lgSrJa/yIFjo) |
| Individual Spina Bifida and Social Goals | Spina Bifida | O`Mahar 2010 [[20]](https://paperpile.com/c/lgSrJa/pLbig) |
| Sharing of Spina Bifida Management Responsibilities | Spina Bifida | O`Mahar 2010 [[20]](https://paperpile.com/c/lgSrJa/pLbig) |
| Adherence to treatment (both to HCP recommendations and to own self-reported understanding of treatment recommendations) | Cystic Fibrosis | Pakhale 2016 [[21]](https://paperpile.com/c/lgSrJa/Z409I) |
| Experience with and Knowledge of NF1 | Neurofibromatosis 1 (NF1) | Rosnau K 2017 [[22]](https://paperpile.com/c/lgSrJa/dRNrq) |
| Genetic knowledge | Rare disorders | Rovira-Moreno  2020 [[23]](https://paperpile.com/c/lgSrJa/VVSz4) |
| Connectedness | Familial chylomicronemia syndrome | Salvatore  2018 [[24]](https://paperpile.com/c/lgSrJa/zSI4B) |
| Symptom severity | Familial chylomicronemia syndrome | Salvatore  2018 [[24]](https://paperpile.com/c/lgSrJa/zSI4B) |
| Fragile X-related health knowledge, and resource use | Women who carry a fragile X premutation | Smolich 2020 [[25]](https://paperpile.com/c/lgSrJa/IExTJ) |

[1. Arnold E, Lane S, Webert KE, Chan A, Walker I, Tufts J, et al. What should men living with haemophilia need to know? The perspectives of Canadian men with haemophilia. Haemophilia [Internet]. 2014;20:219–25. Available from:](http://paperpile.com/b/lgSrJa/vElJV) <https://onlinelibrary.wiley.com/doi/10.1111/hae.12297>

[2. Bhatt N, Boggio L, Simpson ML. Using an educational intervention to assess and improve disease-specific knowledge and health literacy and numeracy in adolescents and young adults with haemophilia A and B. Haemophilia [Internet]. 2021;27:229–36. Available from:](http://paperpile.com/b/lgSrJa/AQINf) <https://onlinelibrary.wiley.com/doi/abs/10.1111/hae.14228>

[3. Braisch U, Martinez-Horta S, MacDonald M, Orth M. Important but not enough - information about HD related topics and peer and professional support for young adults from HD families. J Huntingtons Dis [Internet]. 2016 [cited 2023 Jun 6];5:379–87. Available from:](http://paperpile.com/b/lgSrJa/amYgo) <https://content.iospress.com/articles/journal-of-huntingtons-disease/jhd160218>

[4. Carpenter DM, DeVellis RF, Hogan SL, Fisher EB, DeVellis BM, Jordan JM. Use and perceived credibility of medication information sources for patients with a rare illness: differences by gender. J Health Commun [Internet]. 2011;16:629–42. Available from:](http://paperpile.com/b/lgSrJa/Iyllx) <http://dx.doi.org/10.1080/10810730.2011.551995>

[5. Chaudhry SR, Keaton M, Nasr SZ. Evaluation of a cystic fibrosis transition program from pediatric to adult care. Pediatr Pulmonol [Internet]. 2013;48:658–65. Available from:](http://paperpile.com/b/lgSrJa/sdgQO) <http://dx.doi.org/10.1002/ppul.22647>

[6. Coathup V, Teare HJA, Minari J, Yoshizawa G, Kaye J, Takahashi MP, et al. Using digital technologies to engage with medical research: views of myotonic dystrophy patients in Japan. BMC Med Ethics [Internet]. 2016;17:51. Available from:](http://paperpile.com/b/lgSrJa/HGhX5) <http://dx.doi.org/10.1186/s12910-016-0132-2>

[7. De la Corte-Rodriguez H, Rodriguez-Merchan EC, Alvarez-Roman T, Martin-Salces M, Garcia-Barcenilla S, Jimenez-Yuste V. Health education and empowerment in adult patients with haemophilia. Expert Rev Hematol [Internet]. 2019;12:989–95. Available from:](http://paperpile.com/b/lgSrJa/Q9bb2) <http://dx.doi.org/10.1080/17474086.2019.1650640>

[8. Delisle VC, Gumuchian ST, Pelaez S, Malcarne VL, El-Baalbaki G, Körner A, et al. Reasons for non-participation in scleroderma support groups. Clin Exp Rheumatol [Internet]. 2016;34 Suppl 100:56–62. Available from:](http://paperpile.com/b/lgSrJa/cFkRL) <https://www.ncbi.nlm.nih.gov/pubmed/26950221>

[9. Dicianno BE, Lovelace J, Peele P, Fassinger C, Houck P, Bursic A, et al. Effectiveness of a Wellness Program for Individuals With Spina Bifida and Spinal Cord Injury Within an Integrated Delivery System. Arch Phys Med Rehabil [Internet]. 2016;97:1969–78. Available from:](http://paperpile.com/b/lgSrJa/W2sat) <http://dx.doi.org/10.1016/j.apmr.2016.05.014>

[10. Dwyer AA, Quinton R, Morin D, Pitteloud N. Identifying the unmet health needs of patients with congenital hypogonadotropic hypogonadism using a web-based needs assessment: implications for online interventions and peer-to-peer support. Orphanet J Rare Dis [Internet]. 2014;9:83. Available from:](http://paperpile.com/b/lgSrJa/Oj7kw) <https://ojrd.biomedcentral.com/articles/10.1186/1750-1172-9-83>

[11. Ioannou L, Massie J, Collins V, McClaren B, Delatycki MB. Population-based genetic screening for cystic fibrosis: attitudes and outcomes. Public Health Genomics [Internet]. 2010;13:449–56. Available from:](http://paperpile.com/b/lgSrJa/y7ShV) <http://dx.doi.org/10.1159/000276544>

[12. Katavic SS, Tanackovic SF, Badurina B. Illness perception and information behaviour of patients with rare chronic diseases. Inflamm Res [Internet]. 2016 [cited 2023 Jun 14];21. Available from:](http://paperpile.com/b/lgSrJa/0jwFF) <http://dx.doi.org/10.1111/hir.12261>

[13. Laberge L, Prévost C, Perron M, Mathieu J, Auclair J, Gaudreault M, et al. Clinical and genetic knowledge and attitudes of patients with myotonic dystrophy type 1. Public Health Genomics [Internet]. 2010;13:424–30. Available from:](http://paperpile.com/b/lgSrJa/iYdAM) <http://dx.doi.org/10.1159/000316238>

[14. le Doré S, Grinda N, Ferré E, Roussel-Robert V, Frotscher B, Chamouni P, et al. The hemarthrosis-simulating knee model: A useful tool for individualized education in patients with hemophilia (GEFACET study). J Blood Med [Internet]. 2021;12:133–8. Available from:](http://paperpile.com/b/lgSrJa/3HOIQ) <https://www.tandfonline.com/doi/abs/10.2147/JBM.S280032>

[15. Lindvall K, Colstrup L, Loogna K, Wollter I, Grönhaug S. Knowledge of disease and adherence in adult patients with haemophilia. Haemophilia [Internet]. 2010;16:592–6. Available from:](http://paperpile.com/b/lgSrJa/aaC0R) <http://dx.doi.org/10.1111/j.1365-2516.2009.02189.x>

[16. Lonabaugh KP, O’Neal KS, McIntosh H, Condren M. Cystic fibrosis-related education: Are we meeting patient and caregiver expectations? Patient Educ Couns [Internet]. 2018;101:1865–70. Available from:](http://paperpile.com/b/lgSrJa/ragAS) <http://dx.doi.org/10.1016/j.pec.2018.06.004>

[17. Mohan R, Radhakrishnan N, Varadarajan M, Anand S. Assessing the current knowledge, attitude and behaviour of adolescents and young adults living with haemophilia. Haemophilia [Internet]. 2021;27:e180–6. Available from:](http://paperpile.com/b/lgSrJa/AxWpd) <http://dx.doi.org/10.1111/hae.14229>

[18. Mulders G, de Wee EM, Vahedi Nikbakht-Van de Sande MCVM, Kruip MJHA, Elfrink EJ, Leebeek FWG. E-learning improves knowledge and practical skills in haemophilia patients on home treatment: a randomized controlled trial. Haemophilia [Internet]. 2012;18:693–8. Available from:](http://paperpile.com/b/lgSrJa/uS7x6) <https://onlinelibrary.wiley.com/doi/10.1111/j.1365-2516.2012.02786.x>

[19. Molster C, Urwin D, Di Pietro L, Fookes M, Petrie D, van der Laan S, et al. Survey of healthcare experiences of Australian adults living with rare diseases. Orphanet J Rare Dis [Internet]. 2016;11:30. Available from:](http://paperpile.com/b/lgSrJa/yIFjo) <http://dx.doi.org/10.1186/s13023-016-0409-z>

[20. O’Mahar K, Holmbeck GN, Jandasek B, Zukerman J. A camp-based intervention targeting independence among individuals with spina bifida. J Pediatr Psychol [Internet]. 2010;35:848–56. Available from:](http://paperpile.com/b/lgSrJa/pLbig) <http://dx.doi.org/10.1093/jpepsy/jsp125>

[21. Pakhale S, Baron J, Armstrong M, Tasca G, Gaudet E, Aaron SD, et al. Lost in translation? How adults living with Cystic Fibrosis understand treatment recommendations from their healthcare providers, and the impact on adherence to therapy. Patient Educ Couns [Internet]. 2016;99:1319–24. Available from:](http://paperpile.com/b/lgSrJa/Z409I) <http://dx.doi.org/10.1016/j.pec.2016.03.023>

[22. Rosnau K, Hashmi SS, Northrup H, Slopis J, Noblin S, Ashfaq M. Knowledge and Self-Esteem of Individuals with Neurofibromatosis Type 1 (NF1). J Genet Couns [Internet]. 2017;26:620–7. Available from:](http://paperpile.com/b/lgSrJa/dRNrq) <http://dx.doi.org/10.1007/s10897-016-0036-9>

[23. Rovira-Moreno E, Abuli A, Codina-Sola M, Valenzuela I, Serra-Juhe C, Cuscó I, et al. Beyond the disease itself: A cross-cutting educational initiative for patients and families with rare diseases. J Genet Couns [Internet]. 2021;30:693–700. Available from:](http://paperpile.com/b/lgSrJa/VVSz4) <http://dx.doi.org/10.1002/jgc4.1354>

[24. Salvatore V, Gilstrap A, Williams KR, Thorat S, Stevenson M, Gwosdow AR, et al. Evaluating the impact of peer support and connection on the quality of life of patients with familial chylomicronemia syndrome. Expert Opinion on Orphan Drugs [Internet]. 2018;6:497–505. Available from:](http://paperpile.com/b/lgSrJa/zSI4B) <https://doi.org/10.1080/21678707.2018.1505495>

[25. Smolich L, Charen K, Sherman SL. Health knowledge of women with a fragile X premutation: Improving understanding with targeted educational material. J Genet Couns [Internet]. 2020;29:983–91. Available from:](http://paperpile.com/b/lgSrJa/IExTJ) <http://dx.doi.org/10.1002/jgc4.1222>
