# Supplementary material for: A scoping review of health literacy in rare disorders: key issues and research directions
Source: Orphanet J Rare Dis. 2024 Sep 6;19:328. doi: 10.1186/s13023-024-03332-5 (PMC11380335; doi:10.1186/s13023-024-03332-5)
Supplement: Supplementary file 8 — Supplementary Material 8 [file 13023_2024_3332_MOESM8_ESM.docx]

Additional file 8

Table 6. Description of interventions.

| First Author  Year Country | Target group | Aim of the Intervention | Structure, content and setting | Perceived benefits or effects |
| --- | --- | --- | --- | --- |
| Bhatt  2021  USA [[58]](https://paperpile.com/c/G34cCo/T55i5) | People with haemophilia A or B | To improve knowledge, health literacy, health numeracy, adherence and joint health. | Individual education using plain language and audiovisual material. Using the ‘inform, ask, listen, inform again, ask again’-model, according to the teach-back method. | Participants with low health literacy and/or health numeracy may benefit from alternate methods of education such as audiovisual material.  Education using audiovisual materials improved knowledge and health numeracy in this study; however, this did not affect adherence to the prescribed therapy. |
| Bogart  2017  USA [[70]](https://paperpile.com/c/G34cCo/QvQhr) | People with Moebius syndrome | To provide informational, instrumental and emotional support. | 3-day support conference with informational support (through formal presentations), available private consultations, discussion groups and social events. | Destigmatizing social companionship and informational support were the most frequently mentioned benefits of attending. |
| Chaleat-Valayer et al. 2019 France [[67]](https://paperpile.com/c/G34cCo/zj1Mo) | People with hypermobile Ehlers-Danlos syndrome | To raise awareness and provide information, learning strategies, and psychosocial support regarding syndrome, treatment, care, hospitalization and other care structures, as well as health or disease behaviors. | 5 half days or 5 days workshops with therapeutic group-education in a hospital setting. | The intervention was a success in terms of recognition and satisfaction and led to an increase in knowledge and adaptive skills. |
| Chaudhry  2013  USA [[28]](https://paperpile.com/c/G34cCo/05r6t) | People with Cystic Fibrosis | To maximize lifelong functioning and well-being, and to reduce interruption of care. | Transition program for adolescents transferring to adult center. Including coordination and preparation between the pediatric Center, the patients and families and the adult Center. The program allows patients to engage with the adult team before the transition, and addresses issues specific to Cystic Fibrosis. | Structured transition programs seem to enhance patient satisfaction, perceived health status, and independence. However, they do not appear to decrease patient anxiety. |
| Depping  2021  Germany [[69]](https://paperpile.com/c/G34cCo/mzEmI) | People with rare chronic diseases | To increase acceptance of the disease.  And to heighten rates in  coping strategies, improved illness perceptions and cognitions,  and higher quality of life and social support, as well as  reduced depression, anxiety, and somatic symptom severity. | 6-week intervention with a self-help book and telephone-based peer counselling. | Six months after the intervention, the intervention group had significantly higher rates of acceptance of the disease.  Several secondary outcomes, including different coping strategies, social support, and mental quality of life, were significantly higher after the intervention. |
| Dicianno  2016  USA [[73]](https://paperpile.com/c/G34cCo/EYl72) | People with Spina Bifida and Spinal Cord Injury | To improve health outcomes and patient experience of care, result in a positive return on investment and provide evidence for scalability. | Evidence-based wellness program consisting of care coordination from a mobile nurse, patient education, and patient incentives. | Improvements in all main outcome measures were seen after 2 years of enrolment.  Although cost in year 1 of enrolment increased because of hospitalizations and the overall result of investment was negative, a small positive result of investment was seen in year 2 of enrolment. |
| Hoefnagels  2020  The Netherlands [[59]](https://paperpile.com/c/G34cCo/gH9Fx) | People with severe haemophilia A | Intervention 1 “Living with haemophilia”: improving illness acceptance and higher adherence to prophylaxis.  Intervention 2 “Challenging your haemophilia”: improving self-management higher adherence to prophylaxis. | Intervention 1: “Living with haemophilia”:  Seven 2-hour sessions and one follow-up session after 6 months with face-to-face group training supervised by two Acceptance and Commitment Therapy-qualified haemophilia healthcare professionals.  Intervention 2: “Challenging your haemophilia”: Online training with 5 mandatory and 3 additional modules (each taking approximately 45 min) to be completed within 2 months. | The preliminary results were promising as adherence, quality of life and illness perception all improved. The online training was however determined because of difficulties with enrolment, recruitment, and retention. |
| Le Doré et al. 2021. France [[50]](https://paperpile.com/c/G34cCo/qTviq) | People with severe/ moderate haemophilia | To improve patients’ knowledge, care and self-treatment skills. | Therapeutic training course at haemophilia clinics. Using a hemarthrosis-simulating artificial knee to visualize hemarthrosis on the anatomy of the knee joint and the impact of treatment. | An individualized training course can enhance the understanding of haemophilia in patients of all ages, especially in children and teenagers. Using an artificial knee model may assist in improving patients’ management of their disease. |
| Mulders  2012  The Netherlands [[56]](https://paperpile.com/c/G34cCo/CH43I) | People with haemophilia | To improve knowledge and skills. | An e-learning program lasting 15–30 min. | In patients with haemophilia, who are on home treatment, knowledge of haemophilia treatment and complications as well as practical skills can be improved by an educational e-learning program. |
| O`Mahar 2010  USA [[90]](https://paperpile.com/c/G34cCo/KYaXA) | People with Spina Bifida | To increase  independence. | One-week overnight summer camp with collaborative (i.e., parent and camper) goal identification, group sessions consisting of psycho-education and cognitive tools, and goal monitoring by camp counsellors. | Results indicated that significant progress was made on individually oriented goals from pre- to post-camp. |
| Raphaelis  2018  Switzerland and Austria [[93]](https://paperpile.com/c/G34cCo/nqwTW) | People with vulvar neoplasia | To decrease illness-related uncertainty. | Intervention 1: Written information, received as a set of leaflets.  Intervention 2: Counselling group  5 consultations with advanced practice nurses focusing on symptom self-management, healthcare services, and decision making. | Total uncertainty and the subscales of ambiguity, inconsistency, and unpredictability improved significantly over time within the counselling group but not within the written information group.  In addition, counselling improved inconsistency over time, and total uncertainty, inconsistency, and unpredictability at distinct time points more efficiently than written information. |
| Ringqvist  2021  Sweden [[64]](https://paperpile.com/c/G34cCo/uaA7y) | People with Huntington’s disease | To reduce psychiatric symptoms, improve health-related quality of life, and psychological health factors. | 8-week day-care rehabilitation programme including information and education, group counselling, physical training, speech therapy and creative activities given by a team of specialists. Some individual sessions if pronounced psychiatric symptoms. | Patients were satisfied and displayed significantly reduced anxiety and depression and improved health-related quality of life after rehabilitation.  A sense of coherence seems to be related to attendance rate, indicating that efforts to make life more understandable, manageable and meaningful for people with HD might increase participation in treatments. |
| Rovira-Moreno  2020 Spain [[86]](https://paperpile.com/c/G34cCo/d97sF) | People with rare disorders | To improve understanding of pathologies, empower patients to help them manage socio-cultural challenges and to better cope in their everyday life. | Education training program consisting of eight 2,5-hour-group sessions (five with basic medical knowledge and three covering psychosocial aspects of rare disorders) given by professionals and specialists working in the field of rare disorders in a University Hospital. | The results show improvements in knowledge and better management of the psychological impact.  The mean score for anxiety decreased but was not statistically significant.  Participants were able to exchange experiences and concerns, most of which were shared even though the RDs were different.  Overall, the program was evaluated by the participants as a highly beneficial experience and all of them were interested in attending advanced editions. |
| Smolich  US  2020 [[47]](https://paperpile.com/c/G34cCo/7VgqD) | People that are carriers of a fragile X permutation | To improve fragile X premutation-related health knowledge. | Participants were emailed the 29-page informational booklet ‘Women's Health and the Fragile X Premutation’. | Scores were significantly increased after receipt of the booklet. Participants answered that the booklet was ‘very helpful’ (44.6%) or ‘somewhat helpful’ (38.5%). Although the booklet provided women with needed information, gaps in knowledge still existed. |
| Stubberud  2015  Norway [[92]](https://paperpile.com/c/G34cCo/weAuZ) | People with Spina Bifida | To improve emotional health and coping. | Goal management training is a cognitive rehabilitation method developed to improve executive functioning. It uses strategies such as stopping and orienting to relevant information, partitioning goals into subgoals, encoding and retaining goals, monitoring performance and use of mindfulness training. Group treatment. | Findings indicated positive effects of goal management training on measures of emotional health. The goal management training group showed significant improvement, compared with control subjects, on a self-report inventory of depressive and anxiety symptoms after training, lasting at least 6 months posttreatment. Furthermore, both groups showed improvements after training on mental health components of health-related quality of life. Finally, the goal management training group showed a significant increase in task-focused coping and a decrease in avoidant coping after training compared with pretreatment baseline assessment scores. |
| Van Balen et al. 2018 Canada (OBS authors from the Netherlands, study conducted in Canada) [[57]](https://paperpile.com/c/G34cCo/WlxHN) | People with haemophilia | To change the patient's understanding of their disease and encourage them to make treatment decisions. | Meeting with members of the treatment team (haematologist, nursing specialist and physiotherapist) at a patient‐centred prophylaxis clinic.The treatment team presented visual information on an individual's pharmacokinetic profile and bleed history and encouraged patients to participate in treatment. | Participants perceived the approach helpful because it enhanced communication with the Clinic Team, increased their understanding of haemophilia and pharmacokinetics of coagulation factor and facilitated treatment decisions. |
