# Supplementary material for: A scoping review of health literacy in rare disorders: key issues and research directions
Source: Orphanet J Rare Dis. 2024 Sep 6;19:328. doi: 10.1186/s13023-024-03332-5 (PMC11380335; doi:10.1186/s13023-024-03332-5)
Supplement: Supplementary file 9 — Supplementary Material 9 [file 13023_2024_3332_MOESM9_ESM.docx]

Additional file 9.

Table 2. Country distribution and number of reports

| Country and number of articles |  |
| --- | --- |
| Country | No. |
| USA | 21 |
| Canada | 11 |
| UK | 8 |
| Australia | 4 |
| France | 4 |
| Germany | 3 |
| Spain | 3 |
| Croatia | 2 |
| Ireland | 2 |
| Italy | 2 |
| Japan | 2 |
| Sweden | 2 |
| The Netherlands | 2 |
| India | 1 |
| Iran | 1 |
| Israel | 1 |
| Mexico | 1 |
| Norway | 1 |
| Poland | 1 |
| Sweden, Norway and Denmark | 1 |
| Switzerland | 1 |
| Switzerland and Austria | 1 |
| Total | 75 |
